# Observational and Genetic Associations of Modifiable Risk Factors with Aortic Valve Stenosis: A Prospective Cohort Study of 0.5 Million Participants

**DOI:** 10.3390/nu14112273

**Published:** 2022-05-28

**Authors:** Ninghao Huang, Zhenhuang Zhuang, Zhonghua Liu, Tao Huang

**Affiliations:** 1 Department of Epidemiology & Biostatistics, School of Public Health, Peking University, Beijing 100191, China; ninghaohuang@bjmu.edu.cn (N.H.); 1510306133@pku.edu.cn (Z.Z.); 2 Department of Statistics and Actuarial Science, The University of Hong Kong, Hong Kong 999077, China; zhhliu@hku.hk; 3 Key Laboratory of Molecular Cardiovascular Sciences, Ministry of Education, Peking University, Beijing 100871, China; 4 Center for Intelligent Public Health, Institute for Artificial Intelligence, Peking University, Beijing 100871, China

**Keywords:** aortic valve stenosis, Mendelian randomization, obesity, sleep, biochemical measures

## Abstract

Background: Observational studies have shown that modifiable risk factors are associated with aortic valve stenosis (AVS). However, the causality behind these associations remains largely unknown. Objectives: To explore the associations of modifiable risk factors, including metabolic factors, biochemical measures, education, and lifestyles with AVS and their potential causal associations. Methods: We enrolled 361,930 British white people with genetic data in the UK biobank. Cox proportional risk regression models were used to estimate the hazard ratios between 28 modifiable risk factors and AVS. We used genetic instruments for modifiable risk factors to determine the potential causal relationships using a one-sample Mendelian randomization (MR) approach. Results: A total of 1602 participants developed AVS during an 8.4-year follow-up. Observational analyses showed higher adiposity, blood pressure, heart rate, low-density lipoprotein, urate, C-reactive protein, creatinine, albumin, and glycated hemoglobin, but lower serum vitamin D, and education, unhealthy lifestyle, and poor sleep quality were related to a higher risk of AVS after adjusting for the Bonferroni correction (*p* < 0.0013). Genetically predicted 1-SD higher levels of body mass index [HR: 1.09, 95% CI: 1.03 to 1.16], body fat percentage (1.17, 1.03 to 1.33), triglyceride (TG) [1.08, 1.00 to 1.16], low-density lipoprotein (LDL) (1.15, 1.08 to 1.21) and serum total cholesterol (TC) (1.13, 1.02 to 1.25) were associated with a higher risk of AVS, respectively. Genetically determined per category higher insomnia (1.32, 1.13 to 1.55) was also associated with AVS. The abovementioned genetic associations with the incident AVS showed an increasing relationship pattern. Conclusions: This study provides strong evidence for the potential causal roles of cardiometabolic factors in developing AVS, highlighting that an idea of metabolic status through a healthy lifestyle may help prevent AVS.

## 1. Introduction

Aortic valve stenosis (AVS), a narrowing of the aortic valve opening that restricts the blood flow from the left ventricle to the aorta [1], has become one of the most common and severe valve diseases problems. Globally, about 40 million seniors aged more than 65 in 2020 suffered from AVS. The number is expected to be 72 million in 2030 [2]. Notably, the mortality rate of severe AVS has reached almost 50% within two years [3,4,5]. However, the causes of AVS are mainly unknown. Exploring the potential to reduce AVS morbidity and mortality by targeting modifiable risk factors is of great public health significance.

Conventional observational studies have shown that increasing ages [6], higher body mass index (BMI) [7], higher resting heart rate (RHR) [8,9,10], increased cigarettes consumption [11], much more coffee intake [12], weak blood pressure levels [13,14,15], and diabetes [13,14] were associated with increased risk of AVS. Moreover, large-scale prospective cohorts highlighted that AVS is associated with long-term exposure to high systolic blood pressure [16,17]. Growing evidence shows that the deposition of lipoproteins can induce AVS [18]. It is worth noting that available evidence from traditional observational studies, which are susceptible to confounding and reverse causation, generated inconclusive results. Spotting the cause and early warning signs of AVS, thus, altering risk factors or using drugs at an early stage, may reverse the process of malignant calcification in AVS [19].

Mendelian randomization (MR) has been widely accepted to explore the causal inference between risk factors and disease. MR uses randomly allocated genetic alleles as instrumental variables and avoids reverse causality bias in observational studies. This approach is used extensively in the biomedical field to decipher underlying causality [20]. Previously, several MR studies have reported obesity [21], smoking [22], insomnia [23], and blood lipid, such as low-density lipoprotein [LDL], triglyceride [TG], and total cholesterol [TC] [24,25], as causal factors for the development of AVS. However, it remains unclear whether conventional risk factors play causal roles in AVS, especially in other biochemical factors. Therefore, in the present study, we used an MR approach to explore the causal relationships between modifiable risk factors, including metabolic factors, biochemical factors, education situations, lifestyle factors, and AVS based on the UK Biobank (UKB) of 0.5 million participants, providing the basis for clinical and health management.

## 2. Methods

### 2.1. Study Design and Population

This study adhered to the guidelines for strengthening the reporting of observational studies in epidemiology using Mendelian randomization (STROBE-MR) [26]. The UKB study, including about 500,000 individuals aged between 40 and 69, is a prospective cohort study from the United Kingdom. Participants were invited to one of the twenty-two medical centers between 2006 and 2010 to collect biologic samples. Baseline information was recorded by touch screen questionnaires [27]. UKB genotype data, which included about 96 million variants in 487,381 participants, were imputed with IMPUTE4 by the UK10K+ 1000 Genomes panel and the Haplotype Reference Consortium. UKB has been approved by the Northwest Multi-centre Research Ethics Committee (MREC). All participants gave written informed consent before entering the cohort and were anonymized under analysis.

### 2.2. Measurement of Modifiable Risk Factors

We divided modifiable risk factors into five categories: metabolic factors, biochemical factors, education, and lifestyle factors. The modifiable risk factors we selected should meet the instrumental variable hypothesis (weak instrumental variable bias and linkage disequilibrium). Finally, we included 28 modifiable risk factors, including metabolic factors (BMI, body fat percentage (BF), the waist-hip ratio (WHR), systolic blood pressure (SBP), pulse pressure (PP), resting heart rate (RHR)), biochemical (glycated hemoglobin (HbA1c), and serum vitamin D (VD), TG, high-density lipoprotein (HDL), LDL, TC, urate, C-reactive protein (CRP), creatinine, albumin), education (age completed full-time education), and lifestyle factors (cigarettes consumption per day (CPD), smoking initiation, smoking cessation, coffee consumption, morningness, sleep duration, ease of getting up, napping, daytime dozing, snoring and insomnia). These modifiable risk factors were measured in baseline with a sample size greater than 315 thousand, except for years of education, daily smoking, and cessation of smoking, which are defined only in the population with a history of smoking (detailed information can been seen in Supplementary Table S3).

### 2.3. Measurement of Outcome

We defined the AVS with diagnosis and surgery using version 10 of the International Classification of Disease, which is I35 (nonrheumatic tricuspid (valve) stenosis, including bicuspid aortic valve). Participants were diagnosed by physicians using the hospital inpatients records.

### 2.4. Observational Analysis

Out of 502,528 participants, we excluded those with missing data for gender and age, those that were of non-white race and those with AVS and cancer at baseline, for 361,930 participants included. Cox proportional hazards models were conducted to explore associations between all modifiable hazards that met the proportional hazards assumption (*p* > 0.05). For AVS and modifiable risk factors, a follow-up time was defined as the time from the first measurement to the incidence of AVS or death, which came first. Multivariate models were adjusted for sex, age group (35–70 years, per 5 years increment), dichotomous for family history (included cardiovascular diseases and type 2 diabetes), education status (college or university degree, A levels/AS levels or equivalent, O levels/GCSEs or equivalent, CSEs or equivalent, NVQ or HND or HNC or equivalent, other professional qualifications), household income (less than 18,000 pounds per year (£/y), 18,000 to 29,999 £/y, 30,000 to 51,999 £/y, 52,000 to 100,000 £/y, more than 100,000 £/y), Townsend deprivation indices, metabolic equivalent tasks (METs), alcohol daily consumptions (grams), smoking status (never smoking, previous smoking, current smoking), and SBP (mmHg). We also adjusted with medication use of cholesterol-lowering for blood lipid factors. We excluded the participants without genotype data. Hazard ratios [HRs] and 95% confidence intervals [CIs] were used to evaluate AVS relationships and modifiable risk factors. The *p*-value threshold in the Cox models was adjusted by Bonferroni correction (adjusted *p* = 0.05/28 = 0.0018). To assess the robustness of the association, we excluded patients using blood-lipid lowering drugs and participants with less than three years of follow-up to rule out the potential effect of reverse causation in the sensitivity analysis.

### 2.5. Mendelian Randomization Analysis

We used individual data of single nucleotide polymorphic sites (SNPs) to calculate weighted genetic risk scores (GRS) for the two-stage least square regression MR of a single sample before conducting MR analysis. The SNPs corresponding to each modifiable risk factor were significant (*p*-value < 5 × 10^−8^) in their respective genome-wide association studies (GWASs), and the threshold value of linkage disequilibrium (LD) was set as 0.1 to ensure independence among SNPs [28]. SNPs that could not pass the LD threshold had been omitted in creating GRS. Details of GWASs [29,30,31,32,33,34,35,36,37,38,39,40,41] and SNPs’ sources can be seen in Supplementary Tables S1 and S2. The weighted GRS was calculated by the original effect size of the European race in summary data. Each SNP was imputed by mean and summed up after multiplying with its effect value, then divided half of the total of the effect size (for example, weighted GRSs = (β1 × SNP1 + β2 × SNP2 + … + βn × SNPn)/[(β1 + β2 + … + βn)/*n*]) [42,43]. Moreover, we defined or estimated the phenotypes of corresponding genotypes. Some phenotypes were derived using original variables, while others needed to be computed, and detailed information on phenotypes and their estimated methods were shown in Supplementary Table S3. 

**Figure 1:**
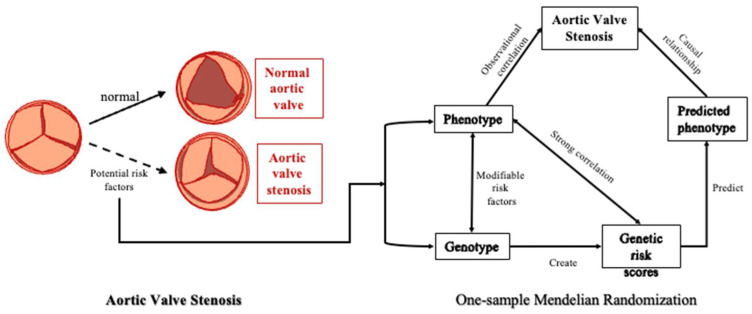
The Aortic Valve Stenosis Diagram. A normal tricuspid valve, known as the aortic valve, can open or close, depending on blood volume. An abnormal aortic valve that does not open properly can block blood flow and result in systemic ischemia. The procession of one-sample Mendelian randomization analysis. The three main steps of Mendelian randomization: Firstly, the genetic tool and the corresponding phenotype perform regression, which can only be carried out in the next step under significant circumstances; Secondly, the predicted value of phenotype was predicted using the regression results. Third, regression was performed using the predicted phenotypes and outcomes, and the statistically significant condition and direction of risk (consistent with the first step) were examined.

Then, we conducted the two-stage least square regression MR approach, which was used to examine the potential causal relationship between modifiable risk factors and AVS [44]. Firstly, linear regression was conducted after adjusting the covariates and the regression results, with the weighted GRSs of each modifiable risk factor taken as independent variables and their phenotypes as dependent variables. Predictor variables were then created, and the Z-score was standardized based on the previous step and then used to estimate HRs between incident AVS and the predictive modifiable risk factors (per unit reflecting one odd for each modifiable risk factor) by Cox models. We also used logistic regressions for all AVS participants (including incident and prevalent AVS) to test the robustness of the results. The procession of one-sample MR analysis is shown in Figure 1. Adjustments were made for sex, age, the first ten genetic principal components, and genotyping chip. Furthermore, we constructed various models with different covariables to check the stability of our results in the sensitivity analysis. The model incrementally adjusts for lifestyle and physiological indicators. We divided participants into quintile groups in categorical analyses according to their observational phenotypes and predicted variables. HRs were estimated with the lowest group as the reference group. We used trend charts to detect the relationships between the different groups of modifiable risk factors and AVS. The MR-Egger regression and weighted median methods were used in sensitivity analysis to test the robustness of potential causal association [26]. In addition, there is a low degree of bias due to horizontal pleiotropic (*p*-value of MR-egger intercepts > 0.05) [45]. We used F-statistic to examine the robustness of genetic instrumental variables. All analyses were conducted in STATA SE software (version 15) and R (version 4.1.2).

## 3. Results

### 3.1. Characteristics of Included Participants

Participants were followed for an average of 8.4 ± 1.6 years follow-up (2,885,764 person-years). Of these, a total of 1602 participants developed AVS at the end of the follow-up. Patients with AVS were older, more likely to be male (65.4%), and had more family history of cardiovascular diseases (62.7%) than non-AVS participants. In terms of metabolic factors, participants with AVS tended to have a greater obesity index; for example, the mean (±standard deviation) of BMI was 29.3 (±5.3) kg/m^2^ in AVS and 27.4 (±4.7) kg/m^2^ in non-AVS. Systolic blood pressures were higher in AVS (149.2 ± 20.8 mmHg) than in non-AVS (140 ± 19.6 mmHg). Similarly, AVS patients showed lower education levels and more unhealthy lifestyle behaviors. Patients also showed higher glycated hemoglobin (39.5 ± 11.5 mmol/mol vs. 35.9 ± 6.5 mmol/mol), and patients with AVS showed worse physiological status in other biochemical metabolites. Patients with AVS had poor sleep quality for sleep factors, including longer hours of sleep, more frequent naps and daytime sleepiness, and more significant snoring, as detailed in Table 1.

### 3.2. Observational Associations

**Figure 2:**
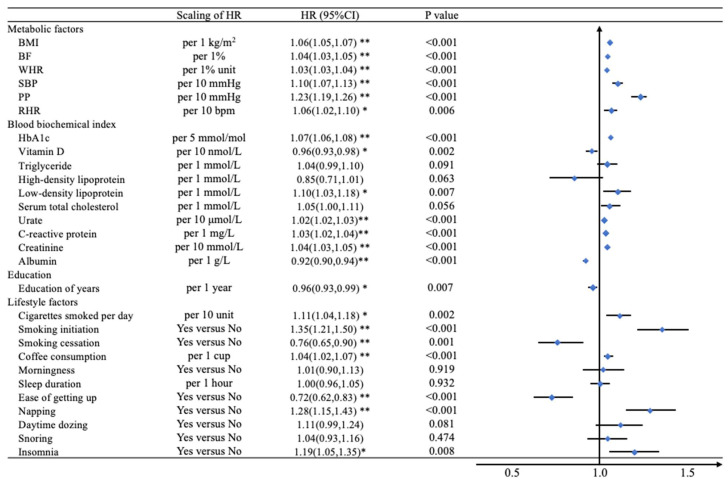
The hazard ratio of modifiable risk factors and AVS incident patients. Adjusted by age, sex, family history of cardiovascular disease, family history of diabetes, education, household income, and Townsend deprivation index. * *p*-value less than 0.05 (*p* < 0.05); ** *p*-value less than 0.0018 (*p* < 0.05/28) for Bonferroni correction. The models adjusted for gender, age, education, income, Townsend deprivation index, family history of cardiovascular disease and diabetes, metabolic equivalent of physical activity, alcohol daily consumption, smoking status, and systolic blood pressure. Biochemical indices were further adjusted by cholesterol-lowering medication.

Figure 2 shows the association between incident AVS and modifiable risk factors. Most biological indicators of metabolic factors were statistically significant after adjustment. All three obesity indices (per 1 kg/m^2^ higher BMI (HR [95% confidence interval (CI): 1.06 [1.05–1.07]), per 1% higher BF (1.04 [1.03–1.05]), per 1%-unit WHR (1.03 [1.03–1.04])]) were positively related with incidence of AVS. Both per 10 mmHg higher SBP (1.10 [1.07–1.13]) and PP (1.23 [1.19–1.26]) were significant with an increment of incidence of AVS. Per 1 mmol/L, higher LDL was associated with 1.10 (1.03 to 1.18) higher HR. Per 5 mmol/mol higher increase in HbA1c, which reflects the control of blood glucose levels, was associated with the incidence of AVS (1.07 [1.06, 1.08]). Moreover, 10 μmol/L higher in urate, 1 mg/L higher in CPR, 10 mmol/L higher in creatinine, and 1 g/L lower albumin was associated with 1.02 (1.02 to 1.03), 1.03 (1.02 to 1.04), 1.04 (1.03 to 1.05) and 0.92 (0.90 to 0.94) higher HRs, respectively.

**Figure 3:**
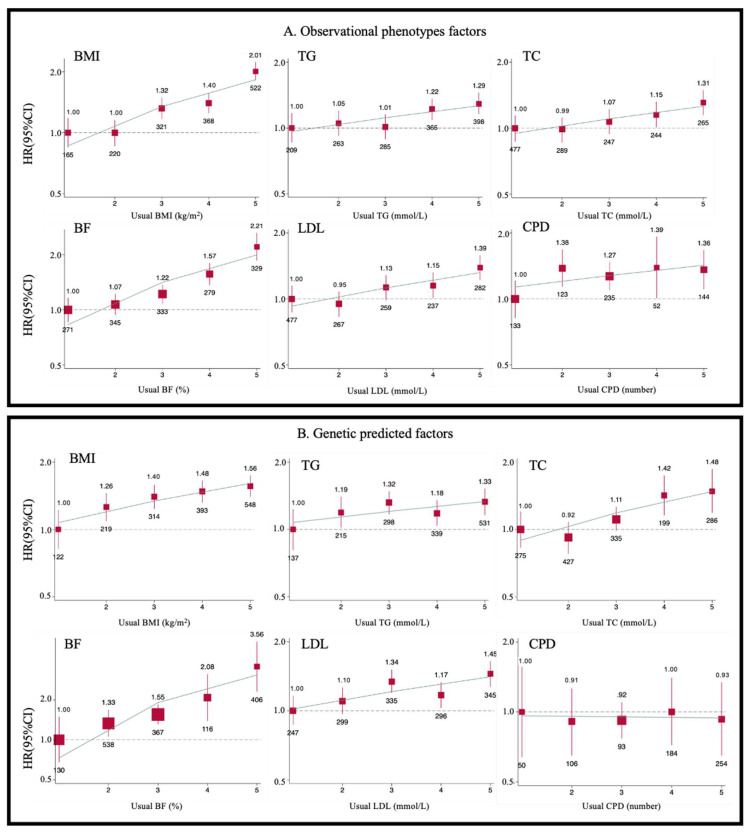
Adjusted HRs for risk of AVS by fifths of BMI, BF, TG, LDL, TC, and CPD in observational analysis and Mendelian randomization analysis. Cox regression was used to estimate the HRs and 95% CIs for AVS (*n* = 1602) by fifths of BMI, BF, TG, LDL, TC, and CPD in observational analysis (**A**) and Mendelian randomization analysis (**B**). Each square has an area inversely proportional to the variance of the log risk in a specific group. The number below each square is the AVS case number of these factors. The line represents the slope from a weighted linear regression with the weights based on the inverse variance of the log HR.

The HR was 0.96 (0.93 to 0.99) of years of educational attainment. We found suggestive associations among three indicators of smoking (per 10 more cigarettes per day: 1.11 [1.04–1.18], smoking initiation: 1.35 [1.21–1.50] compared to no smoking, and smoking cessation: 0.76 [0.65–0.90] compared to current smoking) and AVS. Each additional cup of coffee was associated with a 4% higher risk of incident AVS (1.04 [1.02–1.07]). It turned out that those who napped during the daytime or had insomnia (1.28 [1.15–1.43] compared to those who never nap and 1.19 [1.05 to 1.35] compared to those who had non-insomnia) were more likely to develop AVS. Participants who get up easily can attenuate AVS incidence with HRs: 0.72 (0.62 to 0.83). Multivariable analyses were adjusted and displayed in Supplementary Table S4. Individuals in the top quintile of BMI, BF, TG, LDL, TC, and CPD had a 17% to 170% higher risk of developing AVS (Supplementary Table S6) compared with those in the first quintile. These effects were attenuated by further adjustment for baseline BMI, WHR, and blood pressure. The associations between modifiable risk factors and AVS were also approximately log-linear throughout the BMI, BF, TG, LDL, TC, and CPD (Figure 3A).

### 3.3. Instrumental Variable Analysis

**Figure 4:**
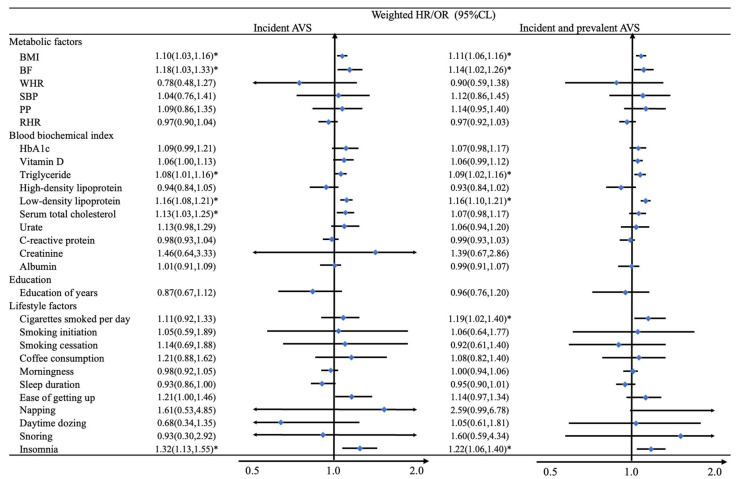
Mendelian randomization analysis between 28 modifiable risk GRS and AVS phenotypes. The MR analysis was adjusted by sex, age, the first 10 genetic principal components, and genotyping chip. The Cox model was used in patients with incident AVS, and the logistic model was used in patients with the incident and prevalent AVS patients. The weighted GRSs used for prediction. * *p*-value less than 0.0018 (after adjusted by Bonferroni correction).

Genetically predicted 1-SD higher levels of BMI [HR:1.10, 95% CI: 1.03 to 1.16], BF (1.18, 1.03 to 1.33), TG [1.08, 1.01 to 1.16], LDL (1.16, 1.08 to 1.21) and TC (1.13, 1.03 to 1.25) were associated with a higher risk of AVS, respectively (Figure 4). We also found that genetically predicted 1-category higher levels of insomnia (1.32, 1.13 to 1.55), which means having a poorer sleep quality, was associated with a higher incidence of AVS. A suggestive association between CPD and AVS was detected in logistic regression with dependent combined with baseline and incidence of AVS. The HRs per genetically predicted 1-SD with a greater number of CPD were 1.19 (1.02 to 1.40). The trends of adjusted HRs for risk of AVS by fifths of predicted BMI, BF, TG, LDL, TC, and CPD can be seen in Figure 3B. Throughout the quintiles range, each group had higher BMI, BF, TG, LDL, TC, and CPD and were associated with 29% to 219% higher AVS risks than the bottom quintile. On the other side, we also found and reported negative results. In terms of biological metabolites, HDL (0.94, 0.84 to 1.05), urate (1.13, 0.98 to 1.29), CRP (0.98, 0.93 to 1.04), creatinine (1.46, 0.64 to 3.33) and Albumin (1.01, 0.91 to 1.09) showed no causal relationship between these metabolic indicators and AVS. Other non-significant results are also shown in Figure 4.

### 3.4. Subgroup Analysis and Sensitivity Analysis

Subgroup analysis was used to check the difference between sex and age group. It illustrated that females were more likely to suffer AVS in higher BF, LDL, many numbers of CPD, and insomnia (Supplementary Table S5). AVS risks varied with age groups, and it seemed that persons among older age groups preferred to develop AVS in different age stratification. Quintiles of original variables and predicted variables in each model were shown in Supplementary Table S6. The sensitivity analysis can be seen in Supplementary Table S7. LDL (1.12, 1.02 to 1.22) and insomnia (1.41, 1.14 to 1.75) remained significant; others were not significant when persons who used lipid-lowering drugs at baseline were excluded. In addition, we found that HbA1c was significant after medical use exclusion. We also excluded the population with the disease onset in the previous three years in the sensitivity analysis. The obtained HRs value was more significant than that before the exclusion. In addition, the methods of MR-Egger and weighted median showed similar results to the present study (Supplementary Table S8).

## 4. Discussion

This is the first study to take advantage of genetic instruments to investigate multiple risk factors associated with AVS risk in a large sample of people of European descent. Our MR analysis suggests that genetically predicted BMI, BF, TG, LDL, and TC had linear associations with AVS. Our findings highlighted the importance of the management of modifiable factors, such as adiposity and lipids, in preventing AVS. However, our genetic results did not discover the causal role of blood pressure, education level, smoking, VD, and coffee intake in developing AVS.

Our observational analysis had supported associations between obesity [46], blood pressure [15,47,48,49], lipids [50,51,52], smoking [53,54], sleep [45,55,56,57], and AVS. Based on the genetic data of the population of up to about 0.3 million, our analysis used the one-sample MR approach to examine the potential causal association between modifiable risk factors and AVS. Our results confirmed previous MR results, showing that gene-predicted indicators of obesity [21], several blood lipids factors [25], and insomnia [23] were associated with AVS. These also support the robustness of our results. Importantly, our MR analysis suggests that genetically predicted BMI, BF, TG, LDL, and TC had linear associations with AVS risk. We also found a potential causal relationship between baseline adjusted smoking and AVS, consistent with previous studies [22]. In addition, we found no evidence of associations between blood pressure, blood glucose, vitamin D, education, coffee intake, and AVS. Although the associations between these factors and AVS were found in the observational analysis of this study, it may be due to reverse causality bias that confused the true relationships.

There are several possible mechanisms for causal associations of obesity and lipids with AVS risk. Firstly, obesity affects the progression of AVS by affecting blood glucose. Hyperglycemia is associated with the effects of blood vessels and inflammatory cells and advanced glycation end products (AGEs) [58]. AGEs can attach to amino groups in free segments of proteins, causing lipid metabolism disorders and excessive production of inflammatory cytokines [59], which play an essential role in vascular calcification [58,59,60,61,62,63]. In addition, obesity may affect the blockage of the aortic valve through blood cholesterol [64,65]. Similar to previous results, this study also found potential causal evidence for some blood lipids [66,67]. It is believed that the possible cause of aortic stenosis is atherosclerosis [68,69,70], and cholesterol is involved in the process of atherosclerosis and is an apparent risk factor for AVS [15,71]. Lipoprotein deposition induces AVS to appear in its symptoms [18,58,59,60,61,62,63]. The formation of reactive oxygen species may be related to the calcification of the valve [72]. This oxidative stress promotes the formation of oxidized phospholipids, which can be converted to lysophosphatidylcholine via lipoprotein phospholipase A2, leading to apoptosis of arterial valve endothelial cells [73,74]. Moreover, a study had shown that about 8% of the indirect effect of obesity on coronary disease is due to cholesterol as a mediator [75]. Lipo-lowering drugs may provide an effective way to prevent AVS. For example, a randomized controlled trial evaluating the effectiveness of a targeted apolipoprotein drug showed that the novel drug effectively lowered serum lipids [58], which could help develop drugs to treat AVS. Our findings imply that reducing excessive body weight and maintaining normal blood lipids through promoting exercise and healthy diet habits are of great public health significance in preventing AVS.

As for lifestyle factors, earlier studies have shown that insufficient sleep is one of the causes of AVS [76]. A previous MR analysis in the UK biobank about sleep duration and CVD demonstrated that short sleep duration is a potential causal risk factor for several CVDs [76]. Poor sleep quality may affect CVD through pathways, including inflammation, nervous system dysfunction, and metabolic abnormalities [77,78,79], indicating that improving sleep quality may prevent CVD development. Although no causal link was found in sleep duration, insomnia is one of the signs of insufficient sleep and disturbance of the biorhythm, suggesting that chronic sleep deprivation may cause AVS. Previous findings have shown that insomnia was one of the causes of AVS [23].

Advantages of this study include a large sample size and the exploration of associations between multiple potential exposures and outcomes. In addition, the data were collected from the general European population with a high genotype quality. Moreover, all genome-wide significant loci (*p* < 5 × 10^−8^) we selected were reported by European descent GWAS with a large sample size, indicating our results’ reliability. The genetic risk score constructed for each modifiable factor had no significant pleiotropic effects on AVS, an important MR analysis assumption. However, the present study has several limitations. Firstly, our sample focused on European ancestry, which may limit its applicability to other ethnicities. Secondly, one-sample MR analysis was susceptible to false significant associations, and the bias of weak instrumental variables is inevitably present in this study. We tested the instrumental variables before MR, and the F-statistic showed that it was valid for predicting modifiable risk factors. Thirdly, there may exist a winner curse in GWAS selection. As one of the largest biological databases, there is inevitable sample overlap between participants in the UKB and selected GWAS. We have tried to select GWAS made up of non-UKB and European people. Lastly, the observational association was under the assumption of linearity, which may cause bias. Future studies may further explore the nonlinear association between modifiable risk factors and AVS.

## 5. Conclusions

Our observational studies and genetic analysis proved that higher body mass index, body fat percentage, triglyceride, low-density lipoprotein, total serum cholesterol, and insomnia were associated with the risk of AVS development, providing an essential basis for future prevention strategies for AVS. However, other modifiable risk factors may not play a causal role in developing AVS.

## Figures and Tables

**Table 1:** Characteristics of the study population.

	UKB with Genotype	Patients with Incident AVS *	Patients with Non-AVS
**No. of participants**	361,930	1602	360,328
**Based demography**			
Age, year	56.6 ± 8.0	62.8 ± 5.3	56.5 ± 8.0
Male, No. (%)	171,409 (47.4)	1048 (65.4)	170,360(47.3)
Female, No. (%)	190,523 (52.6)	554 (34.6)	189,968 (52.7)
Family history of CVD, No. (%)	216,176 (59.7)	1004 (62.7)	215,172 (59.7)
**Metabolic factors**			
Body mass index, kg/m^2^	27.4 ± 4.7	29.3 ± 5.3	27.4 ± 4.7
Body fat percentage, %	31.2 ± 8.5	31.6 ± 8.6	31.2 ± 8.5
Waist-hip ratio	0.87 ± 0.09	0.92 ± 0.09	0.87 ± 0.09
Systolic blood pressure, mmHg	140.1 ± 19.6	149.2 ± 20.8	140.0 ± 19.6
Pulse pressure, mmHg	57.8 ± 14.8	67.5 ± 17.8	57.7 ± 14.8
Resting heart rate, bpm	69.4 ± 11.7	70.6 ± 13.1	69.4 ± 11.7
**Biochemical index**			
HbA1c, mmol/mol	35.9 ± 6.5	39.5 ± 11.5	35.9 ± 6.5
Vitamin D, nmol/L	49.7 ± 20.9	48.6 ± 20.8	49.7 ± 20.9
Triglyceride, mmol/L	1.76 ± 1.03	1.97 ± 1.16	1.76 ± 1.03
High-density lipoprotein, mmol/L	1.45 ± 0.38	1.35 ± 0.37	1.45 ± 0.38
Low-density lipoprotein, mmol/L	3.57 ± 0.87	3.40 ± 0.98	3.57 ± 0.87
Total cholesterol, mmol/L	5.71 ± 1.14	5.45 ± 1.29	5.71 ± 1.14
Urate	310.0 ± 80.3	346.1 ± 86.1	309.8 ± 80.3
C-reactive protein	2.55 ± 4.26	3.69 ± 5.68	2.54 ± 4.25
Creatinine	72.4 ± 17.5	79.7 ± 35.7	72.4 ± 17.4
Albumin	45.3 ± 2.6	44.6 ± 2.62	45.3 ± 2.60
**Education**			
Age completed full-time education, y	16.6 ± 2.2	16.1 ± 2.1	16.6 ± 2.2
**Lifestyle factors**			
Cigarettes per day, number	18.4 ± 10.1	21.1 ± 11.9	18.4 ± 10.1
Smoking initiation	161,951 (44.8)	939 (58.6)	161,010 (44.7)
Smoking cessation	125,524 (77.51)	736 (78.0)	124,791 (77.5)
Coffee consumption, cups/day	2.21 ± 2.14	2.46 ± 2.40	2.21 ± 2.14
Morningness, No. (%)	202,942 (62.8)	916 (64.6)	202,026 (62.8)
Sleep duration, hours/day	7.17 ± 1.08	7.24 ± 1.28	7.17 ± 1.08
Getting up easily, No. (%)	299,356 (82.9)	1337 (83.6)	298,019 (82.9)
Napping, No. (%)	156,378 (43.2)	946 (59.1)	155,432 (43.2)
Daytime dozing, No. (%)	82,613 (22.9)	487 (30.6)	82,126 (22.9)
Snoring, No. (%)	127,531 (37.8)	630 (42.8)	126,901 (37.8)
Insomnia, No. (%)	274,346 (75.9)	1274 (79.7)	273,072 (75.8)

* AVS = Aortic Valve Stenosis.

## Data Availability

Data are available in a public, open access repository. This research has been conducted using the UK Biobank Resource under application number 44430. The UK Biobank data are available on application to the UK Biobank (www.ukbiobank.ac.uk/, accessec on 28 May 2022).

## Supplementary Materials

The following supporting information can be downloaded at: https://www.mdpi.com/article/10.3390/nu14112273/s1, Table S1: List of data of modifiable risk factors using for creating genetic risk scores, including metabolic factors, biochemical index, education, lifestyle factors; Table S2: The genetic variants of modifiable risk factors; Table S3: Information of phenotypic variables; Table S4: Hazard ratio of modifiable risk factors and AVS incident patients; Table S5: Adjusted Hazard Ratios for AVS events by gender and age; Table S6: Adjusted hazard ratios for AVS by quintiles of baseline and predicted variants; Tables S7 and S8: Sensitivity MR analyses.

## Abbreviations

Aortic valve stenosis (AVS); cardiovascular disease (CVDs); genetic risk score (GRSs); hazard ratios (HRs); confidence intervals (CIs); single nucleotide polymorphism (SNPs); body mass index (BMI); body fat percentage (BF); waist-to-hip ratio (WHR); systolic blood pressure (SBP); pulse pressure (PP); vitamin D (VD); cigarettes consumption per day (CPD); triglyceride (TG); high-density lipoprotein (HDL); low-density lipoprotein (LDL); serum total cholesterol (TC); resting heart rate (RHR); glycated hemoglobin (HbA1c); C-reactive protein (CRP); genome-wide association studies (GWASs); Mendelian randomization (MR).
