# Supplementary material for: Observational and Genetic Associations of Modifiable Risk Factors with Aortic Valve Stenosis: A Prospective Cohort Study of 0.5 Million Participants
Source: Nutrients. 2022 May 28;14(11):2273. doi: 10.3390/nu14112273 (PMC9182826; doi:10.3390/nu14112273)
Supplement: Supplementary file 1 [file nutrients-14-02273-s001.zip › nutrients-1698219-Table S2.pdf]

SNPs which can not pass the Hardy-Weinberg test (H-W test) had been omitted in creating GRSs. SNP = single nucleotide polymorphism; Chr = Chromosome; SE = standard error; MR = Mendelian randomization; BMI = body mass index; BF = body fat percentage; WHR = waist-to-hip ratio; SBP = systolic blood pressure; PP = pulse pressure; TG = triglyceride; HDL = high-density lipoprotein; LDL = low-density lipoprotein; TC = serum total cholesterol; RHR = resting heart rate; HbA1c = glycated hemoglobin.

Table S2. Summary statistics for the genetic variants of modifiable risk factors using for creating GRSs with Aortic valve stenosis disease in the Mendelian randomization study.

Trait	SNP	Chr	Position	Effect Alleles	Other Alleles	β or OR	P value
BMI	rs977747	1	47684677	T	G	0.0167	8.652E-08
BMI	rs657452	1	49589847	A	G	0.0227	5.482E-13
BMI	rs11583200	1	50559820	C	T	0.0177	1.479E-08
BMI	rs3101336	1	72751185	C	T	0.0334	2.661E-26
BMI	rs12566985	1	75002193	G	A	0.0242	3.282E-15
BMI	rs12401738	1	78446761	A	G	0.0211	1.145E-10
BMI	rs11165643	1	96924097	T	C	0.0218	2.07E-12
BMI	rs17024393	1	110154688	C	T	0.0658	7.029E-14
BMI	rs543874	1	177889480	G	A	0.0482	2.618E-35
BMI	rs2820292	1	201784287	C	A	0.0195	1.834E-10
BMI	rs13021737	2	632348	G	A	0.0601	1.113E-50
BMI	rs10182181	2	25150296	G	A	0.0307	8.777E-24
BMI	rs11126666	2	26928811	A	G	0.0207	1.332E-09
BMI	rs1016287	2	59305625	T	C	0.0229	2.253E-11
BMI	rs11688816	2	63053048	G	A	0.0172	1.893E-08
BMI	rs2121279	2	143043285	T	C	0.0245	2.313E-08
BMI	rs1460676	2	164567689	C	T	0.0197	8.982E-07
BMI	rs1528435	2	181550962	T	C	0.0178	1.196E-08
BMI	rs17203016	2	208255518	G	A	0.021	8.145E-08
BMI	rs7599312	2	213413231	G	A	0.022	1.173E-10
BMI	rs492400	2	219349752	C	T	0.0158	4.167E-07
BMI	rs2176040	2	227092802	A	G	0.0141	0.000006058
BMI	rs6804842	3	25106437	G	A	0.0185	2.476E-09

BMI	rs2365389	3	61236462	C	T	0.02	1.629E-10
BMI	rs3849570	3	81792112	A	C	0.0188	2.601E-08
BMI	rs13078960	3	85807590	G	T	0.0297	1.737E-14
BMI	rs16851483	3	141275436	T	G	0.0483	3.548E-10
BMI	rs1516725	3	185824004	C	T	0.0451	1.886E-22
BMI	rs10938397	4	45182527	G	A	0.0402	3.205E-38
BMI	rs17001654	4	77129568	G	C	0.0306	7.76E-09
BMI	rs13107325	4	103188709	T	C	0.0477	1.825E-12
BMI	rs11727676	4	145659064	T	C	0.0358	2.55E-08
BMI	rs2112347	5	75015242	T	G	0.0261	6.191E-17
BMI	rs7715256	5	153537893	G	T	0.0163	1.695E-07
BMI	rs205262	6	34563164	G	A	0.0221	1.753E-10
BMI	rs2033529	6	40348653	G	A	0.019	1.388E-08
BMI	rs2207139	6	50845490	G	A	0.0447	4.126E-29
BMI	rs9400239	6	108977663	C	T	0.0188	1.613E-08
BMI	rs9374842	6	120185665	T	C	0.0187	9.673E-08
BMI	rs13201877	6	137675541	G	A	0.0233	2.348E-07
BMI	rs13191362	6	163033350	A	G	0.0277	7.339E-09
BMI	rs1167827	7	75163169	G	A	0.0202	6.333E-10
BMI	rs2245368	7	76608143	C	T	0.0317	3.187E-08
BMI	rs9641123	7	93197732	C	G	0.0191	0.0000005
BMI	rs6465468	7	95169514	T	G	0.0166	0.000002318
BMI	rs17405819	8	76806584	T	C	0.0224	2.07E-11
BMI	rs16907751	8	81375457	C	T	0.035	1.255E-07
BMI	rs2033732	8	85079709	C	T	0.0192	4.889E-08
BMI	rs4740619	9	15634326	T	C	0.0179	4.564E-09
BMI	rs10968576	9	28414339	G	A	0.0249	6.607E-14
BMI	rs6477694	9	111932342	C	T	0.0174	2.673E-08
BMI	rs1928295	9	120378483	T	C	0.0188	7.91E-10
BMI	rs10733682	9	129460914	A	G	0.0174	1.83E-08
BMI	rs7899106	10	87410904	G	A	0.0395	2.96E-08
BMI	rs17094222	10	102395440	C	T	0.0249	5.942E-11
BMI	rs11191560	10	104869038	C	T	0.0308	8.446E-09

BMI	rs7903146	10	114758349	C	T	0.0234	1.112E-11
BMI	rs4256980	11	8673939	G	C	0.0209	2.9E-11
BMI	rs11030104	11	27684517	A	G	0.0414	5.557E-28
BMI	rs2176598	11	43864278	T	C	0.0198	2.971E-08
BMI	rs3817334	11	47650993	T	C	0.0262	5.145E-17
BMI	rs12286929	11	115022404	G	A	0.0217	1.31E-12
BMI	rs7138803	12	50247468	A	G	0.0315	8.153E-24
BMI	rs11057405	12	122781897	G	A	0.0307	2.019E-08
BMI	rs9581854	13	28017782	T	C	0.0298	2.291E-10
BMI	rs12429545	13	54102206	A	G	0.0334	1.094E-12
BMI	rs9540493	13	66205704	A	G	0.0172	1.416E-07
BMI	rs1441264	13	79580919	A	G	0.0175	6.037E-08
BMI	rs10132280	14	25928179	C	A	0.023	1.141E-11
BMI	rs12885454	14	29736838	C	A	0.0207	1.943E-10
BMI	rs11847697	14	30515112	T	C	0.0492	3.99E-09
BMI	rs7141420	14	79899454	T	C	0.0235	1.23E-14
BMI	rs3736485	15	51748610	A	G	0.0176	7.412E-09
BMI	rs16951275	15	68077168	T	C	0.0311	1.911E-17
BMI	rs7164727	15	73093991	T	C	0.018	6.829E-08
BMI	rs758747	16	3627358	T	C	0.0225	7.473E-10
BMI	rs12446632	16	19935389	G	A	0.0403	1.477E-18
BMI	rs2650492	16	28333411	A	G	0.0207	1.915E-09
BMI	rs3888190	16	28889486	A	C	0.0309	3.14E-23
BMI	rs4787491	16	30015337	G	A	0.0159	0.000002241
BMI	rs9925964	16	31129895	A	G	0.0192	8.108E-10
BMI	rs2080454	16	49062590	C	A	0.0168	6.547E-08
BMI	rs1558902	16	53803574	A	T	0.0818	7.51E-153
BMI	rs9914578	17	2005136	G	C	0.0201	8.99E-08
BMI	rs1000940	17	5283252	G	A	0.0192	1.284E-08
BMI	rs12940622	17	78615571	G	A	0.0182	2.494E-09
BMI	rs1808579	18	21104888	C	T	0.0167	4.169E-08
BMI	rs7239883	18	40147671	G	A	0.0164	1.633E-07
BMI	rs7243357	18	56883319	T	G	0.0217	3.857E-08

BMI	rs6567160	18	57829135	C	T	0.0556	3.93E-53
BMI	rs17724992	19	18454825	A	G	0.0194	3.415E-08
BMI	rs29941	19	34309532	G	A	0.0182	2.407E-08
BMI	rs2075650	19	45395619	A	G	0.0258	1.247E-08
BMI	rs2287019	19	46202172	C	T	0.036	4.585E-18
BMI	rs3810291	19	47569003	A	G	0.0283	4.812E-15
BMI	rs6091540	20	51087862	C	T	0.0188	8.024E-08
BMI	rs2836754	21	40291740	C	T	0.0164	4.161E-07
BF	rs543874	1	177889480	G	A	0.032	4.8213E-08
BF	rs2943652	2	227108446	C	T	0.034	5.23096E-12
BF	rs6738627	2	165544450	A	G	0.03	9.86588E-10
BF	rs6755502	2	635721	C	T	0.039	4.016E-11
BF	rs693839	13	80958288	C	T	0.028	1.07176E-08
BF	rs1558902	16	53803574	A	T	0.051	9.91363E-25
BF	rs4788099	16	28855727	G	A	0.027	3.33204E-08
BF	rs9906944	17	47091420	C	T	0.033	1.89896E-08
BF	rs6567160	18	57829135	C	T	0.034	5.23096E-12
BF	rs6857	19	45392254	C	T	0.048	9.86588E-10
BF	rs757318	19	18820308	C	A	0.024	0.00000021
BF	rs3761445	22	38595411	G	A	0.024	0.00000017
WHR	rs10919388	1	170372503	C	A	0.024	3.2E-09
WHR	rs2645294	1	119574587	T	C	0.031	1.7E-19
WHR	rs2820443	1	219753509	T	C	0.035	5.3E-21
WHR	rs714515	1	172352990	G	A	0.027	4.4E-15
WHR	rs905938	1	154991389	T	C	0.025	7.3E-10
WHR	rs10195252	2	165513091	T	C	0.027	5.9E-15
WHR	rs1385167	2	66200648	G	A	0.029	1.9E-09
WHR	rs1569135	2	188115398	A	G	0.021	5.6E-10
WHR	rs10804591	3	129334233	A	C	0.025	6.6E-09
WHR	rs17451107	3	156797609	T	C	0.026	1.1E-12
WHR	rs17819328	3	12489342	G	T	0.021	2.4E-09
WHR	rs2276824	3	52637486	C	G	0.024	3.2E-11
WHR	rs2371767	3	64718258	G	C	0.036	1.6E-20

WHR	rs303084	4	124066948	A	G	0.023	0.000000039
WHR	rs3805389	4	56482750	A	G	0.012	0.0015
WHR	rs9991328	4	89713121	T	C	0.019	0.000000045
WHR	rs1045241	5	118729286	C	T	0.019	0.00000044
WHR	rs6556301	5	176527577	T	G	0.022	0.000000026
WHR	rs7705502	5	173320815	A	G	0.027	4.7E-14
WHR	rs9687846	5	55861894	A	G	0.024	0.000000071
WHR	rs1294410	6	6738752	C	T	0.031	2E-18
WHR	rs1358980	6	43764551	T	C	0.039	3.1E-27
WHR	rs1776897	6	34195011	G	T	0.03	0.000011
WHR	rs1936805	6	127452116	T	C	0.043	3.6E-35
WHR	rs7759742	6	32381736	A	T	0.023	4.4E-11
WHR	rs10245353	7	25858614	A	C	0.035	8.4E-16
WHR	rs1534696	7	26397239	C	A	0.011	0.0013
WHR	rs7801581	7	27223771	T	C	0.027	3.7E-10
WHR	rs12679556	8	72514228	G	T	0.027	2.1E-11
WHR	rs7830933	8	23603324	A	G	0.022	0.000000074
WHR	rs10991437	9	107735920	A	C	0.031	0.00000001
WHR	rs7917772	10	104487443	A	G	0.014	0.000056
WHR	rs11231693	11	63862612	A	G	0.041	0.000000045
WHR	rs10842707	12	26471364	T	C	0.032	4.4E-16
WHR	rs1443512	12	54342684	A	C	0.028	6.9E-13
WHR	rs4765219	12	124440110	C	A	0.028	1.6E-15
WHR	rs1440372	15	67033151	C	T	0.024	1.1E-10
WHR	rs8030605	15	56504598	A	G	0.03	8.8E-09
WHR	rs8042543	15	31708263	C	T	0.026	1.2E-09
WHR	rs2925979	16	81534790	T	C	0.018	0.0000012
WHR	rs4646404	17	17420199	G	A	0.027	1.4E-11
WHR	rs8066985	17	68453345	A	G	0.018	0.00000014
WHR	rs12454712	18	60845884	T	C	0.016	0.0001
WHR	rs12608504	19	18389135	A	G	0.022	8.8E-10
WHR	rs4081724	19	33824946	G	A	0.035	7.4E-12
WHR	rs224333	20	34023962	G	A	0.02	0.000000026

WHR	rs6090583	20	45558831	A	G	0.022	6.2E-11
WHR	rs979012	20	6623374	T	C	0.027	3.3E-14
WHR	rs2294239	22	29449477	A	G	0.025	7.2E-13
SBP	rs17367504	1	11862778	A	G	0.4	0.000000043
SBP	rs2761436	1	207919748	T	C	0.3	0.000000033
SBP	rs1275988	2	26914364	C	T	0.54	1.2E-22
SBP	rs16849225	2	164906820	C	T	0.42	3.5E-11
SBP	rs2178452	3	160370160	G	A	0.34	4.1E-09
SBP	rs13107325	4	103188709	C	T	0.59	0.000000035
SBP	rs16998073	4	81184341	T	A	0.54	8.5E-20
SBP	rs4475250	5	114375552	G	A	0.29	0.000000036
SBP	rs35410524	6	96885405	T	C	0.38	0.000000032
SBP	rs17477177	7	106411858	C	T	0.42	2.3E-10
SBP	rs1004467	10	104594507	A	G	0.7	2.1E-17
SBP	rs633185	11	100593538	C	G	0.37	2E-10
SBP	rs7927515	11	76125330	A	C	0.31	0.00000004
SBP	rs2681492	12	90013089	T	C	0.52	1.7E-13
SBP	rs2759308	15	81016227	A	G	0.31	0.00000003
SBP	rs12596053	16	4946794	C	A	0.34	1.8E-09
SBP	rs17608766	17	45013271	C	T	0.48	0.000000013
SBP	rs12606620	18	42008097	G	T	0.31	0.00000005
SBP	rs10427021	19	7259346	T	G	0.45	0.000000019
SBP	rs8105753	19	31927547	A	C	0.32	9.2E-09
SBP	rs1327235	20	10969030	G	A	0.35	3.5E-11
PP	rs783621	1	42368035	A	G	0.23	1.2E-10
PP	rs147696085	1	51021867	G	A	0.37	7.4E-10
PP	rs60199046	1	59663341	A	G	0.25	0.000000001
PP	rs9729719	1	38298207	G	A	-0.23	0.000000011
PP	rs16849225	2	164906820	C	T	0.37	6.8E-19
PP	rs1250247	2	216299629	C	G	0.27	1.1E-10
PP	rs9815354	3	41912651	G	A	0.43	7.6E-19
PP	rs871606	4	54799245	T	C	0.44	7.4E-15
PP	rs13192976	6	152312415	A	T	-0.35	1.2E-10

PP	rs1322640	6	169586887	T	C	-0.27	8.9E-11
PP	rs13197550	6	169716025	C	A	-0.23	2.1E-11
PP	rs17477177	7	106411858	T	C	-0.56	9.5E-35
PP	rs11977526	7	46008110	G	A	0.38	2.6E-24
PP	rs2107595	7	19049388	G	A	-0.36	4.9E-15
PP	rs2282978	7	92264410	T	C	0.22	4.2E-09
PP	rs2071518	8	120435812	C	T	-0.3	1.7E-13
PP	rs1004467	10	104594507	A	G	0.5	3E-19
PP	rs9337951	10	30317073	G	A	-0.27	1.4E-10
PP	rs34872471	10	114754071	T	C	-0.25	8.6E-10
PP	rs11222084	11	130273230	A	T	-0.38	1.1E-21
PP	rs2289125	11	89224453	A	C	-0.31	1.2E-13
PP	rs7927515	11	76125330	C	A	-0.25	1E-10
PP	rs1261744	11	117218460	T	C	-0.27	1.3E-09
PP	rs2681492	12	90013089	T	C	0.27	6.3E-09
PP	rs76785029	12	94882905	C	T	0.43	0.000000013
PP	rs7977389	12	49981722	T	C	0.31	0.000000048
PP	rs2244643	14	92359022	A	C	-0.25	7.4E-10
PP	rs12050260	14	23761094	T	C	0.21	0.000000048
PP	rs1036477	15	48914926	A	G	0.34	2.1E-10
PP	rs62011052	15	79156983	T	C	-0.29	3.5E-09
PP	rs7500448	16	83045790	A	G	0.25	4.6E-09
PP	rs17608766	17	45013271	T	C	-0.4	1.4E-12
PP	rs2193635	18	43096236	C	T	-0.32	9.9E-13
PP	rs12606620	18	42008097	G	T	0.25	6.5E-11
PP	rs10418305	19	15278808	C	G	-0.37	3.2E-11
RHR	rs11118555	1	207940853	A	T	0.612	3.88E-26
RHR	rs17362588	2	179721046	A	G	0.736	3.57E-26
RHR	rs4140885	2	188333064	A	G	0.217	4.72E-08
RHR	rs13030174	2	232271284	A	C	0.3	1.04E-10
RHR	rs9647379	3	171785168	C	G	0.206	1.17E-09
RHR	rs7612445	3	179172979	G	T	0.358	1.86E-14
RHR	rs6882776	5	172664163	G	A	0.301	2.29E-12

RHR	rs11153730	6	118667522	T	C	0.381	7.55E-21
RHR	rs1015451	6	122131485	C	T	0.713	1.14E-32
RHR	rs180242	7	93549596	T	A	0.316	6.78E-12
RHR	rs13245899	7	100497131	G	A	0.447	7.67E-27
RHR	rs2350782	7	136642634	C	T	0.505	1.26E-12
RHR	rs174549	11	61571382	A	G	0.358	1.38E-22
RHR	rs17287293	12	24770878	A	G	0.444	3.07E-20
RHR	rs7980799	12	33576990	A	C	0.377	6.22E-24
RHR	rs826838	12	39106731	C	T	0.234	3.73E-09
RHR	rs2067615	12	107149422	A	T	0.278	1.58E-09
RHR	rs365990	14	23861811	G	A	0.564	5.39E-45
RHR	rs17796783	14	85809911	T	C	0.334	2.69E-13
RHR	rs4489968	15	73665506	T	G	0.513	3.82E-20
RHR	rs6127471	20	36844038	C	T	0.429	5.22E-29
Fasting glucose	rs340874	1	212225879	C	T	0.013464	4.08E-10
Fasting glucose	rs780094	2	27594741	C	T	0.027386	2.58E-37
Fasting glucose	rs560887	2	169471394	C	T	0.071132	0
Fasting glucose	rs7651090	3	186996086	G	A	0.0128	1.75E−8
Fasting glucose	rs11715915	3	49430334	C	T	0.012	4.9E−8
Fasting glucose	rs11708067	3	124548468	A	G	0.023016	1.3E-18
Fasting glucose	rs1280	3	172195984	T	C	0.026374	8.56E-18
Fasting glucose	rs4869272	5	95565204	T	C	0.0177	1.0E−15
Fasting glucose	rs9368222	6	20794975	A	C	0.0143	1.0E−9
Fasting glucose	rs6943153	7	50759073	T	C	0.0154	1.6E−12
Fasting glucose	rs2191349	7	15030834	T	G	0.029157	0
Fasting glucose	rs2908289	7	44190467	A	G	0.057397	0
Fasting glucose	rs983309	8	9215142	T	G	0.0256	6.3E−15
Fasting glucose	rs11558471	8	118254914	A	G	0.028879	7.8E-37
Fasting glucose	rs10811661	9	22124094	T	C	0.0238	5.6E−18
Fasting glucose	rs16913693	9	110720180	T	G	0.0434	3.5E−11
Fasting glucose	rs3829109	9	138376587	G	A	0.0172	1.1E−10
Fasting glucose	rs10814916	9	4283150	C	A	0.015819	2.26E-13
Fasting glucose	rs11195502	10	113029657	C	T	0.032448	1.97E-18

Fasting glucose	rs7903146	10	114748339	T	C	0.021965	2.71E-20
Fasting glucose	rs11603334	11	72110633	G	A	0.0192	1.1E−11
Fasting glucose	rs11607883	11	45796285	G	A	0.021346	6.32E-24
Fasting glucose	rs11039182	11	47303299	T	C	0.023313	4.82E-22
Fasting glucose	rs174576	11	61360086	C	A	0.019743	0
Fasting glucose	rs10830963	11	92348358	G	C	0.077887	0
Fasting glucose	rs10747083	12	131551691	A	G	0.0133	7.6E−9
Fasting glucose	rs11619319	13	27385599	G	A	0.0195	1.3E−15
Fasting glucose	rs576674	13	32452302	G	A	0.0167	2.3E−8
Fasting glucose	rs3783347	14	99909014	G	T	0.0168	1.3E−10
Fasting glucose	rs4502156	15	60170447	T	C	0.022435	1.38E-25
Fasting glucose	rs2302593	19	50888474	C	G	0.0144	9.3E−10
Fasting glucose	rs6113722	20	22505099	G	A	0.0353	2.5E−11
Fasting glucose	rs6072275	20	39177319	A	G	0.0159	1.7E−8
HbA1c	rs2779116	1	158585415	T	C	0.024	2.75E-09
HbA1c	rs552976	2	169791438	G	A	0.047	8.16E-18
HbA1c	rs1800562	6	26093141	G	A	0.063	2.59E-20
HbA1c	rs1799884	7	44229068	T	C	0.038	1.45E-20
HbA1c	rs4737009	8	41630405	A	G	0.027	6.11E-12
HbA1c	rs16926246	10	71093392	C	T	0.089	3.11E-54
HbA1c	rs1387153	11	92673828	T	C	0.028	3.96E-11
HbA1c	rs7998202	13	113331868	G	A	0.031	5.24E-09
HbA1c	rs1046896	17	80685533	T	C	0.035	1.57E-26
HbA1c	rs855791	22	37462936	A	G	0.027	2.74E-14
Vitamin D	rs12785878	11	71456403	T	G	8.45	
Vitamin D	rs10741657	11	14893332	A	G	3.12	3.27E-20
Vitamin D	rs2282679	4	71742666	T	G	3.7	1.9E-109
Vitamin D	rs6013897	20	54125940	T	A	1.85	6E-10
Triglyceride	rs2131925	1	63025942	T	G	0.066	3E-74
Triglyceride	rs1260326	2	27730940	T	C	0.115	2E-239
Triglyceride	rs645040	3	135926622	T	G	0.029	2E-12
Triglyceride	rs442177	4	88030261	T	G	0.031	1E-18
Triglyceride	rs6831256	4	3473139	G	A	0.026	2E-12

Triglyceride	rs9686661	5	55861786	T	C	0.038	3E-16
Triglyceride	rs998584	6	43757896	A	C	0.029	3E-15
Triglyceride	rs17145738	7	72982874	C	T	0.115	9E-99
Triglyceride	rs13238203	7	72129667	C	T	0.059	0.000003
Triglyceride	rs38855	7	116358044	A	G	0.019	0.00000002
Triglyceride	rs12678919	8	19844222	A	G	0.17	2E-199
Triglyceride	rs2954029	8	126490972	A	T	0.076	1E-107
Triglyceride	rs11776767	8	10683929	C	G	0.022	3E-11
Triglyceride	rs1495741	8	18272881	G	A	0.04	3E-12
Triglyceride	rs1832007	10	5254847	A	G	0.033	2E-12
Triglyceride	rs10761731	10	65027610	A	T	0.031	8E-12
Triglyceride	rs2068888	10	94839642	G	A	0.024	2E-11
Triglyceride	rs964184	11	116648917	G	C	0.234	7E-224
Triglyceride	rs174546	11	61569830	T	C	0.045	7E-38
Triglyceride	rs11613352	12	57792580	C	T	0.028	9E-14
Triglyceride	rs2929282	15	44245931	T	A	0.072	0.000000002
Triglyceride	rs2412710	15	42683787	A	G	0.099	2E-11
Triglyceride	rs11649653	16	30918487	C	G	0.027	0.0000002
Triglyceride	rs3198697	16	15129940	C	T	0.02	0.00000002
Triglyceride	rs8077889	17	41878166	C	A	0.025	0.00000001
Triglyceride	rs7248104	19	7224431	G	A	0.022	5E-10
Triglyceride	rs731839	19	33899065	G	A	0.022	0.000000003
Triglyceride	rs5756931	22	38546033	T	C	0.02	0.00000003
High-density lipoprotein	rs12748152	1	27138393	C	T	0.051	1E-15
High-density lipoprotein	rs4846914	1	230295691	A	G	0.048	4E-41
High-density lipoprotein	rs4660293	1	40028180	A	G	0.035	3E-18
High-density lipoprotein	rs1689800	1	182168885	A	G	0.034	5E-20
High-density lipoprotein	rs12145743	1	156700651	G	T	0.02	0.00000002

High-density lipoprotein	rs4650994	1	178515312	G	A	0.021	0.000000007
High-density lipoprotein	rs1047891	2	211540507	C	A	0.027	9E-10
High-density lipoprotein	rs2972146	2	227100698	G	T	0.032	2E-17
High-density lipoprotein	rs12328675	2	165540800	C	T	0.045	2E-15
High-density lipoprotein	rs2290547	3	47061183	G	A	0.03	0.000000004
High-density lipoprotein	rs6805251	3	119560606	T	C	0.02	0.00000001
High-density lipoprotein	rs2013208	3	50129399	T	C	0.025	9E-12
High-density lipoprotein	rs2606736	3	11400249	C	T	0.025	0.00000005
High-density lipoprotein	rs13326165	3	52532118	A	G	0.029	9E-11
High-density lipoprotein	rs13107325	4	103188709	C	T	0.071	1E-15
High-density lipoprotein	rs10019888	4	26062990	A	G	0.027	0.00000005
High-density lipoprotein	rs3822072	4	89741269	G	A	0.025	4E-12
High-density lipoprotein	rs2602836	4	100014805	A	G	0.019	0.00000005
High-density lipoprotein	rs6450176	5	53298025	G	A	0.025	7E-10
High-density lipoprotein	rs605066	6	139829666	T	C	0.028	0.00000003
High-density lipoprotein	rs1936800	6	127436064	C	T	0.02	3E-10
High-density lipoprotein	rs17173637	7	150529449	T	C	0.036	0.00000002
High-density lipoprotein	rs4142995	7	17919258	G	T	0.026	9E-12

High-density lipoprotein	rs4917014	7	50305863	G	T	0.022	0.00000001
High-density lipoprotein	rs702485	7	6449272	G	A	0.024	6E-12
High-density lipoprotein	rs4731702	7	130433384	T	C	0.029	5E-17
High-density lipoprotein	rs9987289	8	9183358	G	A	0.082	2E-41
High-density lipoprotein	rs2293889	8	116599199	G	T	0.031	4E-17
High-density lipoprotein	rs1883025	9	107664301	C	T	0.07	2E-65
High-density lipoprotein	rs581080	9	15305378	C	G	0.042	1E-19
High-density lipoprotein	rs970548	10	46013277	C	A	0.026	2E-10
High-density lipoprotein	rs2923084	11	10388782	A	G	0.026	0.00000005
High-density lipoprotein	rs499974	11	75455021	C	A	0.026	0.00000001
High-density lipoprotein	rs12801636	11	65391317	A	G	0.024	0.00000003
High-density lipoprotein	rs11246602	11	51512090	C	T	0.034	2E-10
High-density lipoprotein	rs3136441	11	46743247	C	T	0.054	7E-29
High-density lipoprotein	rs7134594	12	110000193	T	C	0.035	2E-13
High-density lipoprotein	rs7134375	12	20473758	A	C	0.021	0.00000001
High-density lipoprotein	rs4765127	12	124460167	T	G	0.032	8E-10
High-density lipoprotein	rs838880	12	125261593	C	T	0.048	6E-32
High-density lipoprotein	rs4759375	12	123796238	T	C	0.056	0.00000003

High-density lipoprotein	rs4983559	14	105277209	G	A	0.02	0.00000001
High-density lipoprotein	rs2652834	15	63396867	G	A	0.028	4E-11
High-density lipoprotein	rs1532085	15	58683366	A	G	0.107	1E-188
High-density lipoprotein	rs2925979	16	81534790	C	T	0.035	1E-19
High-density lipoprotein	rs1121980	16	53809247	G	A	0.02	0.000000007
High-density lipoprotein	rs16942887	16	67928042	A	G	0.083	8E-54
High-density lipoprotein	rs3764261	16	56993324	A	C	0.241	1E-769
High-density lipoprotein	rs11869286	17	37813856	C	G	0.032	3E-17
High-density lipoprotein	rs4148008	17	66875294	C	G	0.028	1E-12
High-density lipoprotein	rs4129767	17	76403984	A	G	0.024	2E-11
High-density lipoprotein	rs7241918	18	47160953	T	G	0.09	1E-44
High-density lipoprotein	rs12967135	18	57849023	G	A	0.026	0.00000004
High-density lipoprotein	rs737337	19	11347493	T	C	0.056	5E-17
High-density lipoprotein	rs7255436	19	8433196	A	C	0.032	0.00000002
High-density lipoprotein	rs17695224	19	52324216	G	A	0.029	2E-13
High-density lipoprotein	rs386000	19	54792761	C	G	0.048	3E-23
High-density lipoprotein	rs1800961	20	43042364	C	T	0.127	2E-34
High-density lipoprotein	rs6065906	20	44554015	T	C	0.059	5E-40

High-density lipoprotein	rs181362	22	21932068	C	T	0.038	4E-18
Low-density lipoprotein	rs2479409	1	55504650	G	A	0.064	3E-50
Low-density lipoprotein	rs267733	1	150958836	A	G	0.033	0.000000005
Low-density lipoprotein	rs629301	1	109818306	T	G	0.167	5E-241
Low-density lipoprotein	rs10490626	2	118835841	G	A	0.051	2E-12
Low-density lipoprotein	rs1250229	2	216304384	C	T	0.024	0.00000003
Low-density lipoprotein	rs1367117	2	21263900	A	G	0.119	1E-182
Low-density lipoprotein	rs2030746	2	121309488	T	C	0.021	0.000000009
Low-density lipoprotein	rs2710642	2	63149557	A	G	0.024	0.000000006
Low-density lipoprotein	rs4299376	2	44072576	G	T	0.081	4E-72
Low-density lipoprotein	rs17404153	3	132163200	G	T	0.034	0.000000002
Low-density lipoprotein	rs7640978	3	32533010	C	T	0.039	0.00000001
Low-density lipoprotein	rs4530754	5	122855416	A	G	0.028	4E-12
Low-density lipoprotein	rs1564348	6	160578860	C	T	0.048	3E-21
Low-density lipoprotein	rs1800562	6	26093141	G	A	0.062	8E-14
Low-density lipoprotein	rs3757354	6	16127407	C	T	0.038	2E-17
Low-density lipoprotein	rs4722551	7	25991826	C	T	0.039	4E-14
Low-density lipoprotein	rs10102164	8	55421614	A	G	0.032	4E-11
Low-density lipoprotein	rs11136341	8	145043543	G	A	0.045	7E-12
Low-density lipoprotein	rs11220462	11	126243952	A	G	0.059	7E-21
Low-density lipoprotein	rs4942486	13	32953388	T	C	0.024	2E-11
Low-density lipoprotein	rs8017377	14	24883887	A	G	0.03	3E-15
Low-density lipoprotein	rs1801689	17	64210580	C	A	0.103	1E-11
Low-density lipoprotein	rs7206971	17	45425115	A	G	0.029	0.0000003
Low-density lipoprotein	rs4420638	19	45422946	G	A	0.225	2E-178
Low-density lipoprotein	rs6511720	19	11202306	G	T	0.221	4E-262
Low-density lipoprotein	rs2328223	20	17845921	C	A	0.03	0.000000006
Low-density lipoprotein	rs364585	20	12962718	G	A	0.025	4E-10
Low-density lipoprotein	rs6029526	20	39672618	A	T	0.044	5E-18
Low-density lipoprotein	rs5763662	22	30378703	T	C	0.077	0.00000001
Serum total cholesterol	rs1077514	1	23766233	T	C	0.03	0.000000006
Serum total cholesterol	rs12027135	1	25775733	T	A	0.027	5E-12

Serum total cholesterol	rs7515577	1	93009438	A	C	0.037	0.00000002
Serum total cholesterol	rs2642442	1	220973563	T	C	0.035	3E-11
Serum total cholesterol	rs514230	1	234858597	T	A	0.039	5E-14
Serum total cholesterol	rs7570971	2	135837906	A	C	0.03	1E-13
Serum total cholesterol	rs2287623	2	169830155	G	A	0.027	4E-12
Serum total cholesterol	rs11694172	2	203532304	G	A	0.028	0.000000002
Serum total cholesterol	rs11563251	2	234679384	T	C	0.037	0.000000001
Serum total cholesterol	rs2290159	3	12628920	G	C	0.037	0.000000002
Serum total cholesterol	rs13315871	3	58381287	G	A	0.036	0.00000004
Serum total cholesterol	rs12916	5	74656539	C	T	0.068	5E-74
Serum total cholesterol	rs6882076	5	156390297	C	T	0.051	5E-41
Serum total cholesterol	rs3177928	6	32412435	A	G	0.048	1E-21
Serum total cholesterol	rs2814982	6	34546560	C	T	0.044	4E-15
Serum total cholesterol	rs2758886	6	39250837	A	G	0.023	0.00000003
Serum total cholesterol	rs9488822	6	116312893	T	A	0.034	0.000000001
Serum total cholesterol	rs9376090	6	135411228	C	T	0.025	0.000000003
Serum total cholesterol	rs1997243	7	1083777	G	A	0.033	3E-10
Serum total cholesterol	rs12670798	7	21607352	C	T	0.036	1E-16
Serum total cholesterol	rs2072183	7	44579180	C	G	0.036	4E-15
Serum total cholesterol	rs2081687	8	59388565	T	C	0.038	9E-12
Serum total cholesterol	rs3780181	9	2640759	A	G	0.044	7E-10
Serum total cholesterol	rs10904908	10	17260290	G	A	0.025	3E-11
Serum total cholesterol	rs2255141	10	113933886	A	G	0.031	7E-16
Serum total cholesterol	rs10128711	11	18632984	C	T	0.031	1E-11
Serum total cholesterol	rs11603023	11	118486067	T	C	0.022	0.00000001
Serum total cholesterol	rs7941030	11	122522375	C	T	0.028	2E-14
Serum total cholesterol	rs4883201	12	9082581	A	G	0.035	0.000000002
Serum total cholesterol	rs11065987	12	112072424	A	G	0.031	2E-16
Serum total cholesterol	rs1169288	12	121416650	C	A	0.032	4E-17
Serum total cholesterol	rs2000999	16	72108093	A	G	0.062	7E-41
Serum total cholesterol	rs314253	17	7091650	T	C	0.023	3E-10
Serum total cholesterol	rs10401969	19	19407718	T	C	0.137	4E-77
Serum total cholesterol	rs492602	19	49206417	G	A	0.031	1E-16

Serum total cholesterol	rs2277862	20	34152782	C	T	0.035	5E-11
Serum total cholesterol	rs2902940	20	39091487	A	G	0.024	9E-10
Serum total cholesterol	rs138777	22	35711098	A	G	0.021	0.00000005
Serum total cholesterol	rs4253772	22	46627603	T	C	0.032	0.00000001
Urate	rs10009618	4	89094008	T	C	-0.089	1.98472E-51
Urate	rs10498730	6	25812069	G	A	-0.084	1.3002E-10
Urate	rs10516194	4	9916209	C	T	-0.47	1.24882E-73
Urate	rs10516207	4	10650693	T	C	-0.065	4.07305E-09
Urate	rs10516796	4	88856733	T	C	-0.033	2.35028E-08
Urate	rs10821905	10	52646093	A	G	0.053	3.44826E-12
Urate	rs11264341	1	155151493	T	C	-0.048	1.03896E-14
Urate	rs11599171	10	61394137	A	G	0.052	1.51705E-13
Urate	rs1165151	6	25821616	G	T	0.092	4.51856E-60
Urate	rs1171614	10	61469538	C	T	0.074	6.47739E-23
Urate	rs11728055	4	10296298	C	A	-0.31	3.5237E-168
Urate	rs1178977	7	72857049	G	A	-0.05	6.6819E-12
Urate	rs11932627	4	10156177	G	T	0.1	5.58856E-11
Urate	rs12418845	11	63873673	A	G	-0.072	3.0856E-08
Urate	rs12512447	4	9586313	A	G	0.092	2.32006E-43
Urate	rs1260326	2	27730940	C	T	-0.077	1.30858E-40
Urate	rs12644047	4	9893403	A	G	-0.16	2.6424E-102
Urate	rs12645163	4	9735520	G	A	-0.12	1.58891E-11
Urate	rs13104360	4	9684804	A	C	0.13	2.41379E-17
Urate	rs1359231	6	25809798	T	G	0.073	1.51565E-17
Urate	rs1394125	15	76158983	A	G	0.043	9.77913E-11
Urate	rs1471633	1	145723739	C	A	-0.061	1.39959E-26
Urate	rs1545207	4	89239492	A	G	0.04	3.74516E-08
Urate	rs1558201	4	10557918	C	G	0.062	8.0891E-26
Urate	rs17013544	4	88823643	A	C	0.046	1.15279E-10
Urate	rs17013995	4	89227094	G	A	0.068	1.46899E-09
Urate	rs17014018	4	89248035	A	G	0.11	9.6917E-14
Urate	rs17050272	2	121306440	A	G	0.037	9.36009E-09
Urate	rs17252870	6	25494666	T	G	-0.049	4.4157E-11

Urate	rs17632159	5	72431482	C	G	-0.038	1.99931E-09
Urate	rs1980449	6	25629658	C	T	-0.038	5.8684E-10
Urate	rs198826	6	26121153	T	C	-0.037	1.94022E-10
Urate	rs2012249	4	10521158	T	C	0.21	2.02768E-27
Urate	rs2018643	4	9947121	C	T	-0.19	1E-200
Urate	rs2078267	11	64334114	T	C	-0.078	8.7277E-36
Urate	rs2079742	17	59465697	C	T	-0.051	6.23735E-09
Urate	rs2108879	4	10538144	G	A	0.11	3.69658E-16
Urate	rs2231142	4	89052323	T	G	0.22	4.4361E-116
Urate	rs2307394	2	148716428	C	T	0.035	7.26457E-09
Urate	rs2384629	2	27606098	G	A	-0.093	3.74438E-09
Urate	rs2671613	10	61486286	C	T	0.038	1.00986E-10
Urate	rs2853749	4	88897814	T	C	-0.05	1.21143E-15
Urate	rs2941484	8	76478768	T	C	0.049	3.90751E-17
Urate	rs3741414	12	57844049	T	C	-0.071	9.7949E-22
Urate	rs4401177	2	28344285	A	G	0.058	2.76847E-09
Urate	rs4693935	4	89139275	G	A	0.053	1.08343E-15
Urate	rs4697936	4	10149595	G	T	0.1	1.69278E-49
Urate	rs559566	11	64581658	A	G	-0.058	2.70657E-09
Urate	rs575416	5	72437534	G	A	0.055	3.63212E-09
Urate	rs642803	11	65560620	T	C	-0.043	4.5144E-14
Urate	rs653178	12	112007756	T	C	-0.036	2.44748E-10
Urate	rs6598541	15	99271135	G	A	-0.044	5.19996E-13
Urate	rs675209	6	7102084	C	T	-0.063	1.37594E-21
Urate	rs6770152	3	53100214	T	G	-0.048	2.66318E-16
Urate	rs6811287	4	10180823	T	C	-0.2	1E-200
Urate	rs6830367	4	10671323	C	G	0.051	2.35126E-11
Urate	rs6833095	4	10506599	A	G	0.071	8.03896E-35
Urate	rs6854361	4	88776864	A	C	0.21	5.55776E-17
Urate	rs7193778	16	69563890	T	C	-0.047	2.35923E-08
Urate	rs7224610	17	53364788	A	C	-0.038	4.73915E-11
Urate	rs727996	4	10314188	T	G	0.16	2.74663E-12
Urate	rs729761	6	43804571	G	T	0.046	3.05351E-12

Urate	rs7436833	4	10311074	C	T	0.14	2.4632E-32
Urate	rs753763	10	61445412	C	T	-0.11	3.81022E-08
Urate	rs7654258	4	10404512	T	C	-0.099	1.09295E-19
Urate	rs7669296	4	9942642	C	G	-0.16	9.20725E-09
Urate	rs7938871	11	64223527	A	C	0.033	2.06524E-08
Urate	rs882210	1	145732946	C	G	0.15	1.71874E-08
Urate	rs893006	11	64365796	A	C	-0.063	1.05196E-24
Urate	rs9291640	4	10007086	T	C	0.31	1E-200
Urate	rs9467606	6	25809218	G	A	0.074	1.8395E-13
C-reactive protein	rs10240168	7	22819334	G	C	-0.028684	4.1096E-11
C-reactive protein	rs10437340	1	159710809	C	G	0.110844	7.8163E-197
C-reactive protein	rs10512597	17	72699833	C	T	0.036931	4.44017E-14
C-reactive protein	rs1051338	10	91007360	G	T	0.023881	2.27002E-09
C-reactive protein	rs10521222	16	51158710	T	C	-0.104411	2.06016E-22
C-reactive protein	rs10754557	1	247599232	A	G	-0.037174	2.5398E-14
C-reactive protein	rs10774580	12	121476423	G	A	-0.059655	1.42004E-55
C-reactive protein	rs10832027	11	13357183	A	G	0.025944	4.42996E-12
C-reactive protein	rs10925027	1	247612562	C	T	-0.036035	4.25011E-21
C-reactive protein	rs11065176	12	121064364	T	C	-0.066903	2.11009E-11
C-reactive protein	rs11065270	12	121240862	C	T	0.038276	2.20999E-08
C-reactive protein	rs11065360	12	121386532	G	A	0.060782	2.59E-61
C-reactive protein	rs11065363	12	121388498	T	C	0.082866	5.50047E-61
C-reactive protein	rs11065387	12	121427653	C	G	-0.150349	3.31971E-25
C-reactive protein	rs11065390	12	121439598	A	G	0.076425	4.92947E-12
C-reactive protein	rs11065445	12	121568945	G	C	-0.095071	1.69981E-11
C-reactive protein	rs11108056	12	95855385	G	C	-0.027907	5.42001E-14
C-reactive protein	rs11249928	8	9363260	C	T	0.034719	6.10942E-20
C-reactive protein	rs11265608	1	154364140	A	G	0.043053	4.48023E-13
C-reactive protein	rs1146430	1	91395267	T	C	-0.033857	2.61E-09
C-reactive protein	rs11577275	1	66497062	A	G	0.033549	6.06038E-18
C-reactive protein	rs11615578	12	121714935	T	C	0.047139	9.79941E-27
C-reactive protein	rs11673139	19	45383037	T	A	0.05651	3.83972E-16
C-reactive protein	rs11690539	2	113658015	A	C	-0.021656	8.40001E-09

C-reactive protein	rs1169282	12	121420430	T	C	-0.1673	1.32008E-36
C-reactive protein	rs11783705	8	10063879	A	C	-0.032575	2.84971E-14
C-reactive protein	rs12026296	1	159608991	C	T	-0.095193	1.55991E-28
C-reactive protein	rs12043437	1	159398715	C	T	-0.04123	2.91005E-12
C-reactive protein	rs12117074	1	159538506	A	G	0.104477	2.71019E-18
C-reactive protein	rs12202641	6	116314634	T	C	-0.022804	2.99999E-10
C-reactive protein	rs12565738	1	247609328	T	C	-0.038775	2.36974E-11
C-reactive protein	rs12587622	14	73365174	A	G	-0.020798	8.52001E-09
C-reactive protein	rs1260326	2	27730940	C	T	-0.073462	2.72019E-92
C-reactive protein	rs12748387	1	159269795	C	G	0.123772	1.46016E-19
C-reactive protein	rs12960928	18	57897803	C	T	0.024	1.90999E-09
C-reactive protein	rs12995480	2	629881	C	T	0.031261	1.24E-10
C-reactive protein	rs13233571	7	72971231	T	C	-0.056895	2.94985E-25
C-reactive protein	rs13248595	8	8965993	G	A	-0.026653	6.21012E-11
C-reactive protein	rs1327121	1	65957337	A	G	0.063956	4.54046E-65
C-reactive protein	rs13409371	2	113838145	A	G	0.048232	5.06991E-36
C-reactive protein	rs1371614	2	27152874	T	C	-0.026321	5.22998E-10
C-reactive protein	rs1386821	1	154382049	G	T	-0.05257	4.74024E-29
C-reactive protein	rs1441169	2	214033530	G	A	-0.024926	2.26986E-11
C-reactive protein	rs1446975	1	159629342	C	T	0.072188	7.81088E-84
C-reactive protein	rs1490384	6	126851160	T	C	-0.024816	2.64972E-12
C-reactive protein	rs1509394	2	28647084	T	C	0.025685	6.05006E-10
C-reactive protein	rs1558902	16	53803574	A	T	0.033926	5.19996E-20
C-reactive protein	rs1566043	16	51113337	A	G	-0.033558	5.10975E-12
C-reactive protein	rs1582763	11	60021948	A	G	-0.022107	2.37001E-09
C-reactive protein	rs1616534	8	11666451	T	C	0.030177	3.87972E-16
C-reactive protein	rs1657794	15	60953851	A	G	0.024325	2.55E-10
C-reactive protein	rs17097193	1	66067396	C	T	0.068539	2.78997E-09
C-reactive protein	rs17459069	1	159648693	T	C	-0.212723	1.66994E-54
C-reactive protein	rs17616063	16	51436882	G	A	-0.106356	1.80011E-21
C-reactive protein	rs17624213	2	113933247	T	G	0.030046	7.15007E-09
C-reactive protein	rs17643262	19	45631816	A	G	-0.033345	1.41E-08
C-reactive protein	rs17658229	5	172191052	C	T	0.055568	5.49997E-09

C-reactive protein	rs17706100	2	27876666	G	T	0.05941	5.47999E-10
C-reactive protein	rs17759740	2	28393115	A	T	-0.033895	1.16001E-08
C-reactive protein	rs178810	17	16097430	T	C	0.02001	2.94999E-08
C-reactive protein	rs1800796	7	22766246	C	G	-0.059421	8.71967E-13
C-reactive protein	rs1800961	20	43042364	T	C	-0.1115	4.6302E-23
C-reactive protein	rs1805096	1	66102257	A	G	-0.104381	2.1677E-183
C-reactive protein	rs1880241	7	22759469	G	A	-0.027537	8.41008E-14
C-reactive protein	rs1965024	16	51173559	A	G	-0.039032	1.40994E-21
C-reactive protein	rs1985096	19	45346551	T	A	-0.042135	7.47997E-11
C-reactive protein	rs2064009	8	117007850	T	C	0.027111	2.27982E-14
C-reactive protein	rs2121477	1	159568260	T	C	-0.064985	4.84005E-08
C-reactive protein	rs2141371	2	27860258	A	G	0.045892	3.12968E-25
C-reactive protein	rs2178464	12	121441440	C	T	-0.096193	2.41002E-10
C-reactive protein	rs2239222	14	73011885	G	A	0.035484	9.86961E-20
C-reactive protein	rs2293476	1	40036847	C	G	0.030262	8.2699E-13
C-reactive protein	rs2315008	20	62343956	G	T	0.023467	5.36006E-10
C-reactive protein	rs2352975	3	49891885	C	T	0.024897	6.42999E-10
C-reactive protein	rs2393795	12	121378877	A	G	-0.068502	1.22999E-15
C-reactive protein	rs2503166	1	66619578	A	G	0.027515	3.63999E-13
C-reactive protein	rs2522137	12	120779931	G	T	0.022087	6.36004E-10
C-reactive protein	rs2523488	6	31350855	C	T	0.029454	1.22999E-09
C-reactive protein	rs2647972	16	51668195	C	T	0.020801	7.03008E-09
C-reactive protein	rs2686344	12	121690548	C	T	0.043182	2.01002E-19
C-reactive protein	rs2686555	12	121095172	G	A	0.057313	1.36994E-53
C-reactive protein	rs2708101	12	121487632	T	C	-0.050726	5.73984E-39
C-reactive protein	rs2710804	7	36084529	C	T	0.021262	1.29999E-08
C-reactive protein	rs2794520	1	159678816	T	C	-0.182186	1.2331E-305
C-reactive protein	rs2798604	1	154798153	T	G	0.021868	1.24E-08
C-reactive protein	rs2808658	1	159613798	T	C	0.030833	3.3597E-11
C-reactive protein	rs2836878	21	40465534	A	G	-0.042902	7.71081E-26
C-reactive protein	rs2852151	18	12841176	A	G	0.024735	1.35988E-11
C-reactive protein	rs2891677	8	126344208	T	C	0.019859	1.59001E-08
C-reactive protein	rs2927438	19	45242107	G	A	0.034816	4.86968E-12

C-reactive protein	rs3093070	1	159680817	G	T	0.220217	2.02023E-30
C-reactive protein	rs3093075	1	159679913	T	G	0.225505	2.0184E-204
C-reactive protein	rs3134996	6	32636866	T	A	0.023199	2.81002E-08
C-reactive protein	rs3136447	11	46744368	C	T	-0.028507	2.04998E-08
C-reactive protein	rs330078	8	9155685	T	C	-0.025589	4.70999E-09
C-reactive protein	rs340005	15	60878030	A	G	0.030007	1.00995E-15
C-reactive protein	rs3763327	6	32413830	C	G	0.024658	3.26964E-11
C-reactive protein	rs3766922	1	154577055	G	T	-0.030948	1.3499E-18
C-reactive protein	rs3790429	1	66036776	A	T	-0.049141	2.07014E-17
C-reactive protein	rs3794205	12	121686929	A	G	0.035182	5.45004E-17
C-reactive protein	rs3845622	1	159171603	A	C	-0.064374	9.91973E-18
C-reactive protein	rs405697	19	45404691	G	A	-0.05727	1.10002E-25
C-reactive protein	rs4092465	18	55080437	G	A	0.027483	3.11E-10
C-reactive protein	rs4129267	1	154426264	T	C	-0.087519	1.1995E-129
C-reactive protein	rs4130236	1	159341851	T	C	0.028132	4.46992E-13
C-reactive protein	rs4246598	2	88438050	A	C	0.022063	5.10999E-10
C-reactive protein	rs4420638	19	45422946	G	A	-0.229459	2.2594E-307
C-reactive protein	rs4474240	1	154457855	C	A	-0.048289	8.23E-25
C-reactive protein	rs4665955	2	27383276	A	C	-0.021673	5.48997E-09
C-reactive protein	rs469772	1	91530305	T	C	-0.031327	5.53988E-12
C-reactive protein	rs4766962	12	120863235	T	A	-0.023211	1.33999E-09
C-reactive protein	rs4766965	12	120942065	A	G	-0.067428	1.97002E-09
C-reactive protein	rs4767878	12	120564999	G	A	0.087865	2.1301E-11
C-reactive protein	rs4774590	15	51745277	A	G	-0.022169	2.71E-08
C-reactive protein	rs4803750	19	45247627	G	A	0.057878	1.03992E-12
C-reactive protein	rs4803763	19	45357291	C	G	-0.057938	9.83105E-38
C-reactive protein	rs4841132	8	9183596	G	A	0.065095	1.99986E-25
C-reactive protein	rs4925671	1	247622874	C	T	0.030763	7.85055E-13
C-reactive protein	rs510591	8	9786355	G	A	-0.032837	4.00996E-09
C-reactive protein	rs5112	19	45430280	G	C	-0.07568	1.74985E-24
C-reactive protein	rs6001193	22	39074737	G	A	-0.027809	6.5298E-14
C-reactive protein	rs637649	12	121548922	C	T	0.036583	5.45004E-09
C-reactive protein	rs644234	9	136142217	G	T	0.022597	1.13E-09

C-reactive protein	rs6485751	11	47336442	C	G	-0.03089	1.42988E-12
C-reactive protein	rs6672331	1	65975847	C	G	0.21359	2.84971E-52
C-reactive protein	rs6672992	1	65848034	C	A	0.050172	1.99986E-19
C-reactive protein	rs6684621	1	66315450	T	C	-0.047619	3.63999E-39
C-reactive protein	rs6743376	2	113832333	A	C	0.03238	1.13999E-16
C-reactive protein	rs6859	19	45382034	G	A	0.050489	1.71989E-34
C-reactive protein	rs6982453	8	11641871	C	T	0.019865	4.89001E-08
C-reactive protein	rs707939	6	31726688	A	C	0.024086	4.43997E-10
C-reactive protein	rs7295481	12	103492168	C	T	-0.032369	1.85012E-19
C-reactive protein	rs7310409	12	121424861	G	A	0.137075	2.541E-299
C-reactive protein	rs733228	1	154639618	C	T	0.027671	4.27996E-10
C-reactive protein	rs7349418	2	28443050	T	C	-0.024613	4.44017E-11
C-reactive protein	rs739809	12	120706384	G	C	-0.03861	4.84953E-11
C-reactive protein	rs7521729	1	159844819	A	C	0.037477	9.67008E-10
C-reactive protein	rs7830431	8	10700317	G	A	-0.024819	9.9793E-12
C-reactive protein	rs7837587	8	8378992	C	T	0.026823	3.53021E-14
C-reactive protein	rs7844834	8	11286146	A	C	-0.021957	4.47003E-08
C-reactive protein	rs8179219	2	27730817	A	G	-0.054467	6.00994E-09
C-reactive protein	rs8179228	2	27737637	A	G	-0.108276	2.47999E-08
C-reactive protein	rs862996	1	159168609	T	C	-0.026089	1.43001E-08
C-reactive protein	rs863018	1	159215326	T	C	-0.043948	7.55962E-29
C-reactive protein	rs9271608	6	32591588	G	A	0.042021	2.33024E-17
C-reactive protein	rs9284725	2	102744854	A	C	-0.02731	7.34007E-11
C-reactive protein	rs9329185	8	9204425	G	A	-0.030249	2.4598E-14
C-reactive protein	rs937813	2	28301540	C	T	0.047625	2.05022E-14
C-reactive protein	rs9385532	6	130371227	C	T	0.02556	1.9002E-11
C-reactive protein	rs992153	2	102710355	T	C	-0.022986	2.89001E-09
Creatinine	rs1047891	2	211540507	A	C	-0.020664	6.79516E-17
Creatinine	rs10740991	10	22058137	C	G	0.0163614	1.70012E-10
Creatinine	rs10747486	1	98253738	G	A	0.0153912	2.4391E-08
Creatinine	rs10782651	1	77934335	C	T	0.0135284	5.87151E-09
Creatinine	rs11968400	6	31804729	T	C	-0.028583	1.3241E-08
Creatinine	rs1288775	15	45661678	A	T	-0.016218	7.93524E-10

Creatinine	rs13143189	4	3757380	A	G	0.0151487	9.00534E-11
Creatinine	rs150797189	18	53635086	A	T	0.0373323	3.42934E-08
Creatinine	rs150952847	9	5691403	G	C	-0.048265	2.80021E-08
Creatinine	rs17391694	1	78623626	T	C	-0.020771	3.37388E-10
Creatinine	rs1800437	19	46181392	C	G	0.017439	1.91832E-09
Creatinine	rs2472297	15	75027880	T	C	0.027145	1.41579E-25
Creatinine	rs28399448	19	41352936	T	C	-0.047965	1.27965E-09
Creatinine	rs34262842	16	20355811	G	A	0.0178355	8.45006E-09
Creatinine	rs35041900	1	171451621	T	C	-0.022255	2.08051E-08
Creatinine	rs3788337	22	23412017	A	G	0.0143972	2.36946E-09
Creatinine	rs3815455	7	75611756	T	C	0.0156922	5.95552E-10
Creatinine	rs429150	6	32075563	C	T	-0.012739	3.65671E-08
Creatinine	rs4410790	7	17284577	C	T	0.0210338	1.31947E-18
Creatinine	rs4488444	16	50914706	G	A	-0.015307	1.23174E-08
Creatinine	rs56099375	8	77372988	T	C	0.0154564	9.16854E-09
Creatinine	rs61780440	1	41490761	C	G	-0.01787	1.26151E-09
Creatinine	rs7142377	14	29635571	A	G	-0.017942	1.11294E-09
Creatinine	rs71658797	1	77967507	A	T	-0.02351	2.04268E-11
Creatinine	rs762279	22	24897743	A	G	0.0130058	2.53571E-08
Creatinine	rs784257	18	53397199	C	T	-0.025129	2.18575E-17
Creatinine	rs9271377	6	32587165	G	T	0.0134248	1.88547E-08
Creatinine	rs9673084	15	75622943	A	G	0.016214	3.80391E-10
Albumin	rs3768321	1	40035928	T	G	-0.0315	1.5E-09
Albumin	rs57274629	1	66110292	G	A	0.0286584	4.60045E-11
Albumin	rs9616	1	154555733	T	A	0.0271068	2.30001E-09
Albumin	rs11589479	1	155033308	A	G	0.0349953	2.69998E-10
Albumin	rs34754216	1	161575538	T	C	-0.030255	1.80011E-12
Albumin	rs10737488	1	161619363	C	G	0.0513234	4.00037E-11
Albumin	rs58546652	1	247601778	T	C	-0.025358	3.09999E-09
Albumin	rs1260326	2	27730940	C	T	-0.044408	7.39946E-26
Albumin	rs6734238	2	113841030	G	A	-0.024645	4.90004E-09
Albumin	rs11128594	3	12303417	G	A	-0.035989	6.20012E-12
Albumin	rs13108218	4	3443931	G	A	-0.028153	4.60045E-11

Albumin	rs55881006	4	74222782	A	G	-0.069592	1.2E-09
Albumin	rs55772354	4	74276150	A	G	-0.080945	1.89998E-08
Albumin	rs6871112	5	72125370	G	C	-0.039301	2.09991E-21
Albumin	rs114949263	7	150498245	C	T	0.0499753	3.29989E-14
Albumin	rs1461729	8	9187242	G	A	0.0603088	1.69981E-18
Albumin	rs3740688	11	47380340	T	G	0.0321874	8.49963E-15
Albumin	rs12364432	11	47902883	A	G	-0.026017	2E-09
Albumin	rs4929885	11	49094571	A	G	-0.023357	2.99999E-08
Albumin	rs2933243	12	56860577	A	G	0.0303081	1.40001E-08
Albumin	rs648997	12	111976776	T	C	0.026539	3.2E-08
Albumin	rs17696736	12	112486818	G	A	-0.022892	4.20001E-08
Albumin	rs60333700	14	94467549	T	C	0.0716423	1.29987E-14
Albumin	rs12896856	14	94616285	C	T	0.0342477	6.79986E-11
Albumin	rs11622647	14	94639887	C	G	0.0333127	3.69999E-09
Albumin	rs61983483	14	94640607	T	C	0.0366204	2.99985E-16
Albumin	rs11621961	14	94769476	T	C	0.045893	4.90004E-27
Albumin	rs1950657	14	94804737	G	A	0.0307524	1.10002E-13
Albumin	rs4900227	14	94831363	C	G	0.0346906	9.49948E-17
Albumin	rs28929474	14	94844947	T	C	0.428915	7.2946E-185
Albumin	rs17580	14	94847262	A	T	0.145105	7.19946E-51
Albumin	rs1980618	14	94852423	T	A	0.0241764	2.59998E-08
Albumin	rs72692809	14	94904542	T	C	0.0490453	1.59993E-13
Albumin	rs116933761	14	95011569	A	G	0.0590474	3.29989E-13
Albumin	rs143875230	15	43278726	A	G	0.0926229	4.79954E-12
Albumin	rs139974673	15	44027885	C	T	0.120695	1.9002E-20
Albumin	rs144972973	15	44564692	G	A	0.102333	1.99986E-14
Albumin	rs4886992	15	78325229	C	T	-0.031666	5.69994E-10
Albumin	rs9912287	17	1630992	A	G	0.0415659	1.20005E-16
Albumin	rs200489612	17	7106378	A	G	-0.184315	2.5E-09
Albumin	rs72829457	17	7568327	T	C	0.0433745	3.19963E-11
Albumin	rs74892229	17	16854480	A	G	-0.051466	4.60045E-14
Albumin	rs61653336	17	40296923	A	G	-0.03696	3.19963E-11
Albumin	rs142925250	17	43563606	T	A	-0.029292	3.50002E-08

Albumin	rs77542162	17	67081278	G	A	-0.156008	2.80027E-29
Albumin	rs477031	17	67345501	T	C	0.0445767	7.10003E-09
Albumin	rs536500508	18	57150129	T	A	0.0458459	2.19999E-08
Albumin	rs34284056	18	60203855	A	C	0.026938	6.49995E-09
Albumin	rs8107347	19	18612748	A	G	-0.025805	2.80001E-09
Albumin	rs1672981	19	35547051	C	T	0.0823067	3.29989E-23
Albumin	rs58895965	19	35551428	A	C	0.0633893	3.50026E-31
Albumin	rs35228868	19	49969240	T	G	0.145829	3.79997E-10
Albumin	rs141409212	19	50006402	T	C	0.141396	2.80027E-11
Albumin	rs187804944	19	50016300	A	T	-0.073479	1.59993E-15
Albumin	rs142385484	19	50016759	T	C	0.0619075	9.09913E-26
Albumin	rs150420714	19	50017538	C	G	-0.226064	1.20005E-23
Albumin	rs117080418	19	50025208	A	T	-0.128004	7.10003E-10
Albumin	rs186451245	19	50034227	T	C	0.17507	1.99986E-13
Albumin	rs10419198	19	50038017	T	C	-0.055925	2.70023E-32
Albumin	rs59601394	19	50199540	C	G	-0.048906	1.6E-09
Education of years	rs301800	1	8490603	T	c	0.019	1.79E-08
Education of years	rs11210860	1	43982527	A	g	0.017	2.36E-10
Education of years	rs34305371	1	72733610	A	g	0.035	3.76E-14
Education of years	rs2568955	1	72762169	T	c	-0.017	0.000000018
Education of years	rs1008078	1	91189731	T	c	-0.016	6.01E-10
Education of years	rs11588857	1	204587047	A	g	0.02	5.27E-10
Education of years	rs1777827	1	211613114	A	g	0.015	1.55E-08
Education of years	rs2992632	1	243503764	A	t	0.017	8.23E-09
Education of years	rs76076331	2	10977585	T	c	0.02	3.63E-08
Education of years	rs11689269	2	15621917	C	g	0.016	1.28E-08
Education of years	rs1606974	2	51873599	A	g	0.022	0.000000028
Education of years	rs11690172	2	57387094	A	g	0.015	1.99E-08
Education of years	rs2457660	2	60757419	T	c	-0.017	7.11E-10
Education of years	rs114598875	2	60976384	A	g	-0.02	2.41E-08
Education of years	rs10496091	2	61482261	A	g	-0.018	5.62E-10
Education of years	rs13402908	2	100333377	T	c	-0.018	1.7E-11
Education of years	rs12987662	2	100821548	A	c	0.027	2.69E-24

Education of years	rs17824247	2	144152539	T	c	-0.016	2.77E-09
Education of years	rs16845580	2	161920884	T	c	0.016	2.65E-09
Education of years	rs4500960	2	162818621	T	c	-0.016	3.75E-10
Education of years	rs6739979	2	193731929	T	c	-0.015	0.000000047
Education of years	rs2245901	2	194296294	A	g	-0.016	4.54E-09
Education of years	rs55830725	2	237056854	A	t	-0.022	5.37E-10
Education of years	rs35761247	3	48623124	A	g	0.034	3.82E-08
Education of years	rs62259535	3	48939052	A	g	0.048	2.63E-09
Education of years	rs148734725	3	49406708	A	g	0.025	1.36E-18
Education of years	rs11712056	3	49914397	T	c	0.024	3.3E-19
Education of years	rs112634398	3	50075494	A	g	0.036	4.61E-08
Education of years	rs62263923	3	85674790	A	g	-0.016	7.01E-09
Education of years	rs6799130	3	160847801	C	g	-0.015	2.82E-08
Education of years	rs12646808	4	3249828	T	c	0.016	0.00000004
Education of years	rs2610986	4	18037231	T	c	-0.016	2.01E-08
Education of years	rs34072092	4	28801221	T	c	0.024	3.91E-08
Education of years	rs3101246	4	42649935	T	g	-0.015	1.43E-08
Education of years	rs4863692	4	140764124	T	g	0.018	1.56E-10
Education of years	rs4493682	5	45188024	C	g	0.019	3.32E-08
Education of years	rs2964197	5	57535206	T	c	0.015	3.02E-08
Education of years	rs61160187	5	60111579	A	g	-0.017	3.49E-10
Education of years	rs10061788	5	87934707	A	g	0.021	2.46E-09
Education of years	rs2431108	5	103947968	T	c	0.016	5.27E-09
Education of years	rs1402025	5	113987898	T	c	0.017	3.42E-08
Education of years	rs62379838	5	120102028	T	c	0.016	0.000000033
Education of years	rs56231335	6	98187291	T	c	-0.017	2.07E-09
Education of years	rs9320913	6	98584733	A	c	0.024	2.46E-19
Education of years	rs7767938	6	153367613	T	c	0.017	2.44E-08
Education of years	rs2615691	7	23402104	A	g	-0.037	4.71E-08
Education of years	rs12531458	7	39090698	A	c	0.014	3.11E-08
Education of years	rs12671937	7	92654365	A	g	0.016	9.15E-10
Education of years	rs113520408	7	128402782	A	g	0.017	1.97E-08
Education of years	rs17167170	7	133302345	A	g	0.02	1.14E-09

Education of years	rs11768238	7	135227513	A	g	-0.017	9.9E-10
Education of years	rs12682297	8	145712860	A	t	-0.016	3.93E-09
Education of years	rs1871109	9	1746016	T	g	-0.016	4.35E-10
Education of years	rs13294439	9	23358875	A	c	-0.023	2.2E-17
Education of years	rs895606	9	88003668	A	g	0.015	2.25E-08
Education of years	rs7854982	9	124644562	T	c	-0.015	1.29E-08
Education of years	rs11191193	10	103802408	A	g	0.018	5.44E-11
Education of years	rs12772375	10	104082688	T	g	-0.015	1.56E-08
Education of years	rs7945718	11	12748819	A	g	0.015	1.54E-08
Education of years	rs7955289	12	14653667	A	t	0.017	4.49E-10
Education of years	rs2456973	12	56416928	A	c	-0.02	1.06E-12
Education of years	rs7131944	12	92159557	A	t	0.015	9.02E-09
Education of years	rs572016	12	121279083	A	g	0.014	3.46E-08
Education of years	rs7306755	12	123767929	A	g	0.023	1.26E-12
Education of years	rs9537821	13	58402771	A	g	0.024	1.5E-16
Education of years	rs1043209	14	23373986	A	g	0.018	1.82E-11
Education of years	rs17119973	14	84913111	A	g	-0.019	3.55E-10
Education of years	rs12969294	18	35186122	A	g	-0.016	7.24E-09
Education of years	rs2837992	21	42620520	T	c	0.015	0.000000038
Education of years	rs165633	22	29880773	A	g	-0.018	2.86E-09
Cigarettes smoked per day	rs11264100	1	35591626	A	G	-0.02217	2.22E-09
Cigarettes smoked per day	rs2072659	1	155000000	C	G	-0.02998	2.51E-13
Cigarettes smoked per day	rs34973462	1	176000000	C	T	0.015073	5.85E-09
Cigarettes smoked per day	rs7599488	2	60718347	C	T	0.014121	8.95E-09
Cigarettes smoked per day	rs78408772	2	62710608	C	T	-0.022	4.51E-08
Cigarettes smoked per day	rs10204824	2	148000000	A	G	-0.01798	1.35E-12
Cigarettes smoked per day	rs2084533	3	16872929	C	T	0.016118	6.53E-10

Cigarettes smoked per day	rs7431710	3	48935583	G	A	-0.01829	1.04E-12
Cigarettes smoked per day	rs2236951	3	50421081	T	C	-0.01719	1.59E-08
Cigarettes smoked per day	rs699165	3	136000000	A	G	0.016119	8.09E-09
Cigarettes smoked per day	rs28813180	3	158000000	G	A	-0.0155	1.95E-10
Cigarettes smoked per day	rs1024323	4	3006043	C	T	-0.01442	8.66E-09
Cigarettes smoked per day	rs11940255	4	67086288	G	A	-0.01716	2.2E-10
Cigarettes smoked per day	rs10454798	4	67980830	G	T	0.015842	1.53E-08
Cigarettes smoked per day	rs7766641	6	26184102	G	A	-0.01728	2.91E-10
Cigarettes smoked per day	rs215600	7	32333642	G	A	-0.024	4.02E-21
Cigarettes smoked per day	rs62447179	7	50339609	G	A	-0.01527	9.68E-09
Cigarettes smoked per day	rs2741351	8	27418040	A	C	0.018476	8.8E-09
Cigarettes smoked per day	rs73229090	8	27442127	C	A	0.026211	1.14E-11
Cigarettes smoked per day	rs13253502	8	42442018	G	A	-0.01384	2.31E-08
Cigarettes smoked per day	rs4236926	8	42578059	T	G	0.034267	7.66E-33
Cigarettes smoked per day	rs790564	8	64604218	A	C	-0.01762	1.24E-10
Cigarettes smoked per day	rs75596189	9	136000000	C	T	0.0358	1.84E-20
Cigarettes smoked per day	rs3025383	9	137000000	T	C	-0.03138	9.78E-24
Cigarettes smoked per day	rs7951365	11	16377044	T	C	0.017756	1.53E-11

Cigarettes smoked per day	rs10742683	11	43667625	G	A	-0.01349	4.83E-08
Cigarettes smoked per day	rs113001570	11	46737412	A	T	0.029801	1.04E-09
Cigarettes smoked per day	rs7125588	11	113000000	A	G	-0.0169	6.5E-12
Cigarettes smoked per day	rs11846838	14	104000000	G	A	0.015177	5.03E-09
Cigarettes smoked per day	rs1115019	15	57141231	T	C	-0.01786	2.27E-09
Cigarettes smoked per day	rs632811	15	59155050	A	G	-0.01775	1.67E-10
Cigarettes smoked per day	rs4886550	15	78243579	A	G	-0.01993	4.58E-09
Cigarettes smoked per day	rs10519203	15	78814046	G	A	-0.09362	3.1E-286
Cigarettes smoked per day	rs182317	15	89943601	G	T	-0.01559	1.31E-09
Cigarettes smoked per day	rs1592485	16	52093549	C	A	-0.01615	1.11E-10
Cigarettes smoked per day	rs12924872	16	69552215	C	T	-0.01341	4.39E-08
Cigarettes smoked per day	rs258321	16	89756473	A	G	0.015796	1.53E-10
Cigarettes smoked per day	rs4144686	18	53251725	G	A	-0.01855	1.35E-08
Cigarettes smoked per day	rs4485470	18	62125063	G	A	-0.01527	7.05E-10
Cigarettes smoked per day	rs59208569	19	4044424	G	C	0.020476	2.45E-10
Cigarettes smoked per day	rs143200968	19	41338847	G	C	-0.0861	6.97E-28
Cigarettes smoked per day	rs117824460	19	41371480	A	G	-0.09526	7.66E-35
Cigarettes smoked per day	rs6078373	20	11863500	G	A	0.016073	9.4E-11

Cigarettes smoked per day	rs1737894	20	31054702	C	G	0.016856	9.9E-12
Cigarettes smoked per day	rs2273500	20	61986949	T	C	0.036386	3.49E-26
Cigarettes smoked per day	rs7281463	21	40520783	A	C	0.013678	3.15E-08
Smoking initiation	rs12130857	1	7791461	G	A	-0.018003	3.65E-11
Smoking initiation	rs301807	1	8484823	A	G	0.0180144	2.5E-12
Smoking initiation	rs3820277	1	18436657	G	T	-0.018837	1.57E-13
Smoking initiation	rs1889571	1	32195819	T	G	0.0221798	4.19E-09
Smoking initiation	rs10914684	1	33795572	G	A	-0.015804	6.32E-09
Smoking initiation	rs2637869	1	38757237	G	A	0.0182201	6.54E-11
Smoking initiation	rs12755632	1	41776623	A	G	-0.015405	1.93E-08
Smoking initiation	rs951740	1	44011737	G	A	0.0295409	3.82E-29
Smoking initiation	rs925524	1	46496709	A	G	0.0155573	2.94E-08
Smoking initiation	rs12022778	1	50603995	A	C	0.0268248	3.18E-17
Smoking initiation	rs11587399	1	50861071	A	T	-0.017805	7.25E-09
Smoking initiation	rs4912332	1	58815243	C	T	0.0141209	2.94E-08
Smoking initiation	rs1937443	1	66469643	C	G	0.0204362	1.79E-15
Smoking initiation	rs1022528	1	71490122	G	A	0.0174022	8.48E-11
Smoking initiation	rs12740789	1	72752073	G	A	-0.028497	1.18E-17
Smoking initiation	rs80054503	1	72900406	T	C	-0.024138	3.1E-09
Smoking initiation	rs10789369	1	73824909	A	G	-0.023448	3.39E-19
Smoking initiation	rs1514176	1	74991596	G	A	-0.0193	7.67E-14
Smoking initiation	rs10873871	1	76689019	A	G	0.0174521	2.82E-08
Smoking initiation	rs11162019	1	87913176	C	T	-0.015495	5.06E-09
Smoking initiation	rs1008078	1	91189731	C	T	0.0228169	1.63E-18
Smoking initiation	rs1935571	1	96414335	T	G	-0.01572	6.99E-10
Smoking initiation	rs12027999	1	154206358	T	C	-0.024359	5.33E-10
Smoking initiation	rs45444697	1	155034632	C	G	0.0196901	2.72E-10
Smoking initiation	rs2901785	1	174104743	G	A	-0.017308	1.47E-11
Smoking initiation	rs147052174	1	179783167	G	T	0.0623094	2.3E-10
Smoking initiation	rs35656245	1	190957480	G	A	0.0159456	2.23E-08

Smoking initiation	rs12739243	1	210302043	T	C	-0.021252	4.45E-12
Smoking initiation	rs12563365	1	236872829	G	A	0.0165589	1.05E-10
Smoking initiation	rs876793	1	237852083	T	C	-0.017925	5.69E-11
Smoking initiation	rs114976176	2	264621	A	C	-0.015514	6.04E-09
Smoking initiation	rs62106258	2	417167	T	C	-0.045498	3.33E-14
Smoking initiation	rs6731872	2	624205	T	G	0.0315977	5.35E-21
Smoking initiation	rs1022376	2	22067213	T	C	-0.014745	1.66E-08
Smoking initiation	rs61533748	2	22582968	T	C	0.0174357	2.82E-11
Smoking initiation	rs72790288	2	29513404	G	A	-0.045532	3.28E-09
Smoking initiation	rs2710634	2	32808804	T	C	-0.017761	3.36E-12
Smoking initiation	rs62137126	2	44250149	A	G	-0.023691	1.31E-09
Smoking initiation	rs1004787	2	45159091	G	A	0.0284143	1.11E-28
Smoking initiation	rs7598402	2	50735943	C	G	-0.014728	7.38E-09
Smoking initiation	rs10490159	2	51341259	C	T	0.0172365	3.86E-11
Smoking initiation	rs1518393	2	58171220	A	C	0.0168601	1.3E-10
Smoking initiation	rs17616642	2	59022210	A	G	-0.016557	0.000000021
Smoking initiation	rs6730325	2	59315828	G	A	-0.014636	0.000000021
Smoking initiation	rs2539706	2	59819545	G	A	0.0162454	1.95E-10
Smoking initiation	rs7585579	2	60024857	C	G	0.0203962	5.48E-15
Smoking initiation	rs1863161	2	60139524	G	A	0.0153394	2.34E-09
Smoking initiation	rs359247	2	60477052	A	T	0.0220304	9.89E-17
Smoking initiation	rs62180324	2	63416606	G	A	-0.019517	3.91E-10
Smoking initiation	rs6750107	2	80748807	G	A	0.0145651	0.000000026
Smoking initiation	rs12714017	2	80999398	T	C	0.0153965	3.65E-09
Smoking initiation	rs56208390	2	83247997	A	G	0.0215636	2.68E-08
Smoking initiation	rs11692435	2	98275354	G	A	0.0250526	4.47E-08
Smoking initiation	rs13392222	2	100672408	A	C	-0.023438	1.93E-10
Smoking initiation	rs1901477	2	104126983	A	G	0.030437	2.07E-31
Smoking initiation	rs11889814	2	104432494	A	C	-0.021027	3.44E-08
Smoking initiation	rs3811038	2	113240183	T	C	0.0191404	1.58E-11
Smoking initiation	rs75210106	2	113246436	C	T	-0.018659	2.33E-08
Smoking initiation	rs34399632	2	137571174	A	G	0.0193504	1.46E-10
Smoking initiation	rs74697736	2	145412271	G	A	0.0222963	2.43E-15

Smoking initiation	rs6756212	2	146140132	C	T	-0.033888	3.49E-40
Smoking initiation	rs16826827	2	147825689	T	C	-0.022207	9.17E-09
Smoking initiation	rs1445649	2	155682556	T	C	0.0205716	8.48E-16
Smoking initiation	rs1722666	2	161816880	C	T	0.0160932	2.17E-08
Smoking initiation	rs11678980	2	162101261	G	A	0.0176689	5.19E-12
Smoking initiation	rs12474587	2	162802993	G	T	0.0242306	4.83E-21
Smoking initiation	rs357304	2	164862639	T	C	0.0166757	5.4E-09
Smoking initiation	rs13007361	2	166250244	G	A	0.0175338	2.29E-08
Smoking initiation	rs7600835	2	172521827	G	A	-0.015122	0.000000018
Smoking initiation	rs6750529	2	182027603	C	T	0.0199074	9.26E-12
Smoking initiation	rs17229285	2	199523122	C	T	-0.01548	1.27E-09
Smoking initiation	rs3115418	2	200936399	T	C	-0.014225	2.79E-08
Smoking initiation	rs62193862	2	202843875	G	A	0.0238463	1.99E-08
Smoking initiation	rs4674916	2	225365635	C	A	-0.018026	3.06E-11
Smoking initiation	rs4674993	2	226332033	A	G	-0.024005	4.85E-14
Smoking initiation	rs11713899	3	2365026	A	C	0.0187188	3.15E-08
Smoking initiation	rs748832	3	16851202	A	G	0.0172142	6.6E-11
Smoking initiation	rs10446419	3	25725501	A	G	-0.019563	5.05E-10
Smoking initiation	rs13319205	3	47800216	T	A	0.0165398	3.77E-09
Smoking initiation	rs3172494	3	48731487	G	T	-0.029127	3.4E-13
Smoking initiation	rs2526390	3	50192760	C	T	0.0204663	3.62E-14
Smoking initiation	rs2276825	3	52886605	T	C	0.0188756	1.89E-10
Smoking initiation	rs2306866	3	53766212	A	T	-0.016675	1.89E-10
Smoking initiation	rs73831818	3	55988394	A	G	0.0320435	5.46E-09
Smoking initiation	rs1910236	3	59434420	G	A	0.0146439	9.91E-09
Smoking initiation	rs7640107	3	59966156	C	T	-0.014186	3.46E-08
Smoking initiation	rs2734390	3	60459291	A	G	0.0147709	2.09E-08
Smoking initiation	rs221988	3	64234307	A	C	-0.014865	1.43E-08
Smoking initiation	rs2196356	3	70890288	G	C	-0.018773	2.45E-11
Smoking initiation	rs11128203	3	71064431	T	A	0.020406	1.29E-15
Smoking initiation	rs62246017	3	71483084	G	A	-0.016169	3.03E-09
Smoking initiation	rs4543050	3	74954560	A	T	0.0222035	1.45E-11
Smoking initiation	rs6782116	3	77176032	C	T	-0.01465	1.46E-08

Smoking initiation	rs13066050	3	81325861	C	T	0.0188343	1.93E-09
Smoking initiation	rs12633090	3	83241365	G	C	-0.02302	3.16E-12
Smoking initiation	rs1549979	3	85460131	C	T	-0.024522	8.8E-21
Smoking initiation	rs74664784	3	85475292	T	C	-0.019911	9.34E-13
Smoking initiation	rs57153235	3	85902536	T	G	-0.01938	1.56E-12
Smoking initiation	rs6437769	3	107997514	C	T	0.0142141	3.74E-08
Smoking initiation	rs9288999	3	114147927	G	A	0.0174411	1.5E-09
Smoking initiation	rs6438436	3	117822149	C	T	0.024737	5.33E-14
Smoking initiation	rs12053870	3	118302515	T	G	0.0156164	1.02E-09
Smoking initiation	rs9826984	3	131945722	G	A	-0.014053	3.87E-08
Smoking initiation	rs2279829	3	147106319	C	T	-0.017377	2.05E-08
Smoking initiation	rs2319545	3	147719648	C	A	0.0232366	8.3E-11
Smoking initiation	rs10935779	3	149543102	C	T	-0.014327	2.95E-08
Smoking initiation	rs963354	3	157393770	C	A	0.0150489	4.21E-08
Smoking initiation	rs1714521	3	158284861	A	C	-0.016295	3.07E-10
Smoking initiation	rs1449012	3	159048333	C	T	-0.015373	1.77E-09
Smoking initiation	rs9850597	3	161761866	G	A	-0.018571	1.65E-08
Smoking initiation	rs1187820	3	173072584	C	T	-0.014271	2.69E-08
Smoking initiation	rs16828799	3	173353739	G	T	0.0197692	1.83E-08
Smoking initiation	rs9841807	3	175718927	C	T	0.0162535	1.35E-08
Smoking initiation	rs7631379	3	181409057	T	C	0.0208014	3.94E-11
Smoking initiation	rs4140932	4	15458598	T	A	-0.014045	4.89E-08
Smoking initiation	rs12642744	4	28027176	G	T	-0.016591	2.82E-08
Smoking initiation	rs59537158	4	28246049	C	T	0.0224875	4.62E-13
Smoking initiation	rs1389171	4	28822284	T	A	-0.017472	4.45E-09
Smoking initiation	rs55944129	4	29082156	T	C	-0.017565	1.06E-09
Smoking initiation	rs58400863	4	31184484	G	A	-0.020172	4.89E-14
Smoking initiation	rs55900829	4	35514712	A	T	0.0191319	5.63E-12
Smoking initiation	rs112725451	4	68017710	C	T	0.026092	1.65E-14
Smoking initiation	rs1160685	4	94052854	C	G	0.0153017	2.31E-09
Smoking initiation	rs1435479	4	94550450	G	T	0.0163905	5.68E-09
Smoking initiation	rs3934797	4	112467612	G	A	-0.021297	1.12E-10
Smoking initiation	rs71602617	4	136406155	C	T	-0.017765	0.000000021

Smoking initiation	rs7696257	4	137474783	G	A	0.0153313	6.78E-09
Smoking initiation	rs13109980	4	140886963	G	A	-0.022182	3.37E-16
Smoking initiation	rs1116690	4	143510148	A	G	0.0162912	2.16E-08
Smoking initiation	rs13110073	4	147797913	T	C	-0.024643	3.24E-21
Smoking initiation	rs28717373	4	147985231	C	T	-0.016466	6.16E-10
Smoking initiation	rs62340589	4	176875795	G	C	0.0174134	4.31E-08
Smoking initiation	rs12517438	5	30842054	T	G	0.0153545	1.89E-09
Smoking initiation	rs35375873	5	43190647	G	C	-0.02701	3.29E-11
Smoking initiation	rs986714	5	50821338	A	T	-0.016031	4.13E-10
Smoking initiation	rs71592686	5	60121271	T	C	0.0207374	3.85E-13
Smoking initiation	rs2028269	5	79308315	G	A	0.0161645	5.19E-10
Smoking initiation	rs6874731	5	80263865	T	G	0.0153179	1.83E-09
Smoking initiation	rs6452785	5	87685500	C	T	-0.026883	4.69E-26
Smoking initiation	rs10805858	5	88873832	A	T	0.0181241	1.88E-11
Smoking initiation	rs181508347	5	91366274	T	G	0.0810758	4.95E-10
Smoking initiation	rs42417	5	94198290	C	T	0.0169296	8.27E-10
Smoking initiation	rs72780746	5	103929588	T	C	-0.025763	2.05E-14
Smoking initiation	rs10060196	5	106455988	C	A	0.0183116	1.29E-12
Smoking initiation	rs72789626	5	106825618	T	A	-0.025643	5.13E-12
Smoking initiation	rs17165769	5	107365642	A	G	0.0159396	9.56E-10
Smoking initiation	rs329124	5	133865452	A	G	-0.016387	1.96E-10
Smoking initiation	rs1385108	5	154839646	C	T	0.0187045	3.84E-10
Smoking initiation	rs1173461	5	157707571	C	T	0.0166092	9.51E-10
Smoking initiation	rs11956866	5	161018271	T	G	-0.014838	7.82E-09
Smoking initiation	rs3909281	5	165096435	T	G	0.0210672	1.62E-16
Smoking initiation	rs3843905	5	165427280	C	T	-0.015146	5.41E-09
Smoking initiation	rs79476395	5	166063680	A	G	0.0333743	1.04E-11
Smoking initiation	rs6890961	5	166778503	C	T	-0.019311	2.13E-13
Smoking initiation	rs4044321	5	166989513	A	G	-0.022641	1.75E-17
Smoking initiation	rs2173019	5	167614971	T	A	0.028207	2.98E-17
Smoking initiation	rs10042827	5	170299916	T	C	0.0167171	9.41E-10
Smoking initiation	rs359431	5	173288534	C	T	-0.014198	3.16E-08
Smoking initiation	rs1059490	6	26171250	T	C	-0.018594	2.16E-12

Smoking initiation	rs6932350	6	26571629	T	A	0.0149684	5.13E-09
Smoking initiation	rs1150668	6	28129789	T	G	-0.01851	8.54E-13
Smoking initiation	rs1632941	6	29796685	T	C	-0.015807	6.67E-10
Smoking initiation	rs3218116	6	41901763	C	T	-0.019843	1.05E-11
Smoking initiation	rs160631	6	52895230	T	G	-0.017263	1.87E-09
Smoking initiation	rs7743165	6	67521222	T	G	0.0192559	4.15E-14
Smoking initiation	rs79180767	6	67540984	C	T	0.0200919	7E-12
Smoking initiation	rs10945141	6	69470709	G	A	0.0181417	3.59E-10
Smoking initiation	rs17554906	6	92226609	G	C	0.0141849	3.14E-08
Smoking initiation	rs619087	6	94175279	A	G	0.0142703	0.000000031
Smoking initiation	rs6568832	6	97702876	G	A	0.018869	1.74E-10
Smoking initiation	rs12195240	6	98636905	G	A	0.0249108	1.08E-18
Smoking initiation	rs6936160	6	100347745	C	T	0.0201068	4.2E-13
Smoking initiation	rs12530388	6	101329173	A	C	-0.018362	5.83E-13
Smoking initiation	rs3800227	6	108994161	A	G	0.017178	3.64E-09
Smoking initiation	rs118202	6	111658371	G	T	-0.036748	1.9E-29
Smoking initiation	rs73008357	6	156431856	A	C	-0.02231	2.44E-08
Smoking initiation	rs9331343	6	157738258	T	C	-0.014135	0.000000039
Smoking initiation	rs10698713	6	158882320	G	A	-0.033517	2.38E-09
Smoking initiation	rs1737329	6	163807748	C	G	0.0170292	5.08E-09
Smoking initiation	rs10272990	7	1703675	T	C	-0.020922	1.27E-14
Smoking initiation	rs6948707	7	1870794	T	G	0.0243472	4.24E-21
Smoking initiation	rs10259715	7	3329967	T	A	-0.018674	6.42E-09
Smoking initiation	rs13237637	7	3503207	G	C	-0.023682	1.54E-20
Smoking initiation	rs79631993	7	69432311	A	C	-0.017034	3.67E-08
Smoking initiation	rs7809303	7	69484366	G	A	-0.021419	3.48E-15
Smoking initiation	rs7802996	7	77771983	C	T	-0.020885	1.06E-09
Smoking initiation	rs1030015	7	78139581	G	T	0.0142896	2.15E-08
Smoking initiation	rs4727189	7	88442568	T	C	0.0148604	0.00000003
Smoking initiation	rs76841737	7	91281409	C	G	-0.023149	3.26E-08
Smoking initiation	rs11768481	7	96629103	C	A	-0.018556	5.23E-12
Smoking initiation	rs1799068	7	97707069	G	T	0.0166096	2.59E-10
Smoking initiation	rs13437771	7	99071478	A	G	-0.02711	1.39E-14

Smoking initiation	rs11766326	7	111100585	T	C	-0.017544	1.79E-11
Smoking initiation	rs6968380	7	114940159	G	A	-0.023419	1.05E-17
Smoking initiation	rs112913817	7	115077394	A	G	0.0780558	9.28E-11
Smoking initiation	rs10233018	7	117523709	A	G	0.0246124	4.77E-22
Smoking initiation	rs10953957	7	121954709	G	A	0.0144058	3.66E-08
Smoking initiation	rs77283305	7	132593831	G	A	-0.015196	3.91E-08
Smoking initiation	rs10279261	7	133589846	G	A	-0.018873	6.05E-13
Smoking initiation	rs1561112	7	133840652	T	C	-0.015244	3.84E-09
Smoking initiation	rs2952251	8	10143164	A	G	0.0164125	4.24E-08
Smoking initiation	rs4326350	8	10763655	C	G	-0.017614	5.16E-12
Smoking initiation	rs11783093	8	27425349	C	T	-0.047124	2.07E-41
Smoking initiation	rs7836565	8	52569449	C	T	-0.015508	4.36E-08
Smoking initiation	rs13261666	8	59814666	G	T	-0.019995	4.36E-15
Smoking initiation	rs3850736	8	64912021	C	G	0.0191284	6.43E-14
Smoking initiation	rs2063976	8	91096366	C	T	-0.020181	7.45E-14
Smoking initiation	rs6993429	8	92733282	C	A	-0.01905	9.87E-14
Smoking initiation	rs6986430	8	93048104	T	C	-0.024338	1.99E-15
Smoking initiation	rs9987376	8	93190014	T	G	-0.020468	2.01E-15
Smoking initiation	rs290601	8	115374642	C	T	0.0163102	1.14E-08
Smoking initiation	rs3847244	9	3025368	C	T	0.0186717	2.6E-13
Smoking initiation	rs11791671	9	3398679	C	T	0.0278504	4.24E-08
Smoking initiation	rs7024924	9	8282399	T	C	0.0188918	0.000000019
Smoking initiation	rs6474609	9	10981069	T	A	-0.015589	1.71E-09
Smoking initiation	rs1931431	9	11161799	G	C	0.0182329	8.56E-13
Smoking initiation	rs7867822	9	20676454	A	G	-0.015097	2.76E-08
Smoking initiation	rs10966092	9	23831658	T	C	-0.020486	1.12E-12
Smoking initiation	rs10969352	9	29747488	T	A	0.0143467	1.82E-08
Smoking initiation	rs4877285	9	81354129	G	A	-0.018132	2.1E-11
Smoking initiation	rs1930371	9	81444104	C	T	-0.017242	7.09E-09
Smoking initiation	rs2378662	9	86707289	G	A	0.0152121	2.67E-09
Smoking initiation	rs1927901	9	120519111	T	C	-0.014175	0.000000031
Smoking initiation	rs4837631	9	122061948	C	T	-0.015357	2.03E-09
Smoking initiation	rs1759433	9	128073097	G	A	0.0153647	1.69E-09

Smoking initiation	rs34553878	9	134334588	A	G	0.0246707	1.17E-09
Smoking initiation	rs7026534	9	134907263	T	G	-0.016603	2.68E-09
Smoking initiation	rs10858334	9	137989785	C	G	0.022871	1.18E-09
Smoking initiation	rs10905461	10	8803551	T	C	-0.016392	2.36E-08
Smoking initiation	rs7920501	10	10043159	T	A	-0.015517	1.25E-09
Smoking initiation	rs1291821	10	11133823	A	G	0.014493	1.39E-08
Smoking initiation	rs11258417	10	13533053	C	T	-0.014514	2.71E-08
Smoking initiation	rs7072776	10	22032942	A	G	-0.021975	5.66E-15
Smoking initiation	rs2796793	10	36634124	G	A	0.0144814	1.55E-08
Smoking initiation	rs1733760	10	56698174	T	C	0.0147734	6.7E-09
Smoking initiation	rs7921378	10	63674885	G	C	-0.023314	6.1E-20
Smoking initiation	rs7901883	10	103186838	G	A	-0.019257	1.98E-10
Smoking initiation	rs11594623	10	103960351	T	C	0.0274396	7.45E-20
Smoking initiation	rs11191269	10	104120522	C	G	0.0176426	4.61E-08
Smoking initiation	rs28408682	10	104403310	A	G	0.0166729	1.41E-10
Smoking initiation	rs12244388	10	104640052	G	A	0.0258153	4.31E-22
Smoking initiation	rs111842178	10	104852121	A	G	0.0224527	2.24E-12
Smoking initiation	rs34970111	10	106078937	C	T	-0.014556	1.28E-08
Smoking initiation	rs9787523	10	106460460	T	C	-0.015627	1.42E-09
Smoking initiation	rs11192347	10	106929313	G	A	-0.02645	6.15E-10
Smoking initiation	rs10885480	10	115378364	T	C	-0.018677	3.83E-11
Smoking initiation	rs4752018	10	118678712	C	A	0.0188538	4.42E-10
Smoking initiation	rs9423279	10	125680419	C	G	-0.018581	3.06E-12
Smoking initiation	rs6265	11	27679916	C	T	-0.029275	2.81E-19
Smoking initiation	rs4275621	11	28652996	A	G	-0.021367	3.76E-16
Smoking initiation	rs62618693	11	32956492	C	T	-0.035272	2.09E-08
Smoking initiation	rs2939756	11	41436297	G	A	-0.0157	7.45E-10
Smoking initiation	rs1381775	11	42442826	T	C	-0.015615	2.79E-08
Smoking initiation	rs2959084	11	46078656	G	A	0.0170799	9.82E-10
Smoking initiation	rs3740977	11	46393574	T	C	0.019474	1.17E-08
Smoking initiation	rs61886926	11	64133552	C	T	-0.01794	7.3E-12
Smoking initiation	rs61884449	11	64485193	C	T	0.0199753	2.32E-08
Smoking initiation	rs644740	11	65561468	C	T	-0.014079	3.67E-08

Smoking initiation	rs7943721	11	73309393	G	A	-0.021214	3.58E-10
Smoking initiation	rs7929518	11	85980958	A	G	0.0192363	2.55E-10
Smoking initiation	rs586699	11	92289734	G	A	-0.014803	7.29E-09
Smoking initiation	rs76460663	11	111979741	C	G	-0.04235	4.15E-11
Smoking initiation	rs2155646	11	112912811	T	C	0.0377772	9.44E-48
Smoking initiation	rs78239456	11	112984491	A	T	-0.018495	9.37E-12
Smoking initiation	rs1713676	11	113660576	A	G	-0.016726	5.38E-11
Smoking initiation	rs238896	11	113994505	G	A	-0.016867	3.65E-11
Smoking initiation	rs540860	11	121530888	A	G	0.0176086	5.75E-12
Smoking initiation	rs1944689	11	121634334	G	T	0.01768	1.27E-08
Smoking initiation	rs1834306	11	122023187	A	G	-0.014485	1.96E-08
Smoking initiation	rs1106363	11	131966264	C	T	0.0173746	9.2E-11
Smoking initiation	rs2010921	11	132098205	G	A	0.0174291	2.47E-10
Smoking initiation	rs11057005	12	16748721	A	G	-0.015714	9.12E-10
Smoking initiation	rs13906	12	49952394	C	T	-0.02453	1.98E-09
Smoking initiation	rs4759229	12	56474480	A	G	0.0155696	6.53E-09
Smoking initiation	rs7969559	12	69655167	A	G	-0.017016	1.53E-09
Smoking initiation	rs7134009	12	75263193	T	C	-0.015797	0.000000043
Smoking initiation	rs77215829	12	112618346	A	C	-0.024045	2.02E-10
Smoking initiation	rs1109480	12	121083279	G	A	-0.016692	1.84E-10
Smoking initiation	rs11611651	12	133380790	G	A	0.0271143	2.05E-09
Smoking initiation	rs17197663	13	38172867	G	A	-0.021587	2.06E-08
Smoking initiation	rs4264267	13	38359676	C	T	0.0147921	6.82E-09
Smoking initiation	rs61959481	13	55834929	G	A	-0.020344	7.95E-11
Smoking initiation	rs3098272	13	55931424	A	C	-0.017808	2.08E-08
Smoking initiation	rs9538162	13	59265043	T	C	0.0173792	1.76E-11
Smoking initiation	rs1413119	13	59339281	C	T	-0.015255	4.77E-09
Smoking initiation	rs56367474	13	59454139	C	T	-0.017298	4.2E-10
Smoking initiation	rs55786907	13	59871584	A	G	0.0194452	1.84E-08
Smoking initiation	rs4886207	13	60705792	T	C	-0.016247	8.78E-10
Smoking initiation	rs9540731	13	66949370	C	T	-0.01773	3.42E-12
Smoking initiation	rs9545155	13	80191873	T	C	-0.016071	3.04E-10
Smoking initiation	rs1772572	13	81191176	C	A	-0.016868	5.62E-10

Smoking initiation	rs75674569	13	96823724	G	A	-0.025338	2.58E-09
Smoking initiation	rs7333559	13	100546450	G	A	-0.023213	5.94E-14
Smoking initiation	rs1108130	13	100648356	T	A	0.0239436	1.57E-14
Smoking initiation	rs12855717	13	101252635	C	T	0.0155237	1.22E-09
Smoking initiation	rs12878369	14	28346502	C	A	0.0174373	1.6E-11
Smoking initiation	rs2145451	14	29316842	T	C	-0.020046	5.44E-10
Smoking initiation	rs9323328	14	58653514	A	G	-0.014237	2.55E-08
Smoking initiation	rs1811739	14	77529375	G	A	0.0182718	5.97E-10
Smoking initiation	rs8005334	14	79563654	T	G	0.0166734	3.44E-10
Smoking initiation	rs34940743	14	80102233	A	G	0.0159249	2.8E-09
Smoking initiation	rs2925128	14	98362355	C	T	0.0168199	3.67E-10
Smoking initiation	rs1381287	14	98597552	C	T	0.0180166	1.81E-12
Smoking initiation	rs55913542	14	99693843	G	T	0.0185623	3.25E-08
Smoking initiation	rs1435672	15	36399479	T	C	0.0141051	3.82E-08
Smoking initiation	rs281296	15	47685010	G	A	0.0246892	1.59E-20
Smoking initiation	rs1435741	15	47935843	G	A	0.0183091	1.09E-12
Smoking initiation	rs56902655	15	63898709	T	G	-0.021863	4.09E-09
Smoking initiation	rs2289791	15	67476952	G	T	-0.017726	2.01E-09
Smoking initiation	rs60833441	15	74048768	A	G	-0.014277	2.28E-08
Smoking initiation	rs62007780	15	78025464	G	T	-0.015913	7.48E-10
Smoking initiation	rs12442563	15	83893243	G	T	-0.02323	3.13E-14
Smoking initiation	rs4310804	15	96858409	C	G	-0.018187	7.55E-10
Smoking initiation	rs8027457	15	99204101	T	C	0.0153138	1.88E-09
Smoking initiation	rs1139897	16	720986	G	A	-0.024087	1.77E-15
Smoking initiation	rs11076962	16	5811367	T	C	0.0182999	1.2E-10
Smoking initiation	rs7192140	16	10173748	T	C	-0.016883	3.4E-11
Smoking initiation	rs9922607	16	17570220	C	T	-0.022159	3.42E-12
Smoking initiation	rs9941217	16	18050926	C	G	-0.018557	3.5E-12
Smoking initiation	rs7188873	16	24727064	A	G	0.0202958	8.46E-15
Smoking initiation	rs6497840	16	25351633	G	A	0.0227703	2.01E-15
Smoking initiation	rs4785187	16	49766772	G	A	0.0199772	6.55E-11
Smoking initiation	rs8050598	16	49891964	C	T	0.0186698	1.76E-10
Smoking initiation	rs12918191	16	50945156	A	G	-0.019727	3.14E-11

Smoking initiation	rs9302604	16	69576894	A	G	0.0187095	3.29E-13
Smoking initiation	rs9936784	16	72230694	T	G	0.0139895	4.33E-08
Smoking initiation	rs62052916	16	72574550	A	T	-0.031914	1.62E-10
Smoking initiation	rs4788676	16	72950468	T	C	-0.017745	4.92E-09
Smoking initiation	rs61537885	16	75620118	T	C	-0.040063	8.06E-09
Smoking initiation	rs117657830	16	75766873	A	G	-0.03776	3.18E-09
Smoking initiation	rs1050847	16	87443734	C	T	-0.01483	7.37E-09
Smoking initiation	rs11642231	16	89608702	G	A	-0.015598	3.44E-09
Smoking initiation	rs4790874	17	1995177	C	T	0.0174491	8.43E-12
Smoking initiation	rs11078713	17	7795972	A	G	-0.014583	1.59E-08
Smoking initiation	rs28441558	17	7803118	T	C	-0.035565	1.24E-10
Smoking initiation	rs11651955	17	16235462	G	A	-0.014026	3.74E-08
Smoking initiation	rs67777803	17	27323322	G	T	-0.024601	3.18E-13
Smoking initiation	rs2344976	17	30685935	T	C	-0.015088	7.98E-09
Smoking initiation	rs3764351	17	37824339	G	A	-0.014749	3.89E-08
Smoking initiation	rs72836318	17	44121579	T	C	-0.017122	0.000000007
Smoking initiation	rs17692129	17	44793283	C	T	0.0195989	4.57E-13
Smoking initiation	rs75919030	17	50193197	T	C	-0.020966	3.35E-13
Smoking initiation	rs2938134	17	50243397	C	A	-0.017501	3.14E-10
Smoking initiation	rs2587507	17	77790135	T	C	-0.01466	8.69E-09
Smoking initiation	rs34342129	18	5872472	T	C	-0.014281	2.13E-08
Smoking initiation	rs4476253	18	25253297	G	A	-0.018486	5.78E-10
Smoking initiation	rs7505855	18	31696075	C	T	-0.016982	5.31E-11
Smoking initiation	rs8096225	18	36921851	A	C	0.0155245	2.63E-08
Smoking initiation	rs67050670	18	39297254	A	G	-0.020272	2.34E-11
Smoking initiation	rs2359180	18	41314171	A	G	-0.014389	4.98E-08
Smoking initiation	rs72898831	18	42658643	A	G	-0.024416	4.14E-12
Smoking initiation	rs8083764	18	49874515	G	T	-0.015952	7.97E-09
Smoking initiation	rs1373178	18	49967811	T	G	-0.020316	4.16E-15
Smoking initiation	rs62098013	18	50863861	G	A	0.01771	2.24E-11
Smoking initiation	rs72938304	18	53661743	G	A	-0.027205	1.36E-11
Smoking initiation	rs11872397	18	72535282	G	A	-0.017114	5.2E-09
Smoking initiation	rs71367544	18	77574374	C	T	0.0205522	8.54E-11

Smoking initiation	rs76608582	19	4474725	C	A	-0.034549	4.88E-09
Smoking initiation	rs10853981	19	4965064	G	A	0.0147872	4.88E-08
Smoking initiation	rs113230003	19	18460956	G	A	-0.018876	1.05E-10
Smoking initiation	rs8103660	19	18566395	T	C	0.0158026	3.03E-09
Smoking initiation	rs117734003	19	51129745	G	C	0.0302991	2.57E-09
Smoking initiation	rs1126757	19	55879872	C	T	0.0141623	2.92E-08
Smoking initiation	rs6050446	20	25195509	A	G	0.0544098	8.8E-13
Smoking initiation	rs6058782	20	29946968	C	T	0.0297103	1.78E-11
Smoking initiation	rs1555445	20	31175258	A	T	0.0187626	7.75E-12
Smoking initiation	rs6073075	20	42015801	T	A	-0.018704	2.44E-08
Smoking initiation	rs910912	20	54462393	T	C	-0.01677	7.82E-09
Smoking initiation	rs6011779	20	61984317	C	T	-0.019177	2.83E-09
Smoking initiation	rs3810496	20	62406886	T	C	0.0158813	1.54E-09
Smoking initiation	rs4818005	21	40588819	G	A	-0.020431	1.09E-14
Smoking initiation	rs139896	22	38397797	T	C	0.0154404	7.14E-09
Smoking initiation	rs4822102	22	42698430	C	T	-0.016543	2.78E-10
Smoking initiation	rs9627272	22	46442288	G	C	-0.015474	2.42E-09
Smoking cessation	rs112187834	2	23953454	T	A	0.0334097	2.81E-09
Smoking cessation	rs7617480	3	49210732	A	C	-0.032871	1.68E-12
Smoking cessation	rs12203592	6	396321	C	T	-0.029209	1.21E-08
Smoking cessation	rs707968	6	35058117	A	G	0.0232707	2.76E-08
Smoking cessation	rs7778443	7	32314690	T	C	-0.022999	1.04E-08
Smoking cessation	rs1565735	8	27426077	T	A	-0.034557	1.54E-12
Smoking cessation	rs60749569	8	42602668	A	T	-0.040071	2.68E-08
Smoking cessation	rs12378015	9	127917257	G	A	-0.02766	8.31E-11
Smoking cessation	rs9409844	9	136461851	G	A	-0.058561	4.37E-10
Smoking cessation	rs3025327	9	136467344	G	C	0.0785863	1.19E-35
Smoking cessation	rs10821523	9	136473572	A	C	0.0261566	2.28E-11
Smoking cessation	rs1611124	9	136509275	G	T	-0.045338	5.26E-09
Smoking cessation	rs7109376	11	16372431	T	A	0.0280592	1.14E-10
Smoking cessation	rs591143	15	47647755	C	T	-0.02433	1.14E-09
Smoking cessation	rs3866543	15	76629609	T	G	0.0222019	1.35E-08
Smoking cessation	rs518425	15	78883813	A	G	-0.030503	1.72E-12

Smoking cessation	rs145580088	19	41342842	A	G	0.0909551	9.48E-13
Smoking cessation	rs56113850	19	41353107	T	C	-0.057606	1.61E-48
Smoking cessation	rs117824460	19	41371480	A	G	0.0864992	1.09E-12
Smoking cessation	rs6011779	20	61984317	C	T	-0.050022	9.89E-24
Smoking cessation	rs6089904	20	62018289	A	T	-0.064183	4.01E-12
Smoking cessation	rs9607805	22	41854446	C	T	0.0295408	1.37E-11
Coffee consumption	rs17685	7	75454041	A	G	0.07	4.26E-11
Coffee consumption	rs4410790	7	17251102	T	C	0.1	3.08E-17
Coffee consumption	rs7800944	7	72673793	T	C	0.05	2.29E-11
Coffee consumption	rs2472297	15	72814933	T	C	0.14	2.47E-24
Coffee consumption	rs9902453	17	25373221	A	G	0.03	2.44E-08
Morningness	rs61773390	1	7884525	T	G	0.029	2.75E-27
Morningness	rs12065331	1	14507831	T	C	-0.015	1.51E-10
Morningness	rs17448682	1	15966713	T	C	0.018	4.04E-13
Morningness	rs7543480	1	20014827	T	C	0.016	6.9E-13
Morningness	rs10916866	1	21137768	A	C	-0.017	2.39E-14
Morningness	rs12140153	1	62579891	T	G	-0.027	4.62E-12
Morningness	rs11208844	1	66851147	A	G	-0.017	2.81E-08
Morningness	rs11162296	1	77700196	C	G	0.036	1.5E-34
Morningness	rs17416934	1	79874811	T	C	0.013	5.93E-09
Morningness	rs72720396	1	91191582	A	G	-0.022	3.29E-18
Morningness	rs7522677	1	96470261	T	C	0.015	7.91E-09
Morningness	rs9437742	1	96960983	C	G	-0.012	3.53E-08
Morningness	rs12139650	1	97598100	T	G	0.015	2.21E-08
Morningness	rs10494041	1	110031990	C	G	0.02	1.01E-12
Morningness	rs35461065	1	115060826	T	C	-0.012	8.36E-09
Morningness	rs2794682	1	150320847	T	C	0.021	3.93E-22
Morningness	rs75650221	1	174421994	T	C	0.034	8.92E-10
Morningness	rs13306728	1	179312559	A	G	0.03	6.16E-14
Morningness	rs509476	1	182573227	T	C	0.099	7.41E-56
Morningness	rs12746073	1	183679912	T	C	0.012	1.73E-08
Morningness	rs12025393	1	193341118	A	G	-0.014	1.82E-08
Morningness	rs16839841	1	196353384	T	G	0.022	2.69E-08

Morningness	rs13011556	2	4651923	C	G	-0.016	7.73E-11
Morningness	rs2712056	2	23959131	T	C	0.018	1.57E-10
Morningness	rs848552	2	36700580	C	G	-0.013	1.11E-09
Morningness	rs2592199	2	44710169	C	G	0.022	1.35E-14
Morningness	rs10495976	2	49750698	A	T	-0.017	1.01E-13
Morningness	rs10193431	2	53725767	T	C	0.013	4.35E-09
Morningness	rs17049270	2	58192905	T	C	-0.02	1.96E-08
Morningness	rs10175975	2	59429807	T	C	0.018	1.94E-10
Morningness	rs359237	2	60473094	T	C	-0.015	9.18E-12
Morningness	rs4672440	2	61616653	T	G	0.016	5.09E-13
Morningness	rs113851554	2	66750564	T	G	-0.028	1.32E-08
Morningness	rs2706762	2	70488470	T	C	-0.019	3.01E-10
Morningness	rs7586062	2	77305160	C	G	-0.022	3.06E-24
Morningness	rs10190053	2	77916762	A	C	-0.013	6.51E-09
Morningness	rs75863239	2	105312975	T	C	-0.023	3.21E-08
Morningness	rs62172117	2	144168667	A	G	-0.019	1.43E-17
Morningness	rs7579662	2	161915810	A	G	-0.012	2.24E-08
Morningness	rs13004345	2	174037347	T	C	-0.012	3.76E-08
Morningness	rs11677484	2	191578172	T	G	0.015	6.25E-10
Morningness	rs4850712	2	197301425	T	G	0.013	9.4E-09
Morningness	rs6716898	2	198944271	A	G	0.021	2.32E-22
Morningness	rs184033703	2	206956138	A	G	0.032	9.65E-10
Morningness	rs11900963	2	239308049	A	T	0.045	3.22E-32
Morningness	rs62182135	2	240267305	A	C	-0.013	7.8E-09
Morningness	rs17786957	3	2550093	C	G	-0.018	6.05E-10
Morningness	rs7428484	3	23330101	A	G	0.013	1.97E-08
Morningness	rs62263597	3	50131691	A	G	0.028	4.79E-13
Morningness	rs67000219	3	71557581	T	C	-0.019	2.68E-08
Morningness	rs9876864	3	77208521	A	T	0.016	9.2E-14
Morningness	rs55753638	3	85644482	T	C	-0.022	2.86E-11
Morningness	rs1800828	3	113891549	C	G	0.014	5.32E-09
Morningness	rs6799356	3	123148362	A	C	0.014	4.14E-08
Morningness	rs2699869	3	133032892	A	C	0.013	5.06E-09

Morningness	rs1109088	3	138154795	A	G	0.013	2.6E-09
Morningness	rs6769642	3	160763469	A	C	0.016	2.8E-13
Morningness	rs3850174	3	172364093	A	T	-0.014	9.06E-09
Morningness	rs6443810	3	182262672	C	G	0.015	2.47E-10
Morningness	rs6778003	3	185991970	T	G	0.016	4.94E-12
Morningness	rs9683585	4	2702804	C	G	-0.012	1.79E-08
Morningness	rs56040212	4	16435084	A	G	-0.014	0.000000022
Morningness	rs28634184	4	62873419	T	C	-0.014	2.31E-08
Morningness	rs57180764	4	83239773	A	G	0.017	3.22E-11
Morningness	rs4241964	4	137053959	T	G	-0.015	3.54E-12
Morningness	rs7449161	5	7214040	T	G	0.013	2.2E-09
Morningness	rs3797051	5	63860141	T	C	0.015	1.48E-09
Morningness	rs542867945	5	65743706	A	C	0.49	4.29E-08
Morningness	rs66507804	5	86630284	T	C	-0.017	1.47E-10
Morningness	rs304137	5	88169652	A	G	0.016	3.06E-14
Morningness	rs40465	5	103981726	T	G	-0.013	2.46E-08
Morningness	rs286808	5	107459376	T	C	0.013	3.87E-09
Morningness	rs10052000	5	152298890	C	G	0.02	7.5E-19
Morningness	rs42210	5	166408788	C	G	-0.014	3.53E-09
Morningness	rs335433	5	176867207	T	C	-0.013	2.29E-09
Morningness	rs9395520	6	13183523	T	C	0.02	1.6E-18
Morningness	rs9295795	6	29145623	T	C	-0.026	0.000000019
Morningness	rs486416	6	31856070	A	G	-0.013	6.07E-09
Morningness	rs734597	6	50836279	A	G	0.016	1.62E-08
Morningness	rs2653349	6	55142337	A	G	0.031	1.03E-32
Morningness	rs2881955	6	72479263	T	C	0.016	5.64E-11
Morningness	rs9375352	6	98739938	A	T	0.014	2.03E-08
Morningness	rs4557564	6	110232051	A	G	0.025	2.15E-09
Morningness	rs6935086	6	128944677	T	C	0.02	5.87E-09
Morningness	rs9479402	6	153135339	T	C	-0.102	1.35E-22
Morningness	rs9348050	6	166263488	T	C	0.013	0.000000002
Morningness	rs16873715	7	8560454	A	T	-0.015	4.42E-08
Morningness	rs56382918	7	24067993	T	C	0.018	1.13E-12

Morningness	rs10236197	7	32291761	T	C	0.014	2.03E-10
Morningness	rs56049037	7	32947201	A	G	-0.017	2.28E-12
Morningness	rs4245555	7	50661409	T	C	-0.018	1.96E-15
Morningness	rs2138759	7	71823436	A	G	0.014	1.75E-09
Morningness	rs2922966	7	96455598	A	G	0.025	1.11E-17
Morningness	rs202157	7	101637753	T	C	-0.018	1.21E-14
Morningness	rs112613078	7	102433678	A	G	-0.025	1.55E-20
Morningness	rs10262462	7	114180062	A	G	-0.015	3.18E-12
Morningness	rs6978514	7	115684409	T	C	-0.013	4.87E-08
Morningness	rs2971970	7	133643778	T	G	0.017	2.23E-11
Morningness	rs35748596	8	4825443	T	G	-0.016	2.37E-12
Morningness	rs34344642	8	31765586	T	G	-0.024	2.88E-09
Morningness	rs12541362	8	33584661	A	T	-0.018	5.55E-15
Morningness	rs1919346	8	35211457	A	G	0.012	0.000000013
Morningness	rs11988076	8	53128629	A	G	-0.019	1.82E-11
Morningness	rs6472936	8	76639759	T	C	0.018	5.16E-12
Morningness	rs1110275	8	86854332	T	C	0.02	4.05E-08
Morningness	rs34578339	8	89440842	A	T	-0.016	2.84E-08
Morningness	rs72673588	8	93356390	C	G	-0.017	1.26E-09
Morningness	rs3100052	8	101967139	A	G	0.013	0.000000013
Morningness	rs2737245	8	116658583	T	G	0.018	2.94E-14
Morningness	rs1323591	9	8456020	T	C	-0.016	2.11E-12
Morningness	rs2291589	9	37079661	T	G	0.018	1.65E-16
Morningness	rs77598468	9	76484268	A	C	-0.042	1.05E-12
Morningness	rs4565536	9	85189088	A	C	-0.012	4.97E-08
Morningness	rs10797119	9	92202495	T	C	-0.013	2.8E-09
Morningness	rs10818834	9	126317324	T	C	0.014	5.24E-09
Morningness	rs28365587	9	131959615	A	G	-0.013	3.6E-09
Morningness	rs10448340	9	139320069	T	G	-0.013	9.07E-09
Morningness	rs1750785	10	776150	A	G	0.014	3.52E-09
Morningness	rs9416744	10	60567937	A	C	0.017	1.47E-12
Morningness	rs7910164	10	72852735	A	G	0.012	4.83E-08
Morningness	rs76518095	10	131149976	T	C	0.023	0.00000001

Morningness	rs9795439	11	1483543	A	G	0.018	6.68E-09
Morningness	rs925947	11	27667367	T	G	0.016	9.64E-10
Morningness	rs12799529	11	30408552	T	C	0.018	5.13E-13
Morningness	rs11032362	11	33759092	A	G	0.031	3.46E-17
Morningness	rs34239319	11	43902681	T	G	0.024	6.45E-12
Morningness	rs11039308	11	47622412	A	G	0.014	4.94E-11
Morningness	rs3168135	11	58386177	A	G	-0.017	3.64E-12
Morningness	rs4008953	11	66660949	A	G	-0.016	3.23E-11
Morningness	rs4237555	11	92725803	T	C	0.012	4.19E-08
Morningness	rs4936290	11	114009255	A	C	-0.013	1.18E-08
Morningness	rs577924	11	122135107	T	C	0.012	2.07E-08
Morningness	rs1174510	12	17093317	A	G	0.012	4.86E-08
Morningness	rs11611435	12	24089322	T	C	0.012	0.00000004
Morningness	rs13377754	12	34051765	T	C	0.024	7.84E-27
Morningness	rs7313852	12	38908389	A	G	-0.025	1.02E-29
Morningness	rs11183201	12	46170982	T	C	-0.017	1.6E-14
Morningness	rs671255	12	52189566	A	G	-0.014	1.42E-08
Morningness	rs7299922	12	54702519	A	G	0.013	8.56E-09
Morningness	rs9788226	12	63517356	C	G	0.022	1.25E-11
Morningness	rs7488974	12	90442001	A	G	0.016	1.81E-13
Morningness	rs10861694	12	107405922	T	C	0.014	2.6E-10
Morningness	rs77531286	12	114299548	T	C	-0.017	0.000000014
Morningness	rs4102203	12	120976371	T	C	0.024	7.8E-12
Morningness	rs61963123	13	42512639	T	C	0.017	2.33E-10
Morningness	rs9597250	13	56303709	A	C	-0.019	2.88E-12
Morningness	rs2321993	13	59681146	A	G	-0.014	2.59E-11
Morningness	rs2593487	13	69903058	A	G	-0.014	2.88E-10
Morningness	rs9565309	13	77577027	T	C	0.066	1.26E-28
Morningness	rs7337911	13	94111843	A	G	0.015	0.000000001
Morningness	rs9521184	13	109782568	T	C	0.013	8.34E-10
Morningness	rs11157143	14	41010556	A	G	-0.015	8.23E-10
Morningness	rs56376592	14	57378912	A	C	0.017	1.4E-09
Morningness	rs4899502	14	74669893	A	G	-0.016	1.61E-11

Morningness	rs12432176	14	101021218	A	C	0.013	1.47E-08
Morningness	rs2701524	15	37383688	T	C	0.013	5.39E-09
Morningness	rs59986227	15	48009263	C	G	-0.015	5.36E-09
Morningness	rs12442008	15	53725112	T	C	0.014	3.36E-08
Morningness	rs11852820	15	101150435	C	G	-0.014	5.76E-10
Morningness	rs2304467	16	8988777	C	G	-0.012	3.91E-08
Morningness	rs7196720	16	24534662	T	C	0.013	4.32E-09
Morningness	rs12927162	16	52684916	A	G	0.028	2.11E-32
Morningness	rs1421085	16	53800954	T	C	-0.023	1.39E-25
Morningness	rs2398144	16	56352854	A	C	-0.021	2.42E-21
Morningness	rs8044054	16	60628436	T	C	0.015	3.87E-12
Morningness	rs17604349	16	72210865	A	G	-0.022	1.21E-15
Morningness	rs2518022	17	8057367	T	C	0.032	1.53E-16
Morningness	rs2232839	17	17399635	T	C	-0.025	2.41E-21
Morningness	rs9915731	17	30599555	A	T	-0.013	0.000000028
Morningness	rs225289	17	33928945	T	C	0.016	1.24E-08
Morningness	rs72828815	17	42994794	T	C	0.016	6.08E-09
Morningness	rs117974417	17	46156510	C	G	-0.018	1.54E-09
Morningness	rs6504758	17	50208366	A	G	-0.014	9.83E-11
Morningness	rs9898091	17	54173599	T	C	0.038	6.88E-12
Morningness	rs8072058	17	55734198	A	T	-0.015	1.38E-08
Morningness	rs17682747	17	61181112	A	G	0.014	4.69E-08
Morningness	rs2916142	17	65482064	T	C	0.015	7.63E-12
Morningness	rs10491171	17	68414995	C	G	0.019	3.62E-09
Morningness	rs487952	17	74898300	A	G	0.013	4.43E-09
Morningness	rs974552	18	5186164	A	G	-0.022	1.4E-15
Morningness	rs1013987	18	22630836	T	C	-0.015	1.36E-11
Morningness	rs4239386	18	31664710	A	T	-0.019	2.06E-17
Morningness	rs12969848	18	38152835	T	C	0.017	1.52E-15
Morningness	rs9956387	18	44773382	A	T	-0.014	1.38E-10
Morningness	rs17596722	18	53154167	T	C	-0.018	3.24E-11
Morningness	rs9964420	18	56824041	A	C	-0.022	4.03E-21
Morningness	rs11152350	18	60240352	A	C	-0.015	1.07E-12

Morningness	rs9958145	18	64363800	A	G	-0.015	4.26E-08
Morningness	rs10402849	19	2695661	T	C	0.016	6.73E-09
Morningness	rs3843751	19	10748121	T	C	0.013	6.33E-09
Morningness	rs9636202	19	18449238	A	G	-0.013	3.53E-08
Morningness	rs12481462	20	14728972	T	C	0.012	4.36E-08
Morningness	rs78095690	20	16239683	T	C	-0.013	5.75E-09
Morningness	rs6131942	20	17348608	A	G	-0.015	7.63E-12
Morningness	rs1737893	20	31051699	T	C	-0.014	1.37E-09
Morningness	rs2072727	20	43538733	T	C	0.012	3.46E-08
Morningness	rs695459	22	28848278	T	C	-0.013	6.59E-09
Morningness	rs28580373	22	35847288	A	G	0.014	3.25E-08
Morningness	rs11705370	22	40555993	A	T	-0.019	3.43E-15
Morningness	rs55715427	22	42458827	C	G	-0.022	1.35E-09
Morningness	rs2294203	22	45738487	A	G	0.013	2.97E-08
Sleep duration	rs4642942	1	31492150	C	G	0.01	3.07E-08
Sleep duration	rs915416	1	34731984	C	G	0.02	8.15E-12
Sleep duration	rs540431	1	57859521	G	A	-0.01	4.91E-09
Sleep duration	rs1392817	1	66539456	A	G	0.01	1.84E-08
Sleep duration	rs79512144	2	9188514	A	G	-0.03	3.64E-08
Sleep duration	rs116219610	2	58849728	T	C	0.02	4.75E-14
Sleep duration	rs62158206	2	114084596	C	T	0.04	3E-43
Sleep duration	rs35662245	2	147583187	A	T	0.02	6.23E-09
Sleep duration	rs11883686	2	157049217	T	A	-0.01	1.01E-08
Sleep duration	rs12463754	2	166942663	G	C	0.02	3.91E-10
Sleep duration	rs112230981	3	55879269	G	A	-0.03	4.59E-08
Sleep duration	rs4688116	3	118195601	G	T	-0.01	0.000000042
Sleep duration	rs16834426	3	123283309	A	G	0.02	1.76E-08
Sleep duration	rs9843801	3	136000746	C	T	0.02	5.82E-10
Sleep duration	rs2192528	4	18327896	A	G	0.02	6.27E-10
Sleep duration	rs7686205	4	44535866	G	A	0.01	1.37E-08
Sleep duration	rs41501452	4	82272105	G	A	-0.02	4.04E-09
Sleep duration	rs17285646	4	92557496	A	T	0.01	1.88E-08
Sleep duration	rs13109404	4	102896591	G	T	-0.03	1.13E-11

Sleep duration	rs365663	5	1428883	G	A	-0.02	1.12E-11
Sleep duration	rs6889592	5	102313939	A	G	0.02	1.44E-11
Sleep duration	rs113322698	5	137769576	A	G	0.01	1.94E-08
Sleep duration	rs12215241	6	27023081	A	G	-0.02	6.09E-10
Sleep duration	rs34388845	6	28578286	G	A	-0.02	5.43E-10
Sleep duration	rs1633063	6	29726046	T	C	-0.02	3.17E-09
Sleep duration	rs9451146	6	89798679	T	C	-0.02	8.53E-09
Sleep duration	rs9362971	6	93157567	C	T	-0.01	1.41E-08
Sleep duration	rs3823624	7	2110346	C	T	0.02	1.3E-09
Sleep duration	rs6979198	7	107197694	G	T	0.02	1.07E-08
Sleep duration	rs1668331	7	113872935	T	G	-0.02	5.2E-10
Sleep duration	rs12336359	9	4129657	C	G	0.01	9.42E-09
Sleep duration	rs10973207	9	37100525	T	G	0.02	2.02E-09
Sleep duration	rs144625846	10	104078044	G	A	-0.02	1.05E-08
Sleep duration	rs7915425	10	125016501	T	C	0.02	3.08E-10
Sleep duration	rs7115856	11	43769287	C	A	0.01	1.25E-09
Sleep duration	rs12791153	11	80685181	T	A	0.03	2.89E-08
Sleep duration	rs1553132	11	88297740	G	A	0.02	6.63E-09
Sleep duration	rs1263056	11	116576415	G	A	-0.01	4.15E-08
Sleep duration	rs4767550	12	117951150	G	A	0.01	2.7E-09
Sleep duration	rs11621908	14	78495761	T	C	-0.03	4.95E-09
Sleep duration	rs13329140	15	48050577	A	G	-0.02	7.51E-11
Sleep duration	rs77684884	16	6499120	G	A	0.02	5.83E-10
Sleep duration	rs28651105	16	19987636	G	A	0.02	4.75E-08
Sleep duration	rs8047587	16	53798622	T	G	-0.02	1.09E-12
Sleep duration	rs11076146	16	56261429	G	T	0.01	2.96E-09
Sleep duration	rs11654671	17	4732467	T	A	0.02	3.71E-08
Sleep duration	rs11650677	17	11231513	A	G	0.01	8.95E-09
Sleep duration	rs62061734	17	44018488	C	T	-0.02	2.17E-09
Sleep duration	rs12607679	18	53059748	C	T	-0.02	9.79E-11
Getting up	rs61773374	1	7858108	G	A	0.02	3.37E-14
Getting up	rs301806	1	8482078	C	T	-0.01	4.73E-08
Getting up	rs77576965	1	15916734	T	C	0.01	1.94E-08

Getting up	rs12752290	1	21535330	C	T	0.01	1.02E-12
Getting up	rs113240734	1	77699071	A	G	0.02	2.17E-16
Getting up	rs75650221	1	174421994	T	C	0.04	3.93E-18
Getting up	rs4652514	1	180529542	C	T	-0.01	9.91E-09
Getting up	rs12736689	1	182549729	C	T	0.05	1.66E-24
Getting up	rs76048411	2	4650079	T	C	0.01	8.53E-10
Getting up	rs2053457	2	44572164	C	T	-0.02	1.29E-15
Getting up	rs1402121	2	50596607	C	T	0.01	6.66E-11
Getting up	rs4671328	2	58935282	T	G	0.01	1.06E-09
Getting up	rs10175975	2	59429807	T	C	0.01	3.3E-09
Getting up	rs13393656	2	70119255	A	C	0.01	0.000000021
Getting up	rs406952	2	76307345	C	T	0.01	0.000000012
Getting up	rs4853283	2	77156402	G	A	-0.01	1.84E-16
Getting up	rs1606803	2	88933316	T	C	0.01	3.22E-10
Getting up	rs4483990	2	239423098	C	A	-0.02	6.4E-13
Getting up	rs13116306	4	10727697	T	C	-0.01	1.76E-08
Getting up	rs10470887	4	92567442	G	A	-0.01	4.05E-08
Getting up	rs9995419	4	158607383	A	G	0.01	0.000000015
Getting up	rs79751662	4	171014655	C	G	-0.02	2.32E-08
Getting up	rs1459192	5	63944519	T	C	-0.01	0.000000025
Getting up	rs12515274	5	87655662	A	G	-0.01	5.37E-10
Getting up	rs4958316	5	151942251	A	C	0.02	3.69E-15
Getting up	rs553108	6	31840455	A	G	0.01	3.12E-08
Getting up	rs2653349	6	55142337	A	G	0.02	8.45E-29
Getting up	rs9399613	6	147986024	T	C	-0.01	1.32E-08
Getting up	rs3735478	7	44800176	T	G	-0.01	1.57E-08
Getting up	rs2944822	7	71795592	T	C	0.01	4.43E-09
Getting up	rs16917522	8	53136442	C	T	0.01	1.12E-09
Getting up	rs72663537	8	76700618	G	T	-0.01	2.76E-08
Getting up	rs4962716	10	126685867	T	C	-0.02	1.82E-09
Getting up	rs11229264	11	57909086	A	G	-0.01	1.43E-10
Getting up	rs7297799	12	34260142	T	C	-0.01	1.21E-12
Getting up	rs17464772	12	39247777	A	G	0.01	1.03E-11

Getting up	rs2193749	12	46134812	T	C	-0.01	1.84E-08
Getting up	rs6581138	12	57744864	A	G	0.01	2.3E-10
Getting up	rs1017168	12	107435405	A	C	-0.01	2.64E-08
Getting up	rs74643199	12	116772891	T	A	-0.01	1.25E-08
Getting up	rs4884166	13	55769145	A	G	-0.01	1.15E-08
Getting up	rs7332608	13	77570677	G	A	-0.03	4.55E-11
Getting up	rs6575012	14	89056699	A	G	-0.01	2.57E-08
Getting up	rs3935182	15	78094807	G	C	0.01	6.77E-12
Getting up	rs1420607	16	49149191	A	G	0.01	1.57E-11
Getting up	rs11642015	16	53802494	T	C	0.01	6.2E-11
Getting up	rs1949072	16	56006108	A	G	0.01	2.48E-08
Getting up	rs17822102	16	60650143	G	A	0.01	9.4E-10
Getting up	rs11643192	16	72214276	A	C	-0.01	1.3E-09
Getting up	rs4790352	17	2578550	G	A	-0.02	8.78E-09
Getting up	rs3760185	17	17401736	T	C	-0.01	4.52E-08
Getting up	rs7222039	17	38165541	T	C	0.01	3.42E-08
Getting up	rs12150229	17	44015446	G	A	0.01	3.52E-09
Getting up	rs77556405	17	46463909	A	G	0.02	1.59E-13
Getting up	rs12601968	17	50296459	T	G	-0.01	1.16E-09
Getting up	rs4395148	18	31690668	T	A	-0.01	2.36E-08
Getting up	rs8182491	19	42712024	T	C	-0.02	1.18E-09
Getting up	rs3746601	20	30662805	C	A	0.01	2.85E-08
Getting up	rs11697690	20	31477981	C	T	0.01	2.33E-08
Getting up	rs74555583	20	51332268	A	G	-0.02	3.04E-08
Napping	rs2820313	1	201870221	G	A	1.07	1.75E-09
Napping	rs2367277	3	192758964	A	G	1.06	4.94E-09
Napping	rs2653344	6	55133586	T	C	1.07	1.11E-08
Napping	rs34799682	9	81743116	G	A	1.07	2.59E-08
Napping	rs2763895	13	22446756	G	A	1.06	3.72E-08
Napping	rs117124984	17	44051588	G	C	0.91	2.76E-13
Napping	rs2048524	18	44799195	A	G	0.94	9.61E-10
Dozing	rs28600082	4	176247942	T	C	2.44	0.0000108
Snoring	rs35915391	1	87781737	C	G	0.97	0.0000225

Snoring	rs35562935	1	96689205	A	G	1.06	0.0000169
Snoring	rs72906130	2	157076893	G	C	1.06	3.5E-14
Snoring	rs9309771	3	77593064	A	G	1.03	5.65E-09
Snoring	rs34811474	4	25408838	A	G	0.96	4.22E-08
Snoring	rs6855873	4	42539270	T	C	1.03	0.0000208
Snoring	rs2307111	5	75003678	C	T	0.97	1.34E-08
Snoring	rs10062026	5	90052289	A	G	0.97	0.0000208
Snoring	rs745558	5	134453814	A	G	0.97	0.000000266
Snoring	rs947612	6	73738661	G	A	1.03	0.00000992
Snoring	rs17060460	6	100827834	G	A	1.03	0.0000143
Snoring	rs9389081	6	133815465	A	T	0.95	0.0000437
Snoring	rs2981329	8	34985791	C	T	1.03	0.00000774
Snoring	rs7007887	8	71555913	T	C	1.04	9.88E-11
Snoring	rs4523230	8	78234143	A	T	0.97	0.00000167
Snoring	rs1016013	9	97476484	A	G	0.97	0.000000722
Snoring	rs11256034	10	9086147	T	C	1.04	4.67E-08
Snoring	rs2049045	11	27694241	C	G	0.96	8.87E-08
Snoring	rs10878269	12	65791463	T	C	1.04	3.76E-10
Snoring	rs12427782	13	40745813	T	G	0.97	5.77E-09
Snoring	rs2762049	13	50822363	C	G	1.04	8.69E-09
Snoring	rs592333	13	51340315	G	A	1.04	1.08E-13
Snoring	rs2664299	14	99742187	C	T	0.97	4.48E-08
Snoring	rs9933881	16	1740691	C	T	1.06	8.94E-09
Snoring	rs732172	16	31050033	T	C	1.03	0.00000135
Snoring	rs8047587	16	53798622	T	G	1.03	0.00000213
Snoring	rs12449843	17	2058207	A	G	0.97	0.000000445
Snoring	rs1641511	17	7559677	G	A	1.03	0.000014
Snoring	rs57222984	17	43758898	G	A	1.04	3.03E-11
Snoring	rs2924251	17	46338677	A	G	1.03	0.00000517
Snoring	rs180107	17	67930772	A	T	0.97	0.00000386
Snoring	rs4987719	18	60960310	T	C	1.08	0.0000359
Snoring	rs10415992	19	32181118	G	C	0.95	8.14E-09
Snoring	rs34107769	20	46306828	C	T	0.97	0.00000993

Snoring	rs6099273	20	55347828	T	C	1.03	0.0000122
Insomnia	rs113851554	2	66750564	T	G	1.229	1.56E-51
Insomnia	rs62149809	2	73853830	A	G	1.159	5.71E-09
Insomnia	rs1064939	11	118396331	A	T	1.139	2.16E-10
Insomnia	rs79204944	13	53969796	A	G	1.082	4.24E-08
Insomnia	rs72899452	11	45415577	T	C	1.077	0.000000001
Insomnia	rs55972276	5	135653737	A	C	1.076	4.19E-17
Insomnia	rs138014720	3	50070843	A	T	1.072	3.46E-08
Insomnia	rs2286729	12	6873818	A	G	1.072	5.37E-11
Insomnia	rs118166957	9	8858043	T	C	1.07	1.95E-16
Insomnia	rs11650304	17	46035001	C	G	1.069	1.23E-08
Insomnia	rs62158170	2	114082175	A	G	1.068	1.2E-19
Insomnia	rs62264767	3	117642005	A	C	1.067	1.63E-14
Insomnia	rs7168238	15	66709386	C	G	1.066	0.000000018
Insomnia	rs699844	1	74878253	A	G	1.062	4.11E-08
Insomnia	rs7566062	2	66972843	T	C	1.061	1.37E-16
Insomnia	rs28611339	8	10170037	T	G	1.06	8.46E-11
Insomnia	rs1015438	16	51177517	A	G	1.06	2.51E-14
Insomnia	rs6465151	7	88310899	T	C	1.058	1.9E-09
Insomnia	rs2815757	1	72764289	T	C	1.057	2.24E-13
Insomnia	rs16903122	5	87693561	T	C	1.057	9.04E-16
Insomnia	rs2792990	9	125621610	C	G	1.056	1.15E-10
Insomnia	rs62590551	23	21494390	A	G	1.056	4.61E-09
Insomnia	rs34490907	17	26933741	C	G	1.055	1.76E-08
Insomnia	rs670501	7	108625185	T	C	1.054	7.4E-13
Insomnia	rs1927902	9	120518991	T	C	1.054	1.15E-14
Insomnia	rs1620977	1	72729142	A	G	1.053	2.27E-14
Insomnia	rs75452188	2	67134426	A	G	1.053	1.58E-08
Insomnia	rs11756035	6	18843810	C	G	1.052	1.29E-08
Insomnia	rs62429521	6	140324582	A	C	1.052	1.78E-09
Insomnia	rs7992992	13	54721699	A	G	1.052	1.15E-08
Insomnia	rs908668	19	56134038	T	C	1.051	1.41E-11
Insomnia	rs2491124	13	53784083	T	C	1.05	8.81E-16

Insomnia	rs35322724	16	77137324	A	C	1.05	3.75E-16
Insomnia	rs62068188	17	2400876	T	C	1.05	1.18E-09
Insomnia	rs9931543	16	56128782	T	C	1.049	1.11E-12
Insomnia	rs3902952	16	61647589	T	C	1.049	2.55E-10
Insomnia	rs4790076	17	2243628	T	C	1.049	1.76E-09
Insomnia	rs45453598	16	52637892	A	T	1.048	4.42E-09
Insomnia	rs17223714	5	50492629	A	G	1.047	2.44E-10
Insomnia	rs9316619	13	53978628	T	C	1.047	5.5E-09
Insomnia	rs429358	19	45411941	T	C	1.047	2.13E-08
Insomnia	rs830716	16	12323509	C	G	1.046	8.68E-12
Insomnia	rs67501351	16	20006745	C	G	1.046	5.36E-11
Insomnia	rs34214423	16	52303107	A	C	1.046	3.18E-09
Insomnia	rs12614369	2	66792109	A	G	1.045	7.21E-09
Insomnia	rs116466468	2	159137557	T	C	1.045	2.11E-10
Insomnia	rs2903385	4	106094427	A	G	1.044	4.53E-13
Insomnia	rs6606731	12	109982578	A	T	1.044	1.51E-08
Insomnia	rs742760	20	50985290	A	T	1.044	2.48E-08
Insomnia	rs10800992	1	190900576	T	C	1.043	3.84E-12
Insomnia	rs55772859	2	208042581	A	C	1.043	4.82E-11
Insomnia	rs10865954	3	49211989	T	C	1.043	1.92E-11
Insomnia	rs17005118	4	82288564	A	G	1.043	6.13E-10
Insomnia	rs35539975	5	91607148	A	G	1.043	4.49E-09
Insomnia	rs12666306	7	115082406	A	G	1.043	2.24E-12
Insomnia	rs2867690	20	41972028	T	C	1.043	0.000000037
Insomnia	rs12030482	1	96961268	A	T	1.042	8.16E-09
Insomnia	rs17025198	3	88001713	A	G	1.042	2.19E-08
Insomnia	rs56133505	11	72348039	A	G	1.042	5.59E-12
Insomnia	rs7486418	12	84336911	T	G	1.042	6.84E-11
Insomnia	rs715338	15	57215867	A	G	1.042	7.85E-12
Insomnia	rs4643373	17	47123423	T	C	1.042	1.58E-10
Insomnia	rs56097173	2	44262449	T	C	1.041	2.69E-10
Insomnia	rs12991815	2	68071990	C	G	1.041	3.02E-11
Insomnia	rs12187443	5	102660400	T	C	1.041	1.64E-10

Insomnia	rs9373590	6	101212001	A	T	1.041	2.18E-11
Insomnia	rs4592425	11	62697813	T	G	1.041	4.31E-10
Insomnia	rs11149313	13	85294881	A	G	1.041	2.38E-09
Insomnia	rs6019663	20	47774512	T	C	1.041	6.47E-10
Insomnia	rs62194948	2	239222376	C	G	1.04	4.64E-09
Insomnia	rs6808140	3	10581380	T	C	1.04	5.35E-11
Insomnia	rs35110063	3	43066558	A	G	1.04	8.82E-11
Insomnia	rs1147852	6	147980909	A	G	1.04	9.94E-10
Insomnia	rs324017	12	57487814	A	C	1.04	1.61E-09
Insomnia	rs6562066	13	60532796	T	C	1.04	1.38E-10
Insomnia	rs1038093	15	74012409	T	C	1.04	2.47E-10
Insomnia	rs11090039	22	41496800	A	G	1.04	1.82E-09
Insomnia	rs1861412	2	58893065	A	G	1.039	1.67E-10
Insomnia	rs6888135	5	141254063	A	C	1.039	1.21E-10
Insomnia	rs940780	7	3323848	T	C	1.039	8.5E-10
Insomnia	rs12924275	16	9191790	T	C	1.039	1.93E-08
Insomnia	rs2398144	16	56352854	A	C	1.039	5.09E-10
Insomnia	rs11679943	2	77724624	A	G	1.038	3.16E-09
Insomnia	rs6756610	2	147480394	C	G	1.038	1.14E-09
Insomnia	rs62213452	2	210380152	T	G	1.038	2.39E-08
Insomnia	rs7040224	9	134886837	A	G	1.038	4.24E-09
Insomnia	rs72773790	9	139109080	T	C	1.038	3.71E-09
Insomnia	rs5877	1	173878862	T	C	1.037	1.23E-08
Insomnia	rs1530938	2	236900633	A	G	1.037	8.82E-10
Insomnia	rs7625896	3	44062561	A	G	1.037	5.28E-09
Insomnia	rs1264419	6	30576781	C	G	1.037	8.91E-10
Insomnia	rs2737240	8	116657235	A	G	1.037	3.37E-08
Insomnia	rs10756571	9	14534505	T	C	1.037	0.000000018
Insomnia	rs2221119	11	88598444	C	G	1.037	0.000000002
Insomnia	rs9540729	13	66947124	A	T	1.037	1.4E-09
Insomnia	rs34967082	2	215382654	A	G	1.036	4.34E-09
Insomnia	rs2216427	3	180785697	C	G	1.036	0.000000016
Insomnia	rs6601080	5	179511043	A	G	1.036	2.21E-08

Insomnia	rs2598293	7	133989882	T	C	1.036	2.48E-09
Insomnia	rs871994	8	35190619	A	C	1.036	5.5E-09
Insomnia	rs1167132	12	43484487	T	C	1.036	8.73E-09
Insomnia	rs176644	15	89913632	T	G	1.036	9.49E-09
Insomnia	rs12605642	18	31313965	T	G	1.036	2.13E-09
Insomnia	rs9964420	18	56824041	A	C	1.036	4.54E-08
Insomnia	rs72820274	2	104412924	A	G	1.035	1.28E-08
Insomnia	rs10928256	2	146458738	T	C	1.035	1.61E-08
Insomnia	rs4260410	3	178469932	T	C	1.035	4.87E-08
Insomnia	rs11722569	4	112822731	T	C	1.035	2.91E-08
Insomnia	rs13138995	4	148987430	A	G	1.035	1.97E-08
Insomnia	rs6978112	7	1966841	T	C	1.035	2.11E-08
Insomnia	rs2030672	7	21687925	C	G	1.035	0.000000011
Insomnia	rs874168	8	30849450	T	C	1.035	7.95E-09
Insomnia	rs10898940	11	73455292	A	C	1.035	8.09E-09
Insomnia	rs1567084	3	71435955	A	G	1.034	2.14E-08
Insomnia	rs1580173	3	107955515	A	G	1.034	2.28E-08
Insomnia	rs1357685	7	109200331	T	C	1.034	1.39E-08
Insomnia	rs4588900	8	73890425	A	G	1.034	1.57E-08
Insomnia	rs28552587	8	103356226	A	G	1.034	0.000000033
Insomnia	rs10955647	8	114154187	T	G	1.034	1.84E-08
Insomnia	rs6597649	9	133786652	T	C	1.034	3.05E-08
Insomnia	rs10825503	10	57177470	T	G	1.034	1.43E-08
Insomnia	rs667730	11	83277325	T	C	1.034	2.26E-08
Insomnia	rs647905	11	121534938	T	C	1.034	2.87E-08
Insomnia	rs10947987	6	41754370	T	C	0.968	4.08E-08
Insomnia	rs4858708	3	25154112	A	T	0.967	1.23E-08
Insomnia	rs2364921	3	158522463	T	C	0.967	2.13E-08
Insomnia	rs238869	6	29355113	T	C	0.967	3.36E-08
Insomnia	rs190073	7	10985188	A	G	0.967	2.86E-08
Insomnia	rs9563886	13	61720066	T	C	0.967	3.08E-08
Insomnia	rs2447094	17	2294048	A	C	0.967	0.000000025
Insomnia	rs1553754	17	46563707	T	G	0.967	3.51E-08

Insomnia	rs11588755	1	57819204	A	G	0.966	5.14E-09
Insomnia	rs11119409	1	210293333	T	C	0.966	1.19E-08
Insomnia	rs728017	6	124292594	A	G	0.966	9.51E-09
Insomnia	rs1731951	7	137075847	A	T	0.966	1.36E-08
Insomnia	rs4788203	16	29978827	A	G	0.966	6.32E-09
Insomnia	rs12454003	18	26315799	C	G	0.966	4.94E-09
Insomnia	rs910187	20	45841052	A	G	0.966	1.63E-08
Insomnia	rs34036083	2	66815719	T	C	0.965	2.07E-08
Insomnia	rs12520974	5	61514611	T	C	0.965	1.69E-09
Insomnia	rs701394	5	80296487	A	G	0.965	6.83E-09
Insomnia	rs37445	5	106899684	A	G	0.965	4.88E-09
Insomnia	rs17367725	5	107112116	T	C	0.965	9.29E-09
Insomnia	rs9469434	6	33455574	C	G	0.965	4.41E-08
Insomnia	rs10758593	9	4292083	A	G	0.965	4.9E-09
Insomnia	rs7402939	15	99183876	T	C	0.965	5.19E-09
Insomnia	rs2838787	21	46539725	A	G	0.965	7.65E-09
Insomnia	rs6702604	1	107190062	A	G	0.964	1.3E-09
Insomnia	rs823247	2	2850540	T	C	0.964	5.25E-10
Insomnia	rs1519102	2	66677816	C	G	0.964	0.000000019
Insomnia	rs1064213	2	198950240	A	G	0.964	6.41E-10
Insomnia	rs7599697	2	239231477	T	C	0.964	0.000000005
Insomnia	rs2388840	6	99598756	A	G	0.964	1.37E-09
Insomnia	rs7475916	10	77771194	C	G	0.964	6.7E-09
Insomnia	rs4767645	12	118385788	T	G	0.964	6.47E-10
Insomnia	rs6510033	19	30710785	A	G	0.964	4.66E-08
Insomnia	rs623025	1	201765094	T	C	0.963	3.16E-08
Insomnia	rs73163783	3	117602144	T	C	0.963	1.39E-08
Insomnia	rs10944696	6	94498850	A	G	0.963	7.99E-09
Insomnia	rs6973090	7	102008352	A	G	0.963	4.31E-08
Insomnia	rs671985	8	60914783	A	G	0.963	2.79E-10
Insomnia	rs11001276	10	76825638	A	T	0.963	2.52E-08
Insomnia	rs214934	11	17193475	A	T	0.963	3.16E-09
Insomnia	rs6589988	11	99126016	A	G	0.963	4.7E-09

Insomnia	rs8181889	13	53613990	A	G	0.963	8.9E-10
Insomnia	rs1536053	13	111982291	T	C	0.963	6.04E-09
Insomnia	rs3184470	16	715164	A	G	0.963	9.73E-10
Insomnia	rs8076183	17	61024696	T	C	0.963	2.75E-10
Insomnia	rs1937447	1	66358242	C	G	0.962	2.08E-08
Insomnia	rs7571486	2	176473295	A	G	0.962	0.000000014
Insomnia	rs4502882	5	153093998	T	C	0.962	7.96E-10
Insomnia	rs6457796	6	34828553	T	C	0.962	1.12E-08
Insomnia	rs4090240	9	77118987	T	C	0.962	8.46E-09
Insomnia	rs12251016	10	21821918	A	T	0.962	3.89E-10
Insomnia	rs224029	10	64519299	T	C	0.962	2.51E-10
Insomnia	rs566673	11	66401373	T	G	0.962	1.18E-10
Insomnia	rs10502966	18	50748499	A	G	0.962	8.54E-11
Insomnia	rs7615602	3	18718055	C	G	0.961	2.59E-09
Insomnia	rs521484	7	49894349	A	G	0.961	1.53E-08
Insomnia	rs75932578	7	106844694	T	C	0.961	4.15E-08
Insomnia	rs12790660	11	57667222	T	C	0.961	4.49E-10
Insomnia	rs2389631	13	96932868	A	C	0.961	2.03E-10
Insomnia	rs2089358	1	37194103	T	C	0.96	2.75E-10
Insomnia	rs1289939	1	117944435	T	C	0.96	0.000000006
Insomnia	rs11803128	1	190060095	A	G	0.96	6.85E-11
Insomnia	rs6545798	2	60521311	A	T	0.96	1.19E-11
Insomnia	rs4664299	2	160570033	T	C	0.96	4.95E-09
Insomnia	rs3774751	3	50209053	T	G	0.96	7.32E-12
Insomnia	rs7044885	9	81739348	C	G	0.96	5.67E-12
Insomnia	rs6734957	2	42813247	T	G	0.959	1.82E-09
Insomnia	rs62301574	4	22050165	C	G	0.959	1.37E-08
Insomnia	rs12917449	15	74331659	A	C	0.959	2.97E-08
Insomnia	rs9889282	17	50259142	A	C	0.959	4.7E-12
Insomnia	rs11126082	2	66789341	C	G	0.958	8.26E-13
Insomnia	rs984306	2	66817402	T	C	0.958	7.94E-10
Insomnia	rs314281	6	105400605	T	C	0.958	6.03E-13
Insomnia	rs10761240	9	96361922	A	G	0.958	2.12E-12

Insomnia	rs12912299	15	38897857	T	C	0.958	4.42E-13
Insomnia	rs4238755	16	52746089	A	C	0.958	2.3E-10
Insomnia	rs60565673	18	52906830	T	G	0.958	1.59E-12
Insomnia	rs12983032	19	5073447	A	G	0.958	1.07E-11
Insomnia	rs694786	3	173112907	T	C	0.957	1.97E-13
Insomnia	rs17083297	5	92995477	A	C	0.957	0.000000016
Insomnia	rs3131638	6	31475127	A	G	0.957	7.88E-10
Insomnia	rs6967168	7	132672192	T	G	0.957	1.39E-10
Insomnia	rs524859	11	66041079	A	G	0.957	1.48E-12
Insomnia	rs61921611	12	66367726	T	C	0.957	7.84E-12
Insomnia	rs7214267	17	43157709	A	G	0.957	5.09E-13
Insomnia	rs61765555	1	73957815	T	C	0.956	4E-11
Insomnia	rs11605348	11	47606483	A	G	0.956	7.01E-13
Insomnia	rs16990210	4	34720226	T	C	0.955	1.97E-08
Insomnia	rs12540241	7	3824141	A	T	0.955	1.58E-09
Insomnia	rs10947690	6	37631768	A	G	0.954	4.04E-12
Insomnia	rs4702	15	91426560	A	G	0.953	6.78E-16
Insomnia	rs73079014	3	49863483	T	C	0.952	3.65E-08
Insomnia	rs8180817	7	114047542	C	G	0.952	1.83E-16
Insomnia	rs76145129	20	62670427	T	G	0.951	2.73E-08
Insomnia	rs1031654	13	54382035	A	C	0.95	3.88E-12
Insomnia	rs152555	5	106849674	A	G	0.949	4.83E-10
Insomnia	rs2431108	5	103947968	T	C	0.948	7.83E-17
Insomnia	rs9394502	6	38452503	T	C	0.947	7.76E-18
Insomnia	rs4709655	6	163280204	T	C	0.947	3.09E-09
Insomnia	rs28582096	12	123856998	A	G	0.947	1.74E-13
Insomnia	rs4981170	14	33412996	A	G	0.947	7.33E-13
Insomnia	rs72657797	4	90820809	T	C	0.946	1.52E-12
Insomnia	rs8180457	5	107209814	T	C	0.946	1.12E-11
Insomnia	rs73671843	7	3520024	A	G	0.946	5.49E-10
Insomnia	rs17324524	23	133134421	T	C	0.944	5.01E-10
Insomnia	rs13010288	2	51824512	T	G	0.942	9.26E-12
Insomnia	rs62383308	5	165460085	A	G	0.942	3.98E-08

Insomnia	rs17643634	8	91650818	T	C	0.942	1.34E-13
Insomnia	rs66674044	16	19904344	A	T	0.942	2.18E-12
Insomnia	rs6119267	20	31163914	C	G	0.942	2.32E-20
Insomnia	rs492858	3	155432229	T	C	0.936	3.46E-09
Insomnia	rs10947428	6	33647058	T	C	0.934	9.06E-21
Insomnia	rs79693059	11	72340686	C	G	0.93	1.61E-11
Insomnia	rs11838830	13	60362013	A	G	0.923	5.2E-10
Insomnia	rs4699157	4	106055212	T	C	0.922	3.98E-08
Insomnia	rs7432782	3	48941551	T	C	0.92	7.42E-09
Insomnia	rs13135092	4	103198082	A	G	0.915	2.53E-16
Insomnia	rs17520265	7	119674508	A	G	0.913	2.87E-08
Insomnia	rs78206187	2	67022234	A	G	0.91	2.96E-13
Insomnia	rs117630493	13	54018867	C	G	0.904	3.61E-08
Insomnia	rs138678612	6	30932223	A	G	0.89	1.41E-08
