# Supplementary material for: Observational and Genetic Associations of Modifiable Risk Factors with Aortic Valve Stenosis: A Prospective Cohort Study of 0.5 Million Participants
Source: Nutrients. 2022 May 28;14(11):2273. doi: 10.3390/nu14112273 (PMC9182826; doi:10.3390/nu14112273)
Supplement: Supplementary file 1 [file nutrients-14-02273-s001.zip › supplement table1.pdf]

**Table S1.** List of data of modifiable risk factors using for creating genetic risk scores, including metabolic factors, biochemical index, education, lifestyle factors. *.

| Modifiable risk factor | Sample size | No of SNPs | Year | Reference |
| --- | --- | --- | --- | --- |
| **Metabolic factors** | | | | |
| BMI | 339224 | 97 | 2015 | Locke AE, Kahali B, Berndt SI, et al. |
| BF | 100716 | 12 | 2016 | Lu Y, Day FR, Gustafsson S, et al. |
| WHR | 224459 | 49 | 2015 | Shungin D, Winkler TW, Croteau-Chonka DC, et al. |
| SBP | 99785 | 23 | 2017 | Hoffmann TJ, Ehret GB, Nandakumar P, et al. |
| PP | 99785 | 38 | 2017 | Hoffmann TJ, Ehret GB, Nandakumar P, et al. |
| RHR | 181171 | 21 | 2013 | den Hoed M, Eijgelsheim M, Esko T, et al. |
| **Biochemical index** | | | | |
| HbA1c | 46368 | 10 | 2010 | Soranzo N, Sanna S, Wheeler E, et al. |
| Vitamin D | 42024 | 4 | 2013 | Vimaleswaran KS, Berry DJ, Lu C, et al. |
| Triglyceride | 188578 | 28 | 2013 | Willer CJ, Schmidt EM, Sengupta S, et al. |
| High-density lipoprotein | 188578 | 60 | 2013 | Willer CJ, Schmidt EM, Sengupta S, et al. |
| Low-density lipoprotein | 188578 | 30 | 2013 | Willer CJ, Schmidt EM, Sengupta S, et al. |
| Serum total cholesterol | 188578 | 39 | 2013 | Willer CJ, Schmidt EM, Sengupta S, et al. |
| Urate | NA | 71 | 2018 | Lyon MS, Andrews SJ, Elsworth B, et al. |
| C-reactive protein | 204402 | 150 | 2018 | Lyon MS, Andrews SJ, Elsworth B, et al. |
| Creatinine | 327525 | 28 | 2017 | Lyon MS, Andrews SJ, Elsworth B, et al. |
| Albumin | 382500 | 60 | 2018 | Lyon MS, Andrews SJ, Elsworth B, et al. |
| **Education** | | | | |
| Education of years | 293723 | 74 | 2016 | Okbay A, Beauchamp JP, Fontana MA, et al. |
| **Lifestyle factors** | | | | |
| Cigarettes smoked per day | 337334 | 55 | 2019 | Liu M, Jiang Y, Wedow R, et al. |
| Smoking initiation | 1E+06 | 378 | 2019 | Liu M, Jiang Y, Wedow R, et al. |
| Smoking cessation | 547219 | 24 | 2019 | Liu M, Jiang Y, Wedow R, et al. |

| | | | | |
|---|---|---|---|---|
| Coffee consumption | 118329 | 5 | 2015 | Coffee and Caffeine Genetics Consortium, Cornelis MC, Byrne EM, et al. |
| Morningness | 434835 | 207 | 2019 | Jansen PR, Watanabe K, Stringer S, et al. |
| Sleep duration | 384317 | 49 | 2019 | Jansen PR, Watanabe K, Stringer S, et al. |
| Ease of getting up | 385949 | 62 | 2019 | Jansen PR, Watanabe K, Stringer S, et al. |
| Napping | 386577 | 7 | 2019 | Jansen PR, Watanabe K, Stringer S, et al. |
| Daytime dozing | 386548 | 1 | 2019 | Jansen PR, Watanabe K, Stringer S, et al. |
| Snoring | 359916 | 36 | 2019 | Jansen PR, Watanabe K, Stringer S, et al. |
| Insomnia | 1E+06 | 245 | 2019 | Jansen PR, Watanabe K, Stringer S, et al. |

SNP = single nucleotide polymorphism; MR = Mendelian randomization; BMI = body mass index; BF = body fat percentage; WHR = waist-to-hip ratio; SBP = systolic blood pressure; PP = pulse pressure; TG = triglyceride; HDL = high-density lipoprotein; LDL = low-density lipoprotein; TC = serum total cholesterol; RHR = resting heart rate; HbA1c = glycated hemoglobin. Detailed reference can be found and described in Methods.
