# Supplementary material for: Observational and Genetic Associations of Modifiable Risk Factors with Aortic Valve Stenosis: A Prospective Cohort Study of 0.5 Million Participants
Source: Nutrients. 2022 May 28;14(11):2273. doi: 10.3390/nu14112273 (PMC9182826; doi:10.3390/nu14112273)
Supplement: Supplementary file 1 [file nutrients-14-02273-s001.zip › supplement table3.pdf]

Table S3. Phenotypic variables of modifiable risk factors used in observational association and MR analysis in UK biobank.

Modifiable risk factor	Selected phenotypes	Phenotypes sample size	Field ID	Calculated methods
**Metabolic factors**				
BMI	Body mass index (kg/m^2)	360808	21001	Original
BF	Body fat percentage (%)	355548	23099	Original
WHR	Waist circumference, Hip circumference (cm)	361293	48, 49	Waist circumference/Hip circumference*100%
SBP	Systolic blood pressure (mmHg)	337959	4080, 93	Original
PP	SBP, DBP (mmHg)	337959	4080, 93, 4079, 94	SBP-DBP
RHR	Pulse rate (bpm)	337966	102	Original
**Biochemical index**				
HbA1c	Glycated haemoglobin (HbA1c) (mmol/mol)	345067	30750	Original
Vitamin D	Blood vitamin D (nmol/L)	330301	30890	Original
Triglyceride	Triglycerides (mmol/L)	344960	30870	Original
High-density lipoprotein	High-density lipoprotein (mmol/L)	316079	30760	Original
Low-density lipoprotein	Low-density lipoprotein (mmol/L)	344589	30780	Original
Serum total cholesterol	Cholesterol (mmol/L)	345227	30690	Original
Urate	Urate (umol/L)	344812	30880	Original
C-reactive protein	C-reactive protein (mg/L)	344502	30710	Original
Creatinine	Creatinine (mmol/L)	345059	30700	Original
Albumin	Albumin (g/L)	316218	30600	Original
**Education**				
Education of years	Age completed full time education (years)	245480	845	Original
**Lifestyle factors**				

Cigarettes smoked per day	Cigarettes per day (number)	108017	2345, 2887	Original
Smoking initiation	Smoking status	361930	20116	Smoking present or previous
Smoking cessation	Smoking status	161949	20116	Smoking previous but not present
Coffee consumption	Coffee intake (cups/day)	335702	1498	Original
Morningness	Morning/evening person (Chronotype)	322940	1180	Original
Sleep duration	Sleep duration (hours/day)	360011	1160	Original
Ease of getting up	Getting up in morning	361165	1170	Original
Napping	Nap during day	361800	1190	Original
Daytime dozing	Daytime dozing / sleeping (narcolepsy)	360747	1220	Original
Snoring	Snoring	337526	1210	Original
Insomnia	Sleeplessness / insomnia	361688	1200	Original

SNP = single nucleotide polymorphism; MR = Mendelian randomization; BMI = body mass index; BF = body fat percentage; WHR = waist-to-hip ratio; SBP = systolic blood pressure; PP = pulse pressure; TG = triglyceride; HDL = high-density lipoprotein; LDL = low-density lipoprotein; TC = serum total cholesterol; RHR = resting heart rate; HbA1c = glycated hemoglobin.
