# Supplementary material for: Observational and Genetic Associations of Modifiable Risk Factors with Aortic Valve Stenosis: A Prospective Cohort Study of 0.5 Million Participants
Source: Nutrients. 2022 May 28;14(11):2273. doi: 10.3390/nu14112273 (PMC9182826; doi:10.3390/nu14112273)
Supplement: Supplementary file 1 [file nutrients-14-02273-s001.zip › supplement table4.pdf]

Table S4. Hazard ratio of modifiable risk factors and AVS incident patients.

Modifiable risk factor	Model1	Model2	Model3
**Metabolic factors**			
BMI	1.08(1.07,1.09) **	1.07(1.06,1.08) **	1.06(1.05,1.07) **
BF	1.05(1.04,1.06) **	1.04(1.03,1.05) **	1.04(1.03,1.05) **
WHR	1.04(1. 03,1.05) **	1.04(1.03,1.04) **	1.03(1.03,1.04) **
SBP	1.10(1.08,1.13) **	1.10(1.07,1.13) **	1.10(1.07,1.13) **
PP	1.23(1.19,1.27) **	1.22(1.19,1.26) **	1.23(1.19,1.26) **
RHR	1.10(1.05,1.14) **	1.09(1.04,1.13) **	1.06(1.02,1.10) **
**Biochemical index**			
HbA1c	1.07(1.06,1.08) **	1.07(1.06,1.08) **	1.07(1.06,1.08) **
Vitamin D	0.94(0.91,0.96) **	0.94(0.92,0.96) **	0.96(0.93,0.98) **
Triglyceride	1.10(1.05,1.15) **	1.08(1.04,1.13) **	1.04(0.99,1.10)
High-density lipoprotein	0.77(0.65,0.91) **	0.82(0.69,0.96) *	0.85(0.71,1.01)
Low-density lipoprotein	1.13(1.06,1.21) **	1.13(1.06,1.21) **	1.10(1.03,1.18) **
Serum total cholesterol	1.07(1.02,1.13) **	1.07(1.02,1.13) **	1.05(1.00,1.11)
Urate	1.03(1.02,1.03)**	1.03(1.02,1.03)**	1.02(1.02,1.03)**
C-reactive protein	1.03(1.02,1.04)**	1.03(1.02,1.04)**	1.03(1.02,1.04)**
Creatinine	1.04(1.02,1.05)**	1.03(1.02,1.05)**	1.04(1.03,1.05) **
Albumin	0.93(0.91,0.95)**	0.93(0.91,0.95)**	0.92(0.9,0.94)**
**Education**			
Education of years	0.94(0.92,0.97) **	0.96(0.93,0.99) *	0.96(0.93,0.99) *
**Lifestyle factors**			
Cigarettes smoked per day	1.13(1.07,1.20) **	1.11(1.05,1.18) **	1.11(1.04,1.18) **
Smoking initiation	1.40(1.27,1.55) **	1.33(1.20,1.47) **	1.35(1.21,1.50) **
Smoking cessation	0.75(0.64,0.87) **	0.80(0.68,0.93) **	0.76(0.65,0.90) **
Coffee consumption	1.05(1.03,1.07) **	1.05(1.03,1.07) **	1.04(1.02,1.07) **
Morningness	0.95(0.86,1.06)	0.95(0.85,1.06)	1.01(0.90,1.13)
Sleep duration	1.00(0.96,1.05)	1.01(0.96,1.05)	1.00(0.96,1.05)
Ease of getting up	0.68(0.59,0.78) **	0.69(0.61,0.79) **	0.72(0.62,0.83) **
Napping	1.37(1.24,1.51) **	1.32(1.19,1.46) **	1.28(1.15,1.43) *
Daytime dozing	1.14(1.02,1.27)*	1.10(0.99,1.23)	1.11(0.99,1.24)
Snoring	1.07(0.97,1.19)	1.07(0.97,1.19)	1.04(0.93,1.16)
Insomnia	1.25(1.10,1.41)**	1.20(1.06,1.36)**	1.19(1.05,1.35)**

Model1: sex, age;
Model2: model1 + family history of cardiovascular disease (yes or no), family history of diabetes (yes or no), education status (college or university degree, A levels/AS levels or equivalent, O levels/GCSEs or equivalent, CSEs or equivalent, NVQ or HND or HNC or equivalent, other professional qualifications), household income (less than 18,000 pounds per year (£/y), 18,000 to 29,999 £/y, 30,000 to 51,999 £/y, 52,000 to 100,000 £/y, more than 100,000 £/y), and Townsend deprivation index.
Model3: model2 + metabolic equivalent of physical activity (METs) , alcohol daily consumption (grams), smoking status (never smoking, previous smoking, current smoking) and systolic blood pressure (mmHg).
Blood biochemistry were further adjusted by cholesterol drug using.

* P value less than 0.05 ($P < 0.05$); ** P value less than 0.0018 ($P < 0.05/28$) for Bonferroni correction.
