# Supplementary material for: Observational and Genetic Associations of Modifiable Risk Factors with Aortic Valve Stenosis: A Prospective Cohort Study of 0.5 Million Participants
Source: Nutrients. 2022 May 28;14(11):2273. doi: 10.3390/nu14112273 (PMC9182826; doi:10.3390/nu14112273)
Supplement: Supplementary file 1 [file nutrients-14-02273-s001.zip › supplement table5.pdf]

Table S5. Adjusted Hazard Ratios for AVS events by gender and age.

	No. of events	Person years	HR (95% CI)							
			Body mass index	Body fat percentage	Triglyceride	Low-density lipoprotein	Serum total cholesterol	Cigarettes smoked per day	Ease of getting up	Insomnia
Sex										
Male	1048	4466	1.07(1.05,1.08) *	1.03(1.02,1.04) *	1.03(0.97,1.09)	1.05(0.96,1.14)	1.03(0.96,1.10)	1.01(1.00,1.02) *	0.94(0.86,1.03)	1.09(1.00,1.29) *
female	554	3004	1.06(1.04,1.07) *	1.04(1.03,1.06) *	1.06(0.96,1.16)	1.17(1.05,1.31) *	1.09(1.00,1.19)	1.02(1.01,1.04) *	0.82(0.74,0.91) *	1.28(1.12,1.46) *
Age										
< 45 years	14	86	1.05(0.96,1.15)	1.06(0.97,1.15)	1.43(1.03,1.99) *	2.76(1.67,4.55) *	2.11(1.42,3.12) *	0.97(0.86,1.10)	1.03(0.51,2.08)	1.53(0.71,3.27)
45-49 years	41	241	1.08(1.03,1.14) *	1.03(0.98,1.09)	1.12(0.89,1.40)	1.58(1.08,2.31) *	1.48(1.11,1.97) *	1.02(0.98,1.06)	0.64(0.44,0.94) *	1.26(0.80,1.98)
50-54 years	88	446	1.05(1.01,1.09) *	1.04(1.01,1.08) *	0.81(0.64,1.02)	1.20(0.91,1.57)	1.14(0.92,1.40)	1.01(0.98,1.04)	1.03(0.78,1.37)	1.08(0.79,1.47)
55-59 years	206	1066	1.06(1.04,1.09) *	1.04(1.02,1.07) *	1.10(0.98,1.24)	1.07(0.89,1.28)	1.00(0.87,1.15)	1.02(1.00,1.04) *	1.13(0.93,1.38)	1.19(0.97,1.46)
60-64 years	500	2458	1.05(1.04,1.07) *	1.03(1.01,1.04) *	0.92(0.84,1.02)	0.99(0.87,1.12)	0.97(0.89,1.07)	1.01(1.00,1.02)	0.81(0.72,0.91) *	1.15(1.01,1.30) *
≥65 years	753	3172	1.07(1.05,1.09) *	1.04(1.02,1.05) *	1.11(1.03,1.19) *	1.10(0.99,1.22) *	1.05(0.97,1.14) *	1.01(1.00,1.02) *	0.90(0.81,1.00) *	1.12(1.01,1.24) *

Results were adjusted by sex, age family history of cardiovascular disease (yes or no), family history of diabetes (yes or no), education status (college or university degree, A levels/AS levels or equivalent, O levels/GCSEs or equivalent, CSEs or equivalent, NVQ or HND or HNC or equivalent, other professional qualifications), household income (less than 18,000 pounds per year (£/y), 18,000 to 29,999 £/y, 30,000 to 51,999 £/y, 52,000 to 100,000 £/y, more than 100,000 £/y), Townsend deprivation index, metabolic equivalent of physical activity (METs), alcohol daily consumption (grams), smoking status (never smoking, previous smoking, current smoking) and systolic blood pressure (mmHg).
