# Supplementary material for: Observational and Genetic Associations of Modifiable Risk Factors with Aortic Valve Stenosis: A Prospective Cohort Study of 0.5 Million Participants
Source: Nutrients. 2022 May 28;14(11):2273. doi: 10.3390/nu14112273 (PMC9182826; doi:10.3390/nu14112273)
Supplement: Supplementary file 1 [file nutrients-14-02273-s001.zip › supplement table6.pdf]

Table S6. Adjusted hazard ratios for AVS by quintiles of baseline and predicted variants.

	Baseline					
	**Quintile1**	**Quintile2**	**Quintile3**	**Quintile4**	**Quintile5**	**P for trend**
**Body mass index**						
No. of participants	72,026	71,917	71,861	71,780	71,628	
No. of person years	601032	587748	575072	563243	541560	
No. of events	147	183	270	299	392	
Model 1	1(ref)	1.07(0.87,1.31)	1.39(1.15,1.68) *	1.53(1.27,1.85) *	2.33(1.95,2.78) *	<0.001
Model 2	1(ref)	1.07(0.87,1.31)	1.39(1.15,1.68) *	1.52(1.15,1.68) *	2.28(1.90,2.72) *	<0.001
Model 3	1(ref)	1.06(0.87,1.30)	1.39(1.15,1.68) *	1.52(1.26,1.83) *	2.28(1.90,2.73) *	<0.001
Model 4	1(ref)	1.00(0.82,1.23)	1.25(1.03,1.52) *	1.30(1.07,1.58) *	1.83(1.50,2.23) *	<0.001
Model 5	1(ref)	1.01(0.82,1.25)	1.29(1.05,1.58) *	1.41(1.15,1.73) *	2.00(1.62,2.46) *	<0.001
**Body fat percentage**						
No. of participants	71,301	70,471	70,990	70,879	70,350	
No. of person years	577969	556424	559446	575153	565630	
No. of events	234	270	264	229	266	
Model 1	1(ref)	1.19(1.01,1.39) *	1.34(1.14,1.58)*	1.76(1.46,2.13)*	2.71(2.19,3.36)*	<0.001
Model 2	1(ref)	1.16(0.99,1.36)	1.29(1.10,1.52)*	1.70(1.41,2.06)*	2.57(2.07,3.20)*	<0.001
Model 3	1(ref)	1.18(1.01,1.39)*	1.33(1.12,1.57)*	1.70(1.40,2.06)*	2.62(2.10,3.26)*	<0.001
Model 4	1(ref)	1.06(0.90,1.24)	1.09(0.92,1.30)	1.30(1.06,1.60)*	1.86(1.46,2.36)*	<0.001
Model 5	1(ref)	1.11(0.94,1.31)	1.11(0.92,1.33)	1.40(1.13,1.74)*	2.03(1.58,2.61)*	<0.001
**Triglyceride**						
No. of participants	68,931	68,614	68,753	68,614	68,528	
No. of person years	566863	552952	548729	540298	534531	
No. of events	164	213	232	297	322	
Model 1	1(ref)	1.01(0.84,1.21)	0.96(0.81,1.16)	1.15(0.97,1.36)	1.21(1.02,1.43)*	<0.001
Model 2	1(ref)	0.99(0.82,1.18)	0.95(0.79,1.13)	1.11(0.94,1.32)	1.17(1.94,1.32)*	<0.001
Model 3	1(ref)	0.99(0.83,1.19)	0.95(0.79,1.14)	1.12(0.94,1.33)	1.17(0.99,1.39)*	<0.001

Model 4	1(ref)	0.95(0.79,1.14)	0.87(0.73,1.05)	1.00(0.84,1.19)	1.01(0.85,1.21)	<0.001
Model 5	1(ref)	0.97(0.80,1.17)	0.87(0.72,1.05)	0.99(0.83,1.19)	0.98(0.82,1.18)	<0.001
**Low-density lipoprotein**						
No. of participants	68,522	68,641	68,666	68,615	68,623	
No. of person years	485213	549773	566120	569580	569717	
No. of events	303	214	229	221	263	
Model 1	1(ref)	0.91(0.78,1.06)	1.07(0.91,1.27)	1.07(0.90,1.28)	1.30(1.09,1.56)*	<0.001
Model 2	1(ref)	0.92(0.79,1.07)	1.09(0.93,1.29)	1.09(0.93,1.29)	1.31(1.10,1.57)*	<0.001
Model 3	1(ref)	0.91(0.78,1.07)	1.08(0.91,1.28)	1.06(0.89,1.27)	1.30(1.09,1.55)*	<0.001
Model 4	1(ref)	0.92(0.78,1.07)	1.08(0.91,1.28)	1.05(0.87,1.26)	1.27(1.06,1.52)*	<0.001
Model 5	1(ref)	0.91(0.77,1.07)	1.12(0.94,1.33)	1.04(0.86,1.26)	1.31(1.09,1.58)*	<0.001
**Serum total cholesterol**						
No. of participants	68,598	68,818	68,724	68,807	68,758	
No. of person years	481888	552176	567496	571785	572079	
No. of events	309	226	221	224	250	
Model 1	1(ref)	1.01(0.87,1.18)	1.05(0.89,1.24)	1.13(0.95,1.35)	1.26(1.06,1.51)*	<0.001
Model 2	1(ref)	1.04(0.89,1.20)	1.08(0.91,1.27)	1.16(0.97,1.38)	1.28(1.07,1.53)*	<0.001
Model 3	1(ref)	1.03(0.88,1.20)	1.06(0.89,1.25)	1.14(0.96,1.36)	1.26(1.05,1.51)*	<0.001
Model 4	1(ref)	1.04(0.89,1.21)	1.07(0.90,1.27)	1.15(0.96,1.37)	1.26(1.05,1.51)*	<0.001
Model 5	1(ref)	1.07(0.91,1.26)	1.11(0.93,1.32)	1.17(0.97,1.41)	1.29(1.06,1.56)*	<0.001
**Cigarettes smoked per day**						
No. of participants	29,543	20,449	36,090	6,203	15,045	
No. of person years	235692	158811	273403	45608	104192	
No. of events	133	123	235	52	144	
Model 1	1(ref)	1.27(1.00,1.63)	1.26(1.02,1.56)*	1.51(1.10,2.09)*	1.50(1.18,1.91)*	<0.001
Model 2	1(ref)	1.27(0.99,1.62)	1.29(1.04,1.60)*	1.53(1.11,2.12)*	1.54(1.21,1.98)*	<0.001
Model 3	1(ref)	1.26(0.98,1.61)	1.26(1.02,1.57)*	1.52(1.10,2.10)*	1.49(1.17,1.91)*	<0.001
Model 4	1(ref)	1.22(0.96,1.57)	1.19(0.96,1.48)	1.39(1.01,1.93)*	1.33(1.04,1.70)*	<0.001
Model 5	1(ref)	1.22(0.94,1.58)	1.20(0.96,1.51)	1.29(0.91,1.82)	1.31(1.01,1.69)*	<0.001

**Ease of getting up**						
No. of participants	241,688	117,878	/	/	/	
No. of person years	491406	2377998	/	/	/	
No. of events	767	525	/	/	/	
Model 1	1(ref)	0.94(0.85,1.04)	/	/	/	<0.001
Model 2	1(ref)	0.97(0.87,1.07)	/	/	/	<0.001
Model 3	1(ref)	0.96(0.86,1.06)	/	/	/	<0.001
Model 4	1(ref)	0.96(0.87,1.07)	/	/	/	<0.001
Model 5	1(ref)	0.97(0.87,1.08)	/	/	/	<0.001
**Insomnia**						
No. of participants	87,342	274,346	/	/	/	
No. of person years	705566	2168728	/	/	/	
No. of events	274	1,019	/	/	/	
Model 1	1(ref)	1.21(1.07,1.37)*	/	/	/	<0.001
Model 2	1(ref)	1.20(1.06,1.35)*	/	/	/	<0.001
Model 3	1(ref)	1.21(1.07,1.37)*	/	/	/	<0.001
Model 4	1(ref)	1.20(1.06,1.35)*	/	/	/	<0.001
Model 5	1(ref)	1.19(1.05,1.36)*	/	/	/	<0.001

**Prediction**						
	**Quintile1**	**Quintile2**	**Quintile3**	**Quintile4**	**Quintile5**	**P for trend**
**Body mass index**						
No. of participants	72,040	71,943	71,847	71,769	71,613	
No. of person years	604734	590512	577387	561054	534969	
No. of events	108	186	254	317	426	
Model 1	1(ref)	1.28(1.03,1.60)*	1.47(1.18,1.82)*	1.53(1.23,1.89)*	1.62(1.30,2.01)*	<0.001
Model 2	1(ref)	1.28(1.02,1.60)*	1.45(1.17(1.80)*	1.50(1.21,1.87)*	1.57(1.27,1.96)*	<0.001
Model 3	1(ref)	1.26(1.00,1.57)*	1.44(1.16,1.78)*	1.49(1.20,1.86)*	1.58(1.27,1.97)*	<0.001
Model 4	1(ref)	1.24(0.99,1.56)	1.41(1.13,1.75)*	1.44(1.16,1.79)*	1.51(1.21,1.88)*	<0.001
Model 5	1(ref)	1.33(1.05,1.68)*	1.43(1.14,1.86)*	1.48(1.18,1.86)*	1.57(1.25,1.98)*	<0.001

**Body fat percentage**						
No. of participants	71,166	70,315	70,617	71,298	70,595	
No. of person years	590242	518029	563959	600156	562235	
No. of events	120	431	285	101	358	
Model 1	1(ref)	1.36(1.07,1.74)*	1.57(1.20,2.05)*	1.88(1.01,3.51)*	3.19(1.67,6.12)*	<0.001
Model 2	1(ref)	1.33(1.05,1.70)*	1.53(1.17,2.01)*	1.82(0.97,3.40)	3.11(1.62,5.96)*	<0.001
Model 3	1(ref)	1.35(1.05,1.72)*	1.54(1.17,2.03)*	1.79(0.95,3.37)	3.06(1.58,5.91)*	<0.001
Model 4	1(ref)	1.33(1.04,1.70)*	1.52(1.16,1.99)*	1.74(0.92,3.27)	2.92(1.51,5.64)*	<0.001
Model 5	1(ref)	1.34(1.04,1.73)*	1.48(1.11,1.96)*	1.53(0.80,2.93)	2.43(1.23,4.78)*	<0.001
**Triglyceride**						
No. of participants	68,855	68,778	68,693	68,653	68,461	
No. of person years	578849	567605	553847	532911	510161	
No. of events	122	191	255	302	425	
Model 1	1(ref)	1.14(0.93,1.41)	1.25(1.01,1.54)*	1.13(0.90,1.42)	1.33(1.06,1.68)*	<0.001
Model 2	1(ref)	1.14(0.92,1.40)	1.25(1.01,1.54)*	1.14(0.90,1.43)	1.33(1.05,1.68)*	<0.001
Model 3	1(ref)	1.14(0.92,1.40)	1.24(1.00,1.53)	1.10(0.87,1.38)	1.32(1.04,1.67)*	<0.001
Model 4	1(ref)	1.13(0.91,1.39)	1.22(0.99,1.51)	1.08(0.86,1.36)	1.31(1.03,1.65)*	<0.001
Model 5	1(ref)	1.13(0.91,1.41)	1.17(0.94,1.46)	1.02(0.80,1.30)	1.29(1.01,1.65)*	<0.001
**Low-density lipoprotein**						
No. of participants	68,671	68,619	68,583	68,622	68,572	
No. of person years	549035	546044	547518	548277	549527	
No. of events	221	241	294	239	300	
Model 1	1(ref)	1.11(0.95,1.31)	1.30(1.10,1.52)*	1.21(1.02,1.44)*	1.54(1.30,1.83)*	<0.001
Model 2	1(ref)	1.12(0.95,1.32)	1.30(1.10,1.53)*	1.23(1.03,1.45)*	1.56(1.31,1.85)*	<0.001
Model 3	1(ref)	1.08(0.92,1.28)	1.29(1.10,1.52)*	1.19(1.01,1.41)*	1.51(1.28,1.30)*	<0.001
Model 4	1(ref)	1.09(0.92,1.28)	1.30(1.11,1.53)*	1.20(1.02,1.43)*	1.53(1.29,1.82)*	<0.001
Model 5	1(ref)	1.05(0.88,1.24)	1.31(1.11,1.55)*	1.19(1.00,1.42)	1.52(1.28,1.82)*	<0.001
**Serum total cholesterol**						
No. of participants	68,771	68,618	68,711	68,847	68,758	

No. of person years	546952	521019	546613	571905	558936	
No. of events	240	333	279	183	260	
Model 1	1(ref)	1.00(0.86,1.16)	1.14(0.97,1.34)	1.32(1.00,1.73)	1.46(1.09,1.94)*	<0.001
Model 2	1(ref)	0.99(0.85,1.15)	1.14(0.96,1.34)	1.34(1.02,1.76)*	1.48(1.11,1.97)*	<0.001
Model 3	1(ref)	0.99(0.85,1.16)	1.13(0.95,1.33)	1.32(1.00,1.75)*	1.48(1.11,1.98)*	<0.001
Model 4	1(ref)	0.99(0.85,1.15)	1.13(0.95,1.33)	1.33(1.01,1.76)*	1.48(1.10,1.98)*	<0.001
Model 5	1(ref)	1.03(0.88,1.20)	1.13(0.95,1.34)	1.36(1.02,1.82)*	1.48(1.09,2.00)*	<0.001
**Cigarettes smoked per day**						
No. of participants	21,554	21,497	21,511	21,419	21,349	
No. of person years	180827	171116	169432	154337	141994	
No. of events	50	106	93	184	254	
Model 1	1(ref)	0.91(0.64,1.29)	0.97(0.65,1.46)	1.12(0.63,1.99)	1.01(0.56,1.82)	<0.001
Model 2	1(ref)	0.91(0.64,1.29)	0.96(0.64,1.44)	1.11(0.63,1.97)	0.99(0.55,1.79)	<0.001
Model 3	1(ref)	0.95(0.67,1.37)	1.04(0.69,1.57)	1.18(0.66,2.10)	1.07(0.59,1.93)	<0.001
Model 4	1(ref)	0.95(0.66,1.36)	1.03(0.68,1.55)	1.16(0.65,2.07)	1.05(0.58,1.89)	<0.001
Model 5	1(ref)	0.87(0.61,1.26)	0.88(0.57,1.36)	0.95(0.53,1.73)	0.87(0.47,1.60)	<0.001
**Ease of getting up**						
No. of participants	180,329	179,237	/	/	/	
No. of person years	1526801	1342603	/	/	/	
No. of events	228	1067	/	/	/	
Model 1	1(ref)	1.28(1.06,1.55)*	/	/	/	<0.001
Model 2	1(ref)	1.31(1.08,1.58)*	/	/	/	<0.001
Model 3	1(ref)	1.28(1.06,1.55)*	/	/	/	<0.001
Model 4	1(ref)	1.27(1.05,1.53)*	/	/	/	<0.001
Model 5	1(ref)	1.23(1.01,1.50)*	/	/	/	<0.001
**Insomnia**						
No. of participants	180,090	179,999	/	/	/	
No. of person years	1427032	1447262	/	/	/	
No. of events	606	687	/	/	/	

Model 1	1(ref)	1.29(1.12,1.48)*	/	/	/	<0.001
Model 2	1(ref)	1.28(1.12,1.47)*	/	/	/	<0.001
Model 3	1(ref)	1.30(1.13,1.49)*	/	/	/	<0.001
Model 4	1(ref)	1.29(1.12,1.48)*	/	/	/	<0.001
Model 5	1(ref)	1.27(1.10,1.47)*	/	/	/	<0.001

Model 1: adjusted for age, sex, cholesterol medical history (yes or no), household income(less than 18,000 pounds per year (£/y), 18,000 to 29,999 £/y, 30,000 to 51,999 £/y, 52,000 to 100,000 £/y, more than 100,000 £/y), education(college or university degree, A levels/AS levels or equivalent, O levels/GCSEs or equivalent, CSEs or equivalent, NVQ or HND or HNC or equivalent, other professional qualifications), and Townsend deprivation index.

Model 2: model 1 + Metabolic equivalent of physical activity (METs), smoking status (never smoking, previous smoking, current smoking), and alcohol daily consumption (grams).

Model 3: model 2 + fruit (servings/days, 1 serving=5 pieces), vegetable (servings/day, serving=3 tablespoons), fish (servings/week) and meat intakes (serving/week).

Model 4: model 3 + body mass index (kg/m^2), and waist-hip ratio.

Model 5: model 4 + diastolic blood pressure (mmHg), and systolic blood pressure(mmHg).
