# Supplementary material for: Observational and Genetic Associations of Modifiable Risk Factors with Aortic Valve Stenosis: A Prospective Cohort Study of 0.5 Million Participants
Source: Nutrients. 2022 May 28;14(11):2273. doi: 10.3390/nu14112273 (PMC9182826; doi:10.3390/nu14112273)
Supplement: Supplementary file 1 [file nutrients-14-02273-s001.zip › supplement table7.pdf]

Table S7. Adjusted hazard ratios per unit higher modifiable risk factors for sensitivity MR analyses of AVS.

Incidence AVS	No. of cases	No. of participants	HR(95%CI)							
			Body mass index	Body fat percentage	Triglyceride	Low-density lipoprotein	Serum total cholesterol	Cigarettes smoked per day	Insomnia	HbA1c
Excluding participants taking cholesterol-lowering medication at baseline	888	299,828	1.06(0.98,1.15)	2.19(0.81,5.96)	1.03(0.92,1.14)	1.12(1.02,1.22)*	1.03(0.89,1.18)	1.08(0.85,1.37)	1.41(1.14,1.75)*	1.16(1.03,1.30)*
Excluding first three years of follow-up	1,338	352,108	1.10(1.03,1.18)*	3.00(1.33,6.80)*	1.08(1.00,1.17)*	1.14(1.07,1.21)*	1.08(0.97,1.21)	1.12(0.91,1.37)	1.39(1.17,1.66)*	1.11(0.99,1.24)
Excluding all above	749	293,105	1.08(0.99,1.17)	3.65(1.23,10.77)*	1.06(0.95,1.19)	1.10(1.00,1.21)	0.99(0.85,1.16)	1.06(0.82,1.38)	1.41(1.11,1.79)*	1.18(1.04,1.34)*

Results were adjusted by sex, age family history of cardiovascular disease (yes or no), family history of diabetes (yes or no), education status (college or university degree, A levels/AS levels or equivalent, O levels/GCSEs or equivalent, CSEs or equivalent, NVQ or HND or HNC or equivalent, other professional qualifications), household income (less than 18,000 pounds per year (£/y), 18,000 to 29,999 £/y, 30,000 to 51,999 £/y, 52,000 to 100,000 £/y, more than 100,000 £/y), medication use of cholesterol-lowering and Townsend deprivation index. * P value less than 0.05 ($P < 0.05$).
