# Supplementary material for: Observational and Genetic Associations of Modifiable Risk Factors with Aortic Valve Stenosis: A Prospective Cohort Study of 0.5 Million Participants
Source: Nutrients. 2022 May 28;14(11):2273. doi: 10.3390/nu14112273 (PMC9182826; doi:10.3390/nu14112273)
Supplement: Supplementary file 1 [file nutrients-14-02273-s001.zip › supplement table8.pdf]

Table S8. Hazard ratio of modifiable risk factors and AVS incident patients.

Modifiable risk factor	Beta (SE)	
	**MR-Egger**	**Weighted median**
BMI	0.0019 (0.0005)*	0.0023 (0.0012)*
BF	0.0021 (0.0007)*	0.0050 (0.0003)*
TG	0.0022 (0.0031)	0.0043 (0.0014)*
LDL	0.0031 (0.0005)*	0.0078 (0.0022)*
TC	0.0017 (0.0004)*	0.0035 (0.0008)*
CPD	0.0011 (0.0025)	0.0013 (0.0021)
Insomnia	0.0035 (0.0008)*	0.0081 (0.0039)*

BMI=Body mass index; BF= Body fat percentage; TG= Triglyceride; LDL= Low-density lipoprotein; TC= Serum total cholesterol; CPD= Cigarettes consumption per day
* P value less than 0.05 (P <0.05);.
